# Standard vs. targeted oxygen therapy prehospitally for chronic obstructive pulmonary disease (STOP-COPD): study protocol for a randomised controlled trial

**DOI:** 10.1186/s13063-024-07920-5

**Published:** 2024-01-25

**Authors:** Arne Sylvester Rønde Jensen, Jan Brink Valentin, Mathilde Gundgaard Mulvad, Victor Hagenau, Søren Helbo Skaarup, Søren Paaske Johnsen, Ulla Væggemose, Martin Faurholdt Gude

**Affiliations:** 1 Department of Research & Development, Prehospital Emergency Medical Services, Central Denmark Region, Aarhus, Denmark; 2 Department of Ambulance & Physician Response Unit, Prehospital Emergency Medical Services, Central Denmark Region, Aarhus, Denmark; 3 Danish Center for Health Services Research, Department of Clinical Medicine, Aalborg University, Aalborg, Denmark; 4 Department of Respiratory Medicine and Allergy, Aarhus University Hospital, Aarhus, Denmark; 5 Department of Clinical Medicine, Aarhus University, Aarhus, Denmark; 6 Department of Anaesthesiology and Intensive Care, Aarhus University Hospital, Aarhus, Denmark

**Keywords:** Prehospital, Titrated oxygen, COPD, Acute exacerbation of COPD, Mortality, Emergency medical services, Emergency medical technicians, Paramedic

## Abstract

**Background:**

A high concentration of inspired supplemental oxygen may possibly cause hypercapnia and acidosis and increase mortality in patients with acute exacerbation of chronic obstructive pulmonary disease (AECOPD). Even so, patients with AECOPD are being treated with high oxygen flow rates when receiving inhalation drugs in the prehospital setting. A cluster-randomised controlled trial found that reduced oxygen delivery by titrated treatment reduced mortality—a result supported by observational studies—but the results have never been reproduced. In the STOP-COPD trial, we investigate the effect of titrated oxygen delivery compared with usual care consisting of high flow oxygen delivery in patients with AECOPD in the prehospital setting.

**Methods:**

In this randomised controlled trial, patients will be blinded to allocation. Patients with suspected AECOPD (*n* = 1888) attended by the emergency medical service (EMS) and aged > 40 years will be allocated randomly to either standard treatment or titrated oxygen, targeting a blood oxygen saturation of 88–92% during inhalation therapy. The trial will be conducted in the Central Denmark Region and include all ambulance units. The power to detect a 3% 30-day mortality risk difference is 80%. The trial is approved as an emergency trial. Hence, EMS providers will include patients without prior consent.

**Discussion:**

The results will provide evidence on whether titrated oxygen delivery outperforms standard high flow oxygen when used to nebulise inhaled bronchodilators in AECOPD treatment. The trial is designed to ensure unselected inclusion of patients with AECOPD needing nebulised bronchodilators—a group of patients that receives high oxygen fractions when treated in the prehospital setting where the only compressed gas is generally pure oxygen. Conducting this trial, we aim to improve treatment for people with AECOPD while reducing their 30-day mortality.

**Trial registration:**

European Union Clinical Trials (EUCT) number: 2022-502003-30-00 (authorised 06/12/2022), ClinicalTrials.gov number: NCT05703919 (released 02/02/2023), Universal trial number: U1111-1278-2162.

**Supplementary Information:**

The online version contains supplementary material available at 10.1186/s13063-024-07920-5.

## Administrative information

Note: the numbers in curly brackets in this protocol refer to SPIRIT checklist item numbers. The order of the items has been modified to group similar items (see http://www.equator-network.org/reporting-guidelines/spirit-2013-statement-defining-standard-protocol-items-for-clinical-trials/).

**Table:** 

Title {1}	Standard vs. targeted oxygen therapy prehospitally for chronic obstructive pulmonary disease (STOP-COPD): study protocol for a randomised controlled trial
Trial registration {2a and 2b}.	European Union Clinical Trials (EUCT) number: 2022-502003-30-00 ClinicalTrials.gov number: NCT05703919 Universal trial number: U1111-1278-2162
Protocol version {3}	v. 4.3 (supplemental document 1) Date 17/08/2023
Funding {4}	Den Landsdækkende Akutlægehelikopterordning (English language: “Danish Air Ambulance”), Simon Spies Fonden (English language: “the Simon Spies Foundation”), Eva Merete Crone Falck’s Fond (English language: “the Eva Merete Crone Falck’s Foundation”, Region Midtjyllands Strategiske forskningsmidler (English language: “Support from the Strategic Research Fund of the Central Denmark Region”. Further funding will be applied for.
Author details {5a}	1. Department of Research & Development, Prehospital Emergency Medical Services, Central Denmark Region, Aarhus, Denmark 2. Department of Ambulance & Physician Response Unit, Prehospital Emergency Medical Services, Central Denmark Region, Aarhus, Denmark 3. Danish Center for Health Services Research, Department of Clinical Medicine, Aalborg University, Aalborg, Denmark 4. Department of Respiratory Medicine and Allergy, Aarhus University Hospital, Denmark 5. Department of Clinical Medicine, Aarhus University, Aarhus, Denmark 6. Department of Anaesthesiology and Intensive Care, Aarhus University Hospital, Aarhus, Denmark
Name and contact information for the trial sponsor {5b}	Prehospital Emergency Medical Services, Central Denmark Region Olof Palmes Allé 34 1. sal, 8200 Aarhus N Denmark Phone: 0045 78414848 E-mail: hovedpostkasse@ph.rm.dk
Role of sponsor {5c}	The sponsor had no part in designing the study and will have no influence on the trial, e.g. its conduct, results, interpretation, final manuscript or decision to publish.

## Introduction

### Background and rationale {6a}

Worldwide, chronic obstructive pulmonary disease (COPD) is the third leading cause of death [1, 2]. In Denmark, its estimated prevalence is between 200,000 and 400,000 annual cases in a population of 5.8 million people [3, 4]. Patients with AECOPD have a high mortality rate, ranging from 5–10% (in-hospital mortality) [5, 6] to 9–16% (30-day mortality) [7, 8].

High concentrations of inspired supplemental oxygen may cause hypercapnia and acidosis and increase mortality in patients with AECOPD [9–12]. Even so, they are often treated with inappropriately high fractions of supplemental oxygen even when a blood oxygen saturation (SpO_2_) > 92% has been reached [6, 13]. This practice runs counter to guidelines recommending oxygen treatment titrated at a target SpO_2_ of 88–92% [14, 15].

A single cluster-randomised controlled trial (CRT) has been conducted to investigate titrated oxygen treatment in prehospital patients with suspected AECOPD [5]. In 405 included patients, intention-to-treat analysis demonstrated a statistically significantly reduced mortality (9% to 4%) favouring a titrated oxygen protocol over a high flow oxygen protocol. In the study, a reduced mortality (9% to 2%) was seen in the subgroup of patients with confirmed AECOPD, and, surprisingly, a reduced mortality was also seen in the group of patients without a final COPD diagnosis who had dyspnoea due to another aetiology (9% to 6%).

A cluster randomised trial (CRT) introduces the potential for bias and intra-cluster correlation. Furthermore, the patient cohort consisted of a limited number, wherein only half received an COPD diagnosis, and protocol violations were also observed. As far as we are aware, a robustly designed individual-level randomised controlled trial (RCT) to validate these findings has not been carried out.

Moreover, following these findings, there has been a reduction in the application of oxygen therapy, casting doubt on the impact of titrated oxygen for COPD/AECOPD. Consequently, there is a growing necessity for a pre-hospital randomised controlled trial (RCT) to establish the most effective oxygen approach for AECOPD. This requirement has been underscored in various studies, notably in a 2020 Cochrane review [6, 8, 16, 17]..

The aim of this RCT is to determine the effects of titrated oxygen versus standard oxygen in patients with suspected AECOPD and need of inhaled bronchodilators in the prehospital setting of the Central Denmark Region.

### Objectives {7}

The primary objective is to determine if a prehospital titrated oxygen strategy may reduce 30-day mortality compared with standard high-dose oxygen treatment in patients with suspected AECOPD (Table 1).

**Table 1:** Secondary objectives

To determine whether a prehospital titrated oxygen strategy for AECOPD patients will result in a reduced 24-h and 7-day mortality compared to patients receiving standard care	
To determine whether a prehospital titrated oxygen strategy for patients with AECOPD will result in reduced length of hospital and ICU stay compared with patients receiving standard care	
To determine whether a prehospital titrated oxygen strategy for patients with AECOPD will reduce the in-hospital need for non-invasive ventilation (NIV) or invasive ventilation compared with patients receiving standard care	
To determine whether a titrated oxygen strategy affects time from hospital admission to intensive care unit admission and time to treatment with non-invasive ventilation or invasive ventilation compared with standard care	
To determine whether a prehospital titrated oxygen strategy for patients with AECOPD will reduce the proportion of patients with respiratory acidosis (PaCO_2_ > 6.3 kPa AND pH < 7.35) and determine the degree of acidosis measured on arrival to hospital compared with patients receiving standard care	
To determine whether a prehospital titrated oxygen strategy for patients with AECOPD will affect experienced dyspnoea (rated on a scale from 0 to 10) compared with patients receiving standard care	
To determine if a titrated oxygen strategy will lower the readmission rate (within 30 days) compared with standard care	
To determine whether a titrated oxygen strategy will have an effect on time to readmission compared to standard treatment	
To determine whether a prehospital titrated oxygen strategy for patients with AECOPD reduces mortality (24 h, 7 days, and 30 days), acidosis, intensive care unit (ICU) admission rate, and need of assisted ventilation compared with patients receiving standard care analysed on a subgroup level based on prehospital transport time	

### Trial design {8}

STOP-COPD is an investigator-initiated, acute, interventional, prospective, 1:1 randomised, parallel-group, patient-blinded, single-centre superiority trial. The Danish Medical Research Ethical Committee approved the STOP-COPD trial as an acute trial; hence, consent before inclusion is not required but must be obtained later (EUCT number: 2022-502003-30-00). The study was also approved by The Danish Medicines Agency (EUCT number: 2022-502003-30-00). The protocol follows the SPIRIT guidelines (Standard Protocol Items: Recommendations for Interventional Trials), https://www.spirit-statement.org/.

## Methods: participants, interventions, and outcomes (Fig. 1)

### Study setting {9}

The trial will be conducted in the Central Denmark Region, which has approx. 1.3 m residents. The prehospital emergency medical services (EMS) in the region consist of one regional emergency medical dispatch centre and 70 ambulances manned by 600–700 EMS providers. The prehospital emergency medical services of the Central Denmark Region dispatch 2000–3000 ambulances to patients suspected of AECOPD annually. Based on Danish data from 2020, the 30-day mortality was 13% (95% confidence interval (CI): 11–14) in the Central Denmark Region [18]. The region has six hospitals capable of receiving and treating prehospital patients with AECOPD.

**Fig. 1:**
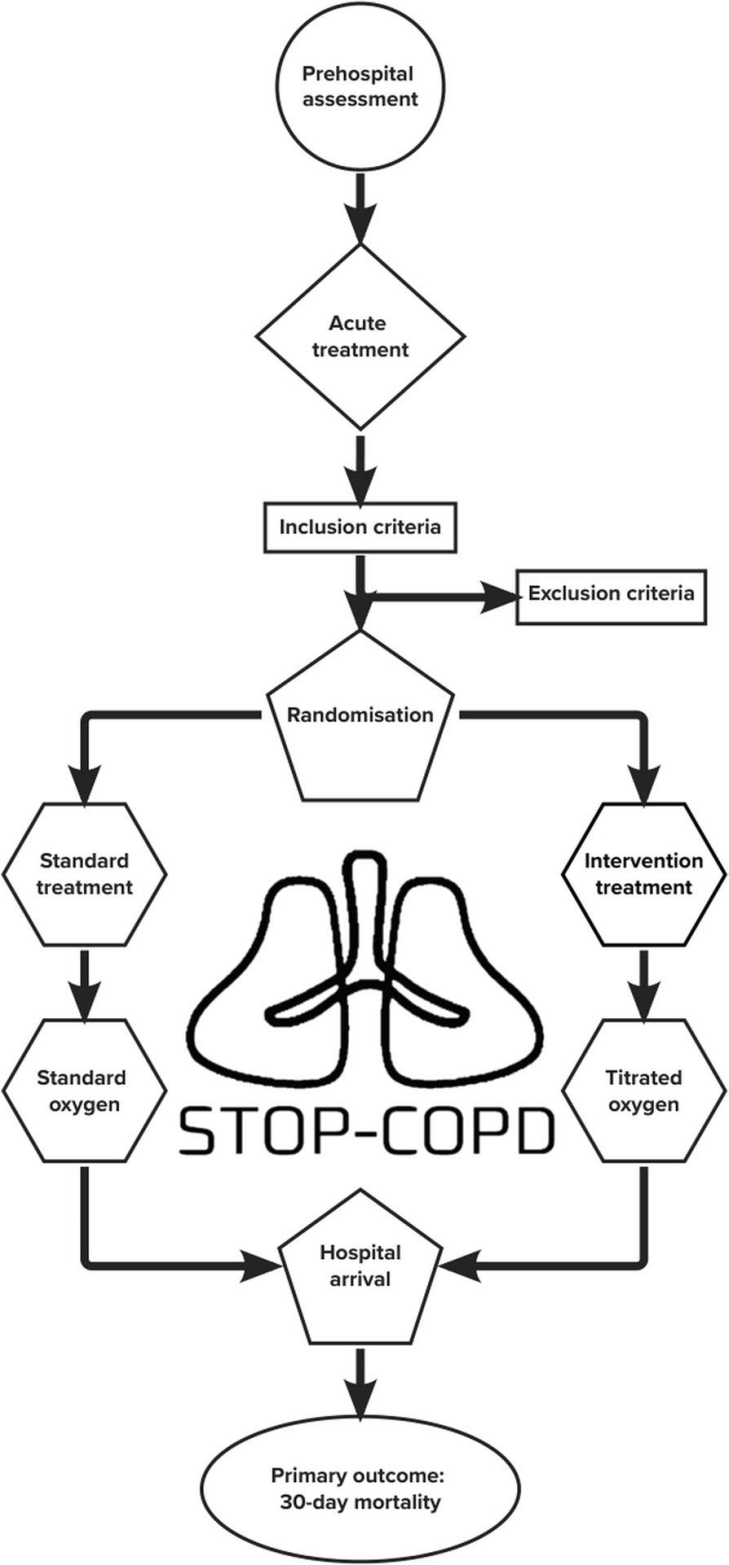
Trial flow

The EMS providers (emergency medical technicians and paramedics) will participate in the trial by identifying, including and treating eligible patients. Depending on the observed inclusion rate, the trial may be expanded to include two other regions of Denmark (the Region of Southern Denmark and the North Denmark Region).

### Eligibility criteria {10}

The eligibility criteria for inclusion are an age above 40 years, a prehospital need of inhaled bronchodilator treatment, and a suspicion of having AECOPD as determined by attending EMS providers. Confirmation of underlying COPD is also necessary for inclusion. Confirmation of COPD status can be acquired through either verbal affirmation of COPD (provided by the patient, family members, or caregivers present at the scene) or written confirmation of COPD with patient identification (from discharge letters, medical record notes, or medication lists) (Table 2).

**Table 2:** Inclusion and exclusion criteria

Inclusion criteria:	
1. Patients over the age of 40 years 2. Prehospital need of inhaled bronchodilator treatment 3. The treating emergency medical service (EMS) provider (emergency medical technician or paramedic) suspects acute exacerbation of chronic obstructive pulmonary disease (AECOPD) 4. Confirmation of the EMS provider’s suspicion of chronic obstructive pulmonary disease (COPD)	
Exclusion criteria:	
1. Non-COPD bronchospasm 2. Known or suspected pregnancy 3. Prehospital non-invasive ventilation (NIV), invasive ventilation or bag-mask-assisted ventilation 4. Allergy to inhalation drug (salbutamol) 5. Transfer between hospitals 6. Acute treatment by EMS providers with more than two doses (5 mg salbutamol) of inhalation drug before randomisation 7. Readmission within 30 days from a previous randomisation 8. Suspicion of acute coronary syndrome^a^	

^a^In concordance with the local standard operating procedure and based on: symptoms, electrocardiogram, trinitrotoluene (TnT), and medical consult

### Who will take informed consent? {26a}

**Fig. 2:**
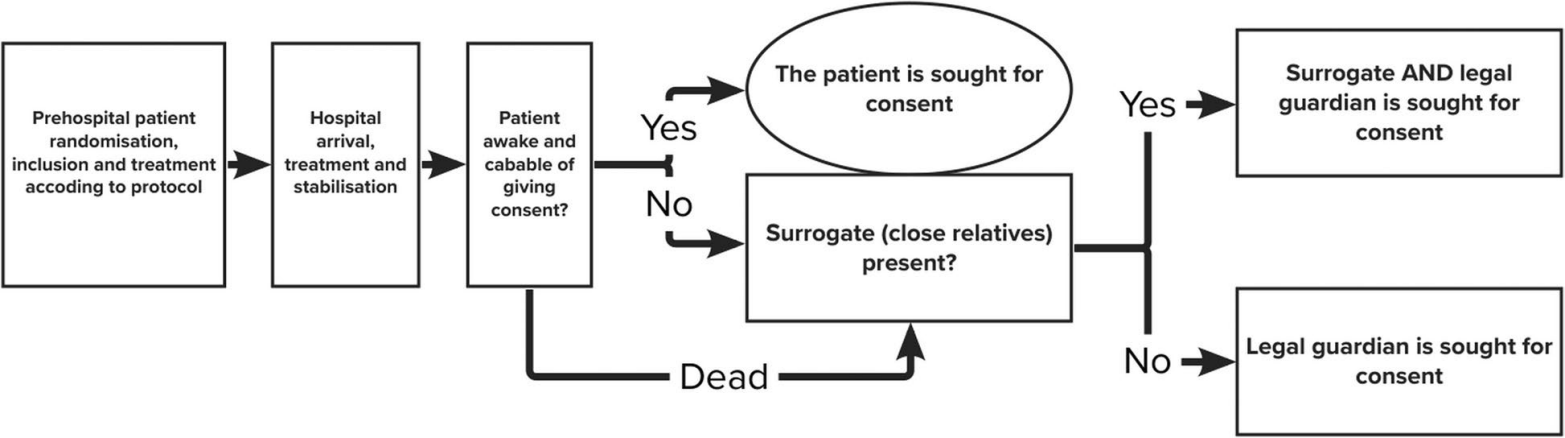
Consent collection flow. Consent will be obtained as soon as possible after hospital admission

AECOPD is associated with severe dyspnoea, anxiety, desperation, and decreased consciousness, especially in the prehospital phase of treatment. Hence, patients are often in a state in which they cannot receive and understand information, making informed consent to study participation impossible. Therefore, the STOP-COPD trial is approved as an acute trial by the Danish Medical Research Ethical Committee. Consent prior to inclusion and treatment is waived, and consent must therefore be obtained as soon as possible after hospital admission. Consent will be obtained by members of the research staff or associated physicians. Information about the trial will be provided in writing and verbally. Declining to give consent will in no way influence the subsequent treatment given to the patient. If a patient declines to give consent, data collection for the patient in question will be discontinued (Fig. 2).

### Additional consent provisions for collection and use of participant data and biological specimens {26b}

There are no plans for collection, laboratory evaluation, and storage of biological specimens for genetic or molecular analysis in the current trial and for future use in ancillary studies.

## Interventions

### Explanation for the choice of comparators {6b}

In this trial, titrated oxygen delivery aiming at a blood oxygen saturation (SpO_2_) of 88–92% is tested against standard high-dose oxygen. Hence, the comparator is standard high-dose oxygen where blood saturation is allowed to reach high levels (exceeding 92%).

### Intervention description {11a}

#### Standard treatment

Patients included in the standard (control) group will be treated with inhaled bronchodilators nebulised with 100% oxygen at a flow rate of 6–8 l/min without an upper SpO_2_ target. A bi-nasal end-tidal carbon dioxide (EtCO_2_) metre will be placed in the patient’s nose during nebulisation for EtCO_2_ measurement during treatment and transport, which also masks the patient for group allocation. The need of additional treatment will be assessed at the discretion of the treating EMS provider, according to local standard operating procedures (SOPs). When nebulised bronchodilators are not being delivered, the patient will receive supplemental oxygen according to local SOPs. At hospital arrival, the patient will have an arterial blood gas drawn and analysed within 30 min.

#### Intervention treatment

Patients included in the intervention group will be treated with inhaled bronchodilator nebulised with compressed atmospheric air (21% oxygen) at a flow rate of 6–8 l/min. A bi-nasal EtCO_2_ meter will be placed in the patient’s nose during nebulisation for EtCO_2_ measuring during treatment and while delivering supplemental oxygen, as needed. During nebulisation, oxygen will be titrated to achieve a SpO_2_ of 88–92%. The need for additional bronchodilator treatment will be assessed at the discretion of the treating EMS provider according to local SOPs. When nebulised bronchodilators are not being delivered, the patient will receive supplemental oxygen to achieve a SpO_2_ of 88–92%. At hospital arrival, the patient will have an arterial blood gas drawn and analysed within 30 min (Table 3).

**Table 3:** Various SpO_2_ scenarios

	Intervention treatment	Standard treatment
SpO_2_ < 88%	Supplemental oxygen via the EtCO_2_ meter up to 6 l/min. If higher oxygen levels are needed, oxygen will be used as driver for the nebuliser. If the SpO_2_ remains under 88%, additional oxygen may be added via the EtCO_2_ meter	Supplemental oxygen via the EtCO_2_ meter, if needed
SpO_2_ 88–92%	No supplemental oxygen	No supplemental oxygen
SpO_2_ >92%	No supplemental oxygen	No supplemental oxygen

### Criteria for discontinuing or modifying allocated interventions {11b}

In the intervention and control group alike, the SOP for requesting support from a physician-manned response unit is unchanged. In case of treatment failure with worsened clinical presentation, a prehospital physician may be requested according to the SOP. The prehospital physician then makes the decision on whether to discontinue the allocated treatment. Termination and reason for termination of treatment will be registered and used in the data analysis.

### Strategies to improve adherence to interventions {11c}

Twice weekly, members of the research staff will do real time audits on data base registrations with feedback to individual EMS providers in case of irregularities. The audits will focus on, firstly, the screening of all eligible patients and, secondly, EMS providers protocol adherence.

If suitable, patients are not screened for participation; the EMS providers will be contacted by the research staff as a reminder and to establish if any unforeseen inclusion problems have been encountered. Based on frequent audits and experience from previous studies, we expect a high recruitment rate. Protocol violations will be registered and reported in the final manuscript. The data monitoring committee (DMC) will conduct interim analyses, including protocol violations, on predefined milestones. If the DMC recommends measures to reduce the number of protocol violations, the trial steering committee will act accordingly.

### Relevant concomitant care permitted or prohibited during the trial {11d}

Enrolment will occur when the patient is physically present in the ambulance care area. Before the EMS provider and patient are present in the ambulance, initial assessment and immediate treatment of up to 5 mg of nebulized salbutamol using pure oxygen will be permitted. The EMS providers will be thoroughly trained to reduce this period to a minimum. Besides receiving the allocated trial treatment with different compositions of gases for nebulisation and target SpO_2_, the patients will receive the usual prehospital and in-hospital care at the full discretion of the treating EMS providers and in-hospital clinicians. However, patients are excluded when treated with non-invasive ventilation (NIV) or invasive ventilation by the physician-manned response unit prior to randomisation.

### Provisions for post-trial care {30}

There is no provision for post trial care apart from standard care. Trial participants will not be compensated for their participation. Participating patients in the trial are not expected to experience any harm. In the event that such harm occurs, trial participants are covered by the national Danish patient insurance scheme.

### Outcomes {12}

Primary and secondary outcomes are presented in Table 4. All outcome assessments will be completed on day 30 after randomisation. Patients who are readmitted will be registered as such, and their 30-day mortality will be determined from the first admission.

**Table 4:** Primary and secondary outcomes

Primary outcome:	
• **30-day mortality** presented as risk difference (RD) and risk ratio (RR) measured from randomisation. The outcome will be assessed using the electronic patient journal	
Secondary outcomes:	
• 24-h mortality and 7-day mortality presented as RD and RR measured from randomisation • Length of hospitalisation presented as days from randomisation to discharge • Intensive care unit (ICU) admission rate presented as RD and RR of enrolled patients admitted to ICU from randomisation to hospital discharge • Length of ICU stay as total number of days in ICU from randomisation to discharge presented as mean differences • In-hospital need for non-invasive ventilation (NIV) (at 24 h, 7 days, and 30 days) presented as RD and RR measured from randomisation • In-hospital need for invasive ventilation (at 24 h, 7 days, and 30 days) presented as RD and RR measured from randomisation • Time to in-hospital NIV presented as Aalen-Johansen curves and hazard ratio (HR) measured in days from randomisation • Time to in-hospital invasive ventilation presented as Aalen-Johansen curves and hazard ratio measured in days from randomisation • Proportion of patients with acidosis on arrival to hospital presented as RD and RR measured on in-hospital arterial blood gas (ABG) drawn < 30 min from hospital arrival • Degree of acidosis on ABG (pH) presented as mean or median differences • Patient-experienced dyspnoea score (verbal rating scale 0–10) measured before intervention treatment and at hospital arrival presented as median differences • Readmission rate from day 2 to day 30 after discharge presented as RD and RR • Time to readmission from discharge to day 30 presented as Arlen-Johnson curves and HR	

### Participant timeline {13}

**Fig. 3:**
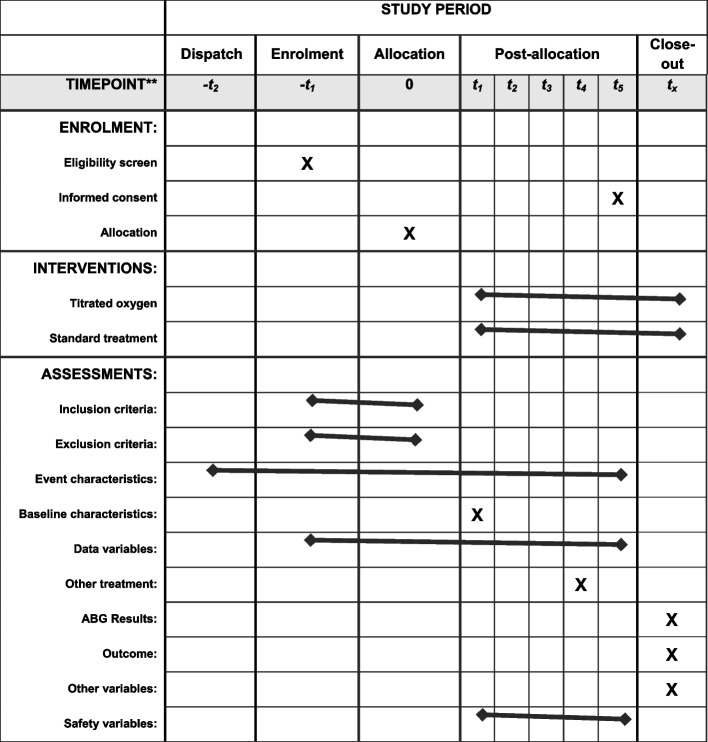
Timeline. “**” indicates the following: time points: -t2 = first contact to dispatch; -t1 = patient contact and assessment of eligibility; 0 = enrolment and randomisation; t1 = baseline prehospital variables and start of allocated treatment; t2 = continues treatment and monitoring; t3 = transport to hospital with ongoing treatment and monitoring; t4 = arrival at hospital, end of prehospital treatment and last registration of prehospital variables; t5 = colleting informed consent; tx = collecting hospital ABG results and patient outcomes. Inclusion criteria is shown in Table 2. Exclusion criteria is shown in Table 2. Event characteristics are as follows: date and time; CRN number; event number; ambulance ID; Dansk Index code; dispatch grade; dispatch time; EMS response time; arrival at patient time; time on scene; departure time; transport time; hospital arrival time; handover time; request by GP or dispatch; EMT or paramedic. Baseline characteristics are as follows: sex; age; known COPD; COPD severity; home oxygen; home NIV; earlier AECOPD; current smoker; limited treatment level; comorbidities; heart disease and its severity. Data variables are as follows: SpO_2_; EtCO_2_; ventilatory rate; pulse rate; systolic blood pressure; diastolic blood pressure; patient experienced dyspnoea; GCS; temperature. Other treatment are as follows: acute treatment before randomisation; prehospital steroids; beta-2 I.V/I.M/S.C; doses of beta-2 inhaled; doses of anticholinergic inhaled. ABG results are as follows: PaO_2_; PaCO_2_; pH; lactate; base excess. Outcomes are shown in Table 4. Other variables are as follows: new treatment limit; hospital diagnosis. Safety variables are as follows: untreated hypoxia; SpO_2_ < 88%; SUSAR; SUSAR description; prehospital termination. Abbreviations: ABG, arterial blood gas; AECOPD, acute exacerbation of chronic obstructive pulmonary disease; COPD, chronic obstructive pulmonary disease; PRU, physician response unit; GCS, Glasgow Coma Score; GP, general practitioner; NIV, non-invasive ventilation; SUSAR, suspected unexpected serious adverse reaction

The participant timeline is shown in Fig. 3.

### Sample size {14}

The sample size estimate for the STOP-COPD trial is based on results from the CRT by Austin et al. [5]. An absolute risk reduction of 5% for in-hospital mortality was found in the intention-to-treat analysis in favour of a restricted titrated oxygen treatment to patients with suspected AECOPD.

However, the STOP-COPD trial differs from the CRT by Austin et al. with respect to study setup. In the CRT, not all included patients were treated with inhaled bronchodilators, and the control group received higher oxygen concentrations than prescribed in current standard practice. Furthermore, a larger uncertainty is associated with a CRT setup as a result of reduced power. In addition, a limited number of clusters increases the risk of cluster-specific confounding, considering administrative differences between sites, etc. To accommodate for these differences, we translated the findings in the CRT by Austin et al. to our study by using a conservative estimate of a 3% reduced (risk difference) (from 7% in the control arm to 4% in the intervention arm) 30-day mortality in favour of titrated oxygen treatment. At a power of 80%, an expected drop out of max. 4%, and a significance level of 5%, the total required sample size will be 1888 patients—944 in each treatment arm. Due to the uncertainty of the estimated risk reduction, a sample size re-estimation is planned. When data have been collected for the first 500 patients, a re-estimation of the sample size will be made based on the observed risk difference seen in this interim analysis. A new sample size from the re-estimation will be considered in terms of its clinical relevance and feasibility and forwarded as a recommendation from the DMC.

### Recruitment {15}

All patients treated with nebulised salbutamol in the ambulances will be screened for study participation using a randomisation smartphone app (see the ‘Sequence generation {16a}’ section).

## Assignment of interventions: allocation

### Sequence generation {16a}

Patients will be randomised in a 1:1 ratio. A randomised block design will be utilised, using receiving hospital, sex, and age group (above/below 70 years of age) as block factors. Each block will be of random size comprising four, six, or eight patients. The patients will be randomised by the EMS providers attending the patients using a smartphone app supported by The Clinical Trial Unit, Aarhus University, Denmark. This app will be accessible from smartphones or tablets and requires no login information besides the receiving hospital’s ID. In the app, the following items of information are requested: receiving hospital, patients’ civil registration number, and inclusion/exclusion criteria. The randomisation sequence incorporating the block design will be a computer-generated random allocation list performed by an independent data manager using Stata (StataCorp. 2021. Stata Statistical Software: Release 17. College Station, TX: StataCorp LLC) and will not be accessible to the clinicians responsible for patient enrolment. The EMS providers will not have access to any information on the randomisation sequence.

### Concealment mechanism {16b}

Proper concealment of randomisation was obtained by the use of an external randomisation service (Clinical Trial Unit, Dept. of Clinical Medicine, Aarhus University, Denmark) providing an allocation process without human involvement. Allocation lists were generated by use of a validated procedure in the statistical package Stata (StataCorp. 2021. Stata Statistical Software: Release 17. College Station, TX: StataCorp LLC). The EMS providers will not have access to any information on the randomisation sequence.

### Implementation {16c}

N/A. See the ‘Sequence generation {16a}’ section.

## Assignment of interventions: blinding

### Who will be blinded {17a}

The trial will be single blinded with patients blinded to treatment allocation. The EMS providers will be instructed to keep the patients blinded, but they will not themselves be blinded. A protocol for emergency unblinding is unnecessary.

### Procedure for unblinding if needed {17b}

The design is open label with only patients and in-hospital staff being blinded. EMS providers attending the patients and trial staff members will not be blinded. We do not anticipate any requirement for unblinding, but if required, the trial staff members will have access to group allocations and any unblinding will be reported.

## Data collection and management

### Plans for assessment and collection of outcomes {18a}

The first data will be collected by the EMS providers during randomisation, including the civil registration number and data on admission hospital and inclusion/exclusion criteria. After obtaining informed consent and 30 days after randomisation, all other data will be collected from the electronic patient record and registered in the electronic case report form by trained research staff. One hundred days after randomisation, a safety follow-up will be made searching the electronic patient record for late registration of death.

All EMS providers involved in the treatment of enrolled patients will be trained to optimise data quality and validity. All data will be entered into the electronic case report form according to a data dictionary created prior to patient enrolment. The electronic case report form will be tested and validated before the trial is initiated.

### Plans to promote participant retention and complete follow-up {18b}

If a patient is discharged before consent is obtained, the patient will be contacted by telephone to arrange a physical meeting or a video call where full information on study participation will be given. Furthermore, the patient is given the opportunity to raise questions and signed written informed consent may be agreed upon.

### Data management {19}

All baseline and follow-up data will be collected from the electronic prehospital patient record and electronic patient record by trained research staff and entered into the electronic case report form in Research Electronic Data Capture (REDCap). The electronic case report form will comprise safety ranges and data entry validation rules ensuring correct data entry.

### Confidentiality {27}

All confidential data on the included patients will be stored in REDCap—both the electronic case report forms and the signed electronic consents. In REDCap, the data will be stored during the full inclusion period and will be exported only for analysis. Data analysis will be performed on the data exported from REDCap and stored in a secure electronic data base (MidtX) hosted by the Central Denmark Region according to European Union regulations [19]. Data will be handled according to all relevant Danish and European Union provisions, including the General Data Protection Regulation and the Data Protection Act [20, 21]. The project will be registered with the internal register of research projects of the Central Denmark Region, with permission from the Danish Data Protection Agency. Each patient will receive a unique trial identification number. During the trial, the sponsor, investigator, research staff, and coordinator will have access to the entire databases (REDCap and MidtX). The Good Clinical Practice unit, regulatory agencies, and other relevant entities will have direct access to patient records and to all relevant trial data including the electronic case report form, as applicable. The DMC does not have direct access to data; instead, specified analysed data will be shared according to the charter for the DMC; additional data may be shared on request.

### Plans for collection, laboratory evaluation, and storage of biological specimens for genetic or molecular analysis in this trial/future use {33}

There are no plans for collection, laboratory evaluation, and storage of biological specimens for genetic or molecular analysis in the current trial and for future use in ancillary studies.

## Statistical methods

### Statistical methods for primary and secondary outcomes {20a}

Differences in mortality are calculated as RD and RR, performed using linear regression and Poisson regression, respectively, with robust variance estimation [22–24]. For the primary analysis, the estimation is a crude analysis. All results will be presented with 95% CI. All binary secondary outcomes will be analysed in the same way as the primary outcome. Time-to-event outcomes will be analysed using Cox proportional hazards regression and Tobit regression. Continuous outcomes will be analysed using linear regression. The patient-experienced dyspnoea score will be analysed using linear mixed effects models.

### Interim analyses {21b}

Interim analyses will be made on predefined milestones (i.e. after 200, 500, 1000 and 1500 enrolled patients) once follow-up data have been collected. The results from the interim analyses will be presented only to the DMC who can recommend immediate trial discontinuation for reasons of futility or harm based on the following criteria: (1) patients have a statistically significantly higher risk of death in one treatment arm than in the other (1% significance level), and (2) patients have a significantly higher risk of safety issues (untreated hypoxia defined as repeated measures of SpO_2_ lower than 88% for a duration of ≥ 5 min after allocation—the significance level will be set at the discretion of the DMC).

### Methods for additional analyses (e.g. subgroup analyses) {20b}

The following subgroup analyses will be made: (1) primary and secondary outcome for groups defined by pulse-oximetry-measured blood saturation (< 88%, 88–92%, and > 92%) determined prior to the first administration of inhaled bronchodilators; (2) primary and secondary outcomes using prehospital transport time as a regression variable; (3) primary and secondary outcomes analysed on patient groups defined by a final diagnosis of AECOPD (yes/no); and (4) primary and secondary outcomes analysed by patient groups defined by in-hospital NIV and invasive ventilation.

If statistical analysis and methods that are not described in the protocol are deemed useful and important in the reporting of results, this will be stated clearly in the main article.

### Methods in analysis to handle protocol non-adherence and any statistical methods to handle missing data {20c}

All analyses will be made on an intention-to-treat basis. Patients with missing data on the primary outcome will be excluded. Sensitivity analyses will be made using multiple-imputation-chained equations with 100 imputation sets and including relevant first- and second-order variables in the imputation model [25]. Possible differences in patient characteristics and exposure between complete cases and dropouts are addressed by sensitivity analyses adjusted by appropriate patient characteristics using inverse probability of treatment weights (IPTW). Balanced diagnostics are conducted using the threshold criteria given by Zhang et al. [26].

### Plans to give access to the full protocol, participant-level data, and statistical code {31c}

All trial-related documents, including the protocol, will be publicly available at the trial website. De-identified participant-level data will be made available upon reasonable request. Anonymised data will be stored for 25 years.

## Oversight and monitoring

### Composition of the coordinating centre and trial steering committee {5d}

The coordinating centre is placed at the research unit at the Prehospital Emergency Medical Services in the Central Denmark Region. The day-to-day management of the trial will be handled by the trial coordinator and the principal investigator in collaboration. The trial steering committee consists of the principal investigator, the trial coordinator, and clinical experts in prehospital emergency medicine and pulmonary medicine. The steering committee will oversee the trial by reviewing and approving any study protocol modifications and by reviewing trial progress. The steering committee will gather after the DMC has held their scheduled meetings and discuss DMC recommendations. In case of unexpected events, the steering committee will gather at short notice.

### Composition of the data-monitoring committee, its role and reporting structure {21a}

The DMC will be responsible for safeguarding the interests of the trial participants and for assessing the safety and efficacy of the interventions during the trial. Also, the DMC is responsible for monitoring the overall conduct of the trial. The DMC consists of three specialists with expertise in anaesthesiology, intensive care, and clinical research and thus holds clinical and statistical expertise as recommended [27]. The DMC is independent of the sponsor and other members of the research staff. The DMC will review de-identified data for safety at five predetermined milestones (200, 500, 1000, and 1500 enrolled patients), but can, at any time, require extra reviews. Unless group differences are observed that require unblinding (as determined by the DMC), the DMC will be blinded to treatment groups. The trial will continue while the DMC reviews data. After a review, the DMC will prepare a short report for the steering committee with recommendations for continuation, modifications, or termination of the trial. The final decision on potential modifications or termination will rest with the steering committee and the sponsor-investigator. A detailed charter for the DMC will be available on the STOP-COPD trail website after patient inclusion starts.

### Adverse event reporting and harms {22}

During data entry into the electronic case reports forms (performed twice weekly), the electronic prehospital patient record will be screened for adverse events by research staff and any findings will be registered. Adverse events reported to by EMS providers, prehospital physicians, in-hospital clinicians, patients, or relatives will be registered by the sponsor-investigator. The sponsor-investigator will classify the events as one of the following: adverse events, adverse reactions, serious adverse events, serious adverse reactions, or suspected unexpected adverse reactions according to the list of adverse reactions in the summary of product characteristics. Suspected unexpected serious adverse events will be reported to the Good Clinical Practise unit, which will report to the EudraVigilance database. Due to the short half-life of the intervention and control drug, adverse events will be registered only if occurring in the prehospital phase. The reporting will be according to legislation and guidelines [19, 28].

### Frequency and plans for auditing trial conduct {23}

The Good Clinical Practice unit from Aarhus University will monitor and audit the trial. Before patient enrolment, a detailed monitoring plan will be prepared in collaboration between the Good Clinical Practice unit and the sponsor-investigator. The Good Clinical Practice unit will audit the following on all enrolled patients: primary outcome, consent, inclusion/exclusion criteria, and adverse events. Additionally, 10% of all enrolled patients will have a complete audit comprising all collected data points.

### Plans for communicating important protocol amendments to relevant parties (e.g. trial participants, ethical committees) {25}

Changes to the protocol will be decided on steering committee meetings and reported to the European Union clinical trials unit according to current legislation [19, 28]. Changes affecting the inclusion and exclusion criteria, interventions, and randomisation will be conveyed thoroughly to the prehospital clinicians (EMS providers and prehospital physicians).

### Dissemination plans {31a}

Following the CONSORT guidelines, the trial results will be published regardless of any negative, inconclusive or positive results [29, 30]. The results will be published in an international peer-reviewed journal with open access and disseminated as conference presentations. If the results from the trial have public interest, they will also be presented to mainstream media. The trial results will be shared with the participating EMS providers, patients, and others at the trial website (www.stop-copd.com). Within 1 year after the trial concludes, the results will be uploaded to the European Union Clinical Trials database [19, 31]. Authorship will follow the International Committee of Medical Journal Editors guidelines [32].

## Discussion

The need for improvements in the treatment of AECOPD is pressing as mortality has remained persistently high for many years. Currently, treatment with bronchodilators cannot be separated from high-dose oxygen because pure oxygen is the only available compressed gas for nebulisation in ambulances. With the STOP-COPD trial, we aim to investigate if titrated oxygen therapy aiming at achieving a SpO_2_ of 88–92% may reduce mortality in a group of patients treated with nebulised bronchodilators—a group of patients with moderate to severe AECOPD that receives high-dose oxygen during their ambulance transport due to their need for bronchodilator treatment.

We find our study setup to be a solid representation of the real workflow when EMS providers attend patients with AECOPD in the prehospital environment, but the setup also has some limitations. To make inclusion of patients feasible and clinically realistic, inclusion is first completed in the ambulance care area. Unfortunately, this allows for treatment with pure-oxygen-nebulised bronchodilators prior to randomisation during the transport time from the patient’s location to the ambulance care area. In the perfect setting, it should also be possible to use compressed atmospheric air for nebulisation at the patients’ location at first contact. Furthermore, EMS providers will not be blinded to the intervention. However, it would not be practical or safe to have unknown compressed gases in the ambulances where patients with non-COPD are being treated.

If we find beneficial effects of titrating oxygen, this would constitute a long-awaited improvement in the care of patients with AECOPD. Moreover, we consider titrated oxygen to be safe, easy to implement and inexpensive, making it a highly relevant treatment protocol. Furthermore, titrating oxygen has the potential to ease the burden on the healthcare system by reducing the severity of AECOPD.

## Trial status

As per January, 2024, the current protocol version and date is v4.3 August 17, 2023. The trial is not actively recruiting but is expected to start recruiting in the spring of 2025. The last day of patient recruiting will be Marts 30, 2027.

### Supplementary Information

**Additional file 1.**

## Abbreviations

AECOPD: Acute exacerbation of chronic obstructive pulmonary disease
CI: Confidence interval
COPD: Chronic obstructive pulmonary disease
CRT: Cluster-randomised controlled trial
DMC: Data monitoring committee
EMS: Emergency medicine services
EtCO_2_: End-tidal carbon dioxide
EUCT: European Union Clinical Trials
HR: Cox proportional hazard ratio
ICU: Intensive care unit
ID: Identification
NIV: Non-invasive ventilation
pH: Potential of hydrogen
RD: Risk difference
REDCap: Research Electronic Data Capture
RCT: Randomised controlled trial
RR: Risk ratio
SOP: Standard operating procedure
SpO_2_: Blood oxygen saturation
