# Supplementary material for: Standard vs. targeted oxygen therapy prehospitally for chronic obstructive pulmonary disease (STOP-COPD): study protocol for a randomised controlled trial
Source: Trials. 2024 Jan 25;25:85. doi: 10.1186/s13063-024-07920-5 (PMC10809561; doi:10.1186/s13063-024-07920-5)
Supplement: Supplementary file 1 — Additional file 1. [file 13063_2024_7920_MOESM1_ESM.pdf]

Supplemental document 1.

STOP COPD

Standard vs Targeted Oxygen Therapy Prehospital

for Chronic Obstructive Pulmonary Disease

Acronym: STOP-COPD

**Project name**	Standard vs Targeted Oxygen Therapy Prehospital for Chronic Obstructive Pulmonary Disease
**Project Acronym**	**STOP-COPD S**tandard vs **T**argeted **O**xygen Therapy **P**rehospital for **C**hronic **O**bstructive **P**ulmonary **D**isease
**Sponsor-Investigator**	Martin Faurholdt Gude, MD<br>Prehospital Emergency Medical Services (Præhospitalet), Olof Palmes Allé 34 1. sal, 8200 Aarhus N
**Sponsor-Investigator signature**	
**Study coordinator**	Arne Sylvester Rønde Jensen, Paramedic
**Universal trial number**	U1111-1278-2162
**Danish Medicines Agency ref. number**	2021070942
**EU CTIS number**	2022-502003-30-00
**Website (not active until trial start)**	www.STOP-COPD.com

STOP-COPD
Protocol
version 4.3
17-08-2023

Content

Abbreviations 7

0 List of modifications 9

1 Summary 11

2 Project overview 15

3 Background 16

3.1 Incidence and mortality 16

3.2 Pathophysiology 16

3.2.1 Hypoxic Pulmonary Vasoconstriction 16

3.2.2 The Haldane effect 17

3.2.3 Impaired respirator-drive in patients with COPD 17

3.3 Current evidence 18

3.4 Guidelines 19

3.5 Standard of care in Denmark 20

4 Trial objectives 20

5 Trial Design 21

5.1 Overview 21

5.1.1 Study procedures 21

5.2 Setting 22

5.3 Rollout 22

5.4 Outcomes 23

5.4.1 Primary Outcome 23

5.4.2 Secondary outcomes 23

5.5 Allocation 24

5.6 Randomization 24

5.7 Blinding 24

5.8 Study drugs 24

5.8.1 Investigational medicinal products 24

5.8.2 Auxiliary medicinal product 26

5.9 Trial phases 26

5.10 Termination 27

5.10.1 Termination of allocated treatment 27

5.10.2 Termination of the trial 27

5.11 Co-enrolment 27

5.12 Medical responsibility 28

5.13 Recruitment 28

5.14 End of trial 28

6 Inclusion and exclusion 28

6.1 Screening and enrolment 28

6.2 Inclusion criteria 29

6.3 Exclusion criteria 30

7 Intervention 30

7.1 Standard treatment 30

7.2 Intervention treatment 31

7.3 Clinical treatment 32

7.4 Clinical personnel 32

8 Data collection 32

8.1 Process 32

8.2 Variables 33

8.2.1 Baseline characteristics 33

8.2.2 Pre-intervention characteristics 34

8.2.3 Post-intervention characteristics 34

8.2.4 Outcomes ... 35
8.2.5 Safety ... 35
8.2.6 ABG ... 35
8.3 Patient-experienced dyspnoea ... 36
8.4 Data quality and validity ... 36
8.4.1 Protocol violations ... 36
9 Safety ... 37
9.1 Standard treatment ... 37
9.2 Intervention treatment ... 37
9.3 Adverse events and reactions ... 38
9.3.1 Definitions ... 38
9.3.2 Reporting ... 38
9.4 Specific adverse reactions ... 39
9.4.1 Oxygen (Medicinsk Oxygen ”Air Liquide”) ... 39
9.4.2 Compressed air (Medicinsk Luft ”Air Liquide”) ... 39
9.4.3 Salbutamol ... 40
9.4.4 Berodual ... 40
9.5 Assessment of adverse events ... 40
9.5.1 Timing ... 40
9.5.2 Classification of an event ... 40
10 Sample size and statistical analysis plan ... 40
10.1 Sample size calculation ... 40
10.2 Feasibility ... 41
10.2.1 Patients ... 41
10.2.2 Clinician’s enrolment rate ... 42
10.3 Stopping criteria ... 42

10.4 Statistical analysis plan 42
10.4.1 Outcomes and statistics 42
10.4.2 Subgroup analysis 44
10.5 Missing data 44
11 Data 44
11.1 Storage 44
11.2 Data access 45
11.3 Data sharing 45
12 Quality and monitoring 45
12.1 Good Clinical Practice monitoring 45
12.2 Data monitoring committee 46
13 Ethical Considerations 46
13.1 Risk/benefit assessment 46
13.1.1 Potential benefits 46
13.1.2 Potential harms 47
13.1.3 Risk/benefit ratio 47
13.2 Consent in emergency situations 47
13.2.1 Regulations from the European Parliament 48
13.2.2 Obtaining informed consent 50
13.2.3 Responsibilities regarding consent 51
13.2.4 Decline of consent 52
13.3 Summary of ethical considerations 52
13.4 Insurance 52
13.5 Approval from authorities 53
14 Funding 53
15 Timeline 54

16 Publication 55

17 Division of tasks 55

18 References 56

Appendices 59

Appendix 1 CONSORT flow diagram 59

Appendix 2 SOP Salbutamol 60

Appendix 3 SmPC Oxygen (Medicinsk Oxygen ”Air Liquide”) (In Danish) 63

Appendix 4 SmPC for Compressed air (Medicinsk Luft ”Air Liquide”) 81

Appendix 5 Marking of compressed air in ambulances 87

Appendix 6 Labelling of Oxygen 88

Appendix 7 Labelling of Air 89

Abbreviations

ABG: Arterial Blood Gas

ACS: Acute Coronary Syndrome

AECOPD: Acute Exacerbation of Chronic Obstructive Pulmonary Disease

AHF: Acute Heart Failure

EMDC: Emergency Medical Dispatch Center

BTS: British Thoracic Society

CI: Confidence Interval

COPD: Chronic Obstructive Pulmonary Disease

CRN: Civil Registration Number

CTR: REGULATION (EU) No 536/2014 OF THE EUROPEAN PARLIAMENT AND OF THE COUNCIL of 16 April 2014 on clinical trials on medicinal products for human use, and repealing Directive 2001/20/EC

DMC: Data monitoring committee

eCRF: electronic Case Report Form

ED: Emergency department

EMS: Emergency Medicine Services

EMT: Emergency Medical Technician

EPR: Electronic patient Record

GCP: Good Clinical Practice

Hb: Haemoglobin

HEMS: Helicopter Emergency Medical Services

ICU: Intensive Care Unit

IQR: Inter Quartile Range

MacCAT-CR: MacArthur Competence Assessment Tool for Clinical Research

MAT: Medical Laboratory Technologist

NIV: Non-Invasive Ventilation

$PaCO_2$: partial pressure of arterial dissolved carbon dioxide

PaO_2: Arterial Partial pressure of oxygen

PEMSCDR: Prehospital Emergency Medical Service of Central Denmark Region

PDSA: Paln-Do-Study-Act

PPR: Prehospital Patient Record

PRU: The physician response unit (rapid response emergency service, staffed by a senior anesthesiologist)

RedCAP: Research Electronic Data Capture

RCT: Randomized Controlled Trial

SD: Standard Deviation

SmPC: Summary of product characteristics (DK: Produktresume)

SOP: Standard operating procedure

SpO_2: Blood saturation

TMF: Trial master file

V/Q ratio: Ventilation/Perfusion ratio

0 List of modifications

Date of modification	Protocol version	Summary of modification	Explanation
12-07-2023	4.2	• New trial steering committee members p. 9-10	To strengthen the trial steering committee and the conduct of the trial
12-07-2023	4.2	• New funder added p. 54	Application for funding of the trial will be ongoing.
12-07-2023	4.2	• Change in reporting of SUSARs p. 35-36	The update refers to the new procedure applicable for the Central Denmark Region. This is standard procedure for the entire region.
12-07-2023	4.2	• Statistical analysis method added for "Patient experienced dyspnoea" p. 40	Due to unknown reasons the statistical method for analysing "Patient experienced dyspnoea" was missed in the original protocol.
12-07-2023	4.2	• Name of GCP monitor added p. 10	The monitor has firstly been designated now.
12-07-2023	4.2	• Sex added as stratification factor p. 21	It is the sponsors goal to ensure an equal gender distribution in all areas in this trial. This applies both to the composition of the trial steering committee, personnel employed in the trial and to the allocation of participants. We will however, not make an equal distribution of sex among the participants because it would introduce bias and we would lose external validity as it would not represent the true distribution. However, by introducing stratification on sex, we simply ensure an

			equal distribution of gender between the two groups (the intervention and the comparator arm).
12-07-2023	4.2	• Exclusion criteria in "Summary" updated p. 11	There was an error between the exclusion criteria in the summary and the rest of the protocol. It has now been corrected.
12-07-2023	4.2	• Specification of standard treatment p. 27-28	The reason for the change in the protocol, is that we realized that we would change the standard operating procedure used for treatment in the control arm if we specified the flow of oxygen used to correct a low blood oxygen saturation <88%. Several local guidelines describe treatment of patients with a low blood saturation in the prehospital setting in the Central Denmark Region. By specifying a specific flow target, a high risk of changing the current management would be introduced.
12-07-2023	4.2	• Missing objective added p. 18	There was an error between the secondary outcomes and the trial's secondary objectives. It has now been corrected.
12-07-2023	4.2	• Updated logo and project overview	N/A
12-07-2023	4.2	• New exclusion criteria	"Prior decline to consent in the trial" has been added to the list of exclusion criteria due to

			technical challenges with the automatic exclusion. Section 13.2.4 has been updated to reflect this change.
12-07-2023	4.2	• New supplier of investigational medical products	Due to new regional supplier of medical gasses the supplier and name of products has been changed. The content of the products is the same as the prior.
12-07-2023	4.2	• Appendix 6 & 7 pictures of labelling changed reflecting the new supplier	Due to new regional supplier
12-07-2023	4.2	• SmPC, in appendix 3 & 4, for investigational medical products updated to reflect the new supplier	Due to new regional supplier
17-08-2023	4.3	• Inclusion criteria added	For clarification "Need of inhaled bronchodilators" has been added as an inclusion criterion. The need of inhaled bronchodilators has all the time been the focus of the trial and a part of the description of the targeted patient population.

1 Summary

**Sponsor-Investigator:**
Martin Faurholdt Gude<br>Prehospital Emergency Medical Services (Præhospitalet)<br>Olof Palmes Allé 34 1. sal<br>8200 Aarhus N
**Title of study:**
**STOP-COPD S**tandard vs **T**argeted **O**xygen Therapy **P**rehospital for **C**hronic **O**bstructive **P**ulmonary **D**isease

**Trial Steering Committee**
Sponsor-Investigator:<br>Martin Faurholdt Gude, MD, Prehospital Emergency Medical Services (Præhospitalet),<br>Olof Palmes Allé 34 1. sal, 8200 Aarhus N<br><br>Study coordinator:<br>Arne Sylvester Rønde Jensen, Prehospital Emergency Medical Services (Præhospitalet),<br>Olof Palmes Allé 34 1. sal, 8200 Aarhus N<br>Phone: 0045 22396968<br>Mail: arjens@rm.dk<br><br>Members:<br>Ulla Væggemose, Head of research, academic coordinator, associate professor, PhD, Prehospital Emergency Medical Services (Præhospitalet), Olof Palmes Allé 34 1., 8200 Aarhus N<br><br>Søren Helbo Skaarup, MD, consultant, PhD, Afdeling for lungesygdomme og allergi. Aarhus University Hospital.<br><br>Søren Paaske Johnsen, MD, PhD, clinical professor University of Aalborg<br><br>Sophie-Charlott Seidenfaden, MD, PhD, Post Doc, Prehospital Emergency Medical Services (Præhospitalet)<br><br>Michael Drevland, Clinical Prehospital Specialist, Prehospital Emergency Medical Services (Præhospitalet)<br><br>Tina Haahr Nørgaard, Department Manager, Department of Education and Quality, Prehospital Emergency Medical Services (Præhospitalet)
**Data monitoring committee:**
• Anders Gade Kjærgaard, MD, PhD, department of anaesthesiology and intensive care Aarhus University Hospital, Denmark<br>• Mikkel Brabrand, MD, PhD, FRCP(Ed), FESEM, clinical professor (University of Southern Denmark), Consultant – Department of Emergency Medicine Odense University Hospital

• Tim Alex Lindskou, Senior researcher, PhD, Centre for Prehospital and Emergency Research, Aalborg University and Aalborg University Hospital, Denmark
**Good Clinical Practice monitoring:**
Birgitte Horst Andreasen, The GCP-unit, Aarhus and Aalborg University hospitals, Olof Palmes Allé 15,<br>8200 Aarhus N
**Study center:**
Prehospital Emergency Medical Services, Central Denmark Region
**Planned study period:**
Planning 2020-2022<br>Enrolment 2023-2025<br>End of trial: 30. September 2025<br>Reporting 2025-2026
**Patient population:**
Prehospital patients with suspected Acute Exacerbation of Chronic Obstructive Pulmonary Disease treated with inhaled bronchodilators
**Intervention:**
Titrated oxygen strategy - a mix of supplemental oxygen and compressed atmospheric air as driver for inhaled bronchodilators
**Comparator:**
Standard care using compressed oxygen (100%) as driver for inhaled bronchodilators
**Methods:**
Interventional, prospective, randomized 1:1, parallel groups, patient blinded, prehospital, single center, acute, superiority trial
**Inclusion:**
Patients over the age of 40<br>EMT or Paramedic suspected AECOPD<br>Confirmed suspicion of COPD<br>Need of inhaled bronchodilators
**Exclusion:**
Non-chronic obstructive pulmonary disease (COPD) bronchospasm<br>Known or suspected pregnancy<br>Prehospital Non-invasive, invasive or assisted bag mask ventilation<br>Allergy to inhaled bronchodilators (Salbutamol)

Transfer between hospitals
More than 2 doses (5 mg salbutamol) inhalation drug, acute treatment by EMS personnel, before allocated treatment is initiated
Readmission within 30 days from a previous randomisation
Suspicion of acute coronary syndrome
Prior decline to participate in the trial
**Sample size:**
Intervention group: 944 patients<br>Comparator group: 944 patients
**Primary Outcome:**
30-day mortality
**Secondary outcomes:**
Mortality, (24-hour)<br>Mortality, (7-day)<br>Length of hospital stay<br>ICU admission rate<br>Length of ICU stay<br>In-hospital need for NIV within 24 hours, 7 days and 30 days<br>Time to NIV<br>In-hospital need for invasive ventilation within 24 hours, 7 days and 30 days<br>Time to invasive ventilation<br>Acidosis on arrival to hospital<br>The degree of acidosis based on the pH-value<br>Patient experienced dyspnoea on a scale 0-10 (see section 8.3)<br>Readmission rate<br>Time to readmission

2 Project overview

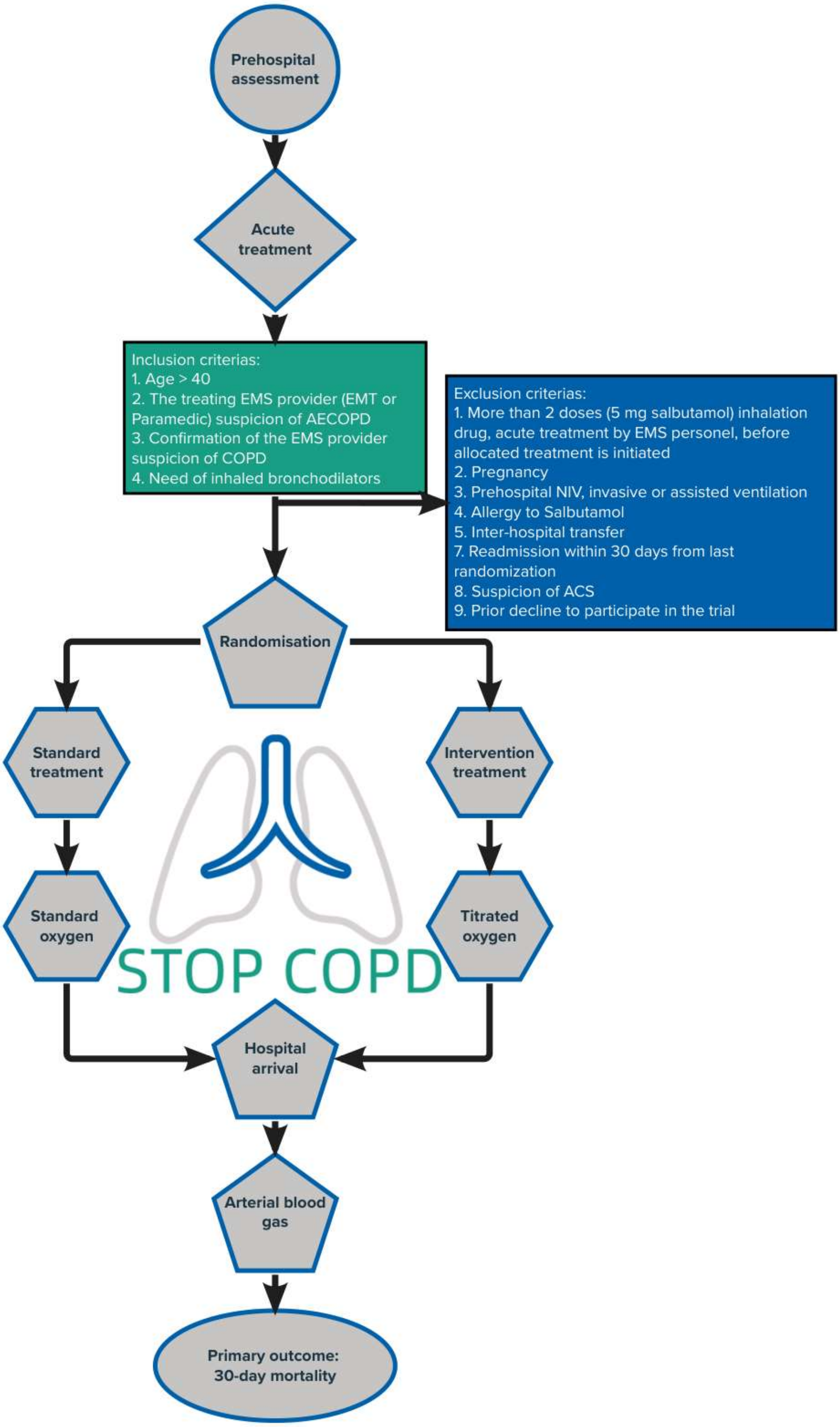

3 Background

3.1 Incidence and mortality

COPD is a widespread condition, the third leading cause of death worldwide, and affecting around 328 mill. people[1, 2]. In Denmark, estimated 200.000-400.000 people live with COPD[3, 4]. An acute exacerbation of chronic pulmonary disease (AECOPD) is frequently encountered in the prehospital setting, and the Prehospital Emergency Medical Services of the Central Denmark Region (PEMSCDR) dispatches 2000-3000 ambulances each year to patients with suspected AECOPD and need for inhaltiondrugs. AECOPD have a high in-hospital mortality varying from around 5% to 10%[5, 6]. Based on 2020 data from the Danish COPD registry the 30-day mortality in the CDR was 13% (95%CL 11-14) without significant variance between hospitals[7]. COPD patients with respiratory failure that require invasive or non-invasive ventilator (NIV) support have an even higher in-hospital mortality ranging from 11% and up to 31%[7, 8]. This emphasizes the importance of a prehospital treatment that seeks to prevent the development of respiratory failure.

3.2 Pathophysiology

In patients with COPD the pathophysiological cornerstone is an impaired ability to exhale carbon dioxide (CO_2) at a given minute ventilation reflecting an enlarged physiological dead space[9].
It has been long known that inspired high oxygen concentrations might exaggerate poor CO_2 excretion causing respiratory acidosis and subsequently a higher mortality in AECOPD [10].
The underlying mechanisms are several and the following have been described: attenuation of "hypoxic pulmonary vasoconstriction", the Haldane effect[11-13], and impaired respiratory drive.

3.2.1 Hypoxic Pulmonary Vasoconstriction

To maintain an optimal ventilation/perfusion (V/Q) ratio for pulmonary gas exchange, the pulmonary vasculature constricts in response to a decreasing alveolar O_2 concentration. This ensures a normal V/Q ratio redirecting the blood flow to lung areas with good ventilation.
COPD patients have an altered V/Q ratio because of destruction of alveolar septa resulting in large air pockets with only peripheral gas exchange (emphysema). This leads to dead space ventilation with increasing pCO_2 in the blood[9].
If the O_2 concentration increases in the poorly ventilated alveoli, as with oxygen therapy (based on supra-normal oxygen fractions with unchanged poor ventilation), the associated

capillaries will dilate and increase blood flow (inhibition of the pulmonary vasoconstriction), to maintain the normal V/Q ratio[11-13]. In the case of COPD patients, capillary blood is now led through poorly ventilated alveoli, which prevents effective excretion of CO_2, and lowers the amount of blood led through the well-ventilated alveoli, hence further lowers the excretion of CO_2. On the other hand, the inhibition of the pulmonary vasoconstriction caused by supplemental oxygen therapy lowers pulmonal vascular resistance which is beneficial for COPD patients who has COPD associated pulmonal hypertension.

3.2.2 The Haldane effect

CO_2 produced by metabolism is mainly transported in the blood from the tissue to the lung after a chemical reaction with H_2O. This process is catalysed by the enzyme carbonic anhydrase where CO_2 and H_2O react (fuses) and subsequently separates into H^+ that binds to haemoglobin (Hb) and to HCO_3^- that dissolves in plasma. In the pulmonary capillaries, the O_2 level is high and Hb begins to bind O_2 because of a higher affinity. This increases free H^+ and the high level of H^+ turn the reaction chain around where H^+ reacts with HCO_3^- to form CO_2 and H_2O. The CO_2 diffuses into the air in the alveoli (if the alveoli is well ventilated and have a normal structure) and then CO_2 gets excreted with ventilation. This is known as the Haldane effect[11-13].

This means that more CO_2 can be excreted in an oxygen-rich environment, which is found in the pulmonary capillaries, and accentuated during oxygen therapy if ventilation is increased. However, in AECOPD patients the ventilation is already near maximum capacity (still inefficient because the enlarged physiological dead space) and therefore, when the fraction of inspired oxygen (FiO_2) is increased with oxygen therapy, ventilation cannot compensate for the excess amounts of free CO_2. This, together with the consequences of the inhibited pulmonary vasoconstriction, leads to a further increase in the arterial blood measured partial pressure of carbon dioxide ($PaCO_2$) and associated acidosis.

3.2.3 Impaired respirator-drive in patients with COPD

The well-known theory describing a shift from normal CO_2-based respiratory drive to hypoxic drive in patients with severe COPD has been questioned[12, 14]. Still, an association between hyperoxia and low minute ventilation exists originating from both decreased tidal volume and respiratory frequency. The effect from hyperoxia on minute ventilation is greatest within the first 5 minutes hereafter the ventilation increases to a sub-normal level near the pre-oxygen state[11].

All the above-mentioned mechanisms resulting in increased $PaCO_2$ and acidosis both representing physiological changes worsening overall homeostasis and pulmonary arterial hypertension and in general a comorbid COPD–heart failure that are common in many patients with COPD.
In addition to the mechanisms described above increasing $PaCO_2$ and acidosis in AECOPD treated with supplemental oxygen delivery, the AECOPD condition results in an extensive increased work of breathing that eventually can lead to exhaustion, decreasing minute ventilation and worsening CO_2 retention creating a viscous spiral.

3.3 Current evidence

A Cochrane systematic review from 2020[15] found only one randomized clinical trial (RCT) on titrated oxygen for prehospital COPD patients, and concluded that "*More evidence is required to optimise the management of people with AECOPD and provide increased generalisability to the findings of this review".* To date, Austin et al. (2010) [16] conducted the only RCT on oxygen treatment of prehospital suspected AECOPD patients including 405 patients. In an intention to treat analysis, a decrease in mortality was found (9% to 4%) when changing a "high flow" oxygen protocol with a "titrated" oxygen protocol in patients with *suspected* AECOPD. The finding was statistically significant and the scale of the mortally reduction was highly clinically relevant. In a subgroup analysis only based on the *confirmed* AECOPD a significant decreasing mortality from 9% to 2% was seen. A reduction in mortality was also seen for AECOPD suspected patients with a final diagnosis of a non-COPD respiratory condition (9% to 4%).
A review on patients admitted to an emergency department (ED) with AECOPD patients with hyperoxia (PaO_2 >100 mmHg /13,3 kPa) had an odds ratio (OR) of 8.51 of having serious adverse outcome with normoxia (PaO_2 60-100 mmHg / 8,0-13,3kPa) as the referent group. In addition, the OR was 1.45 for a serious adverse outcome in the hypoxia (PaO_2 <60 mmHg / 8,0 kPa) group also compared to the reference group[17]. An observational study found that serious adverse outcomes were associated with a OR of 1.1 pr. 10 mmHg (1.33 kPa) rise in PaO_2 [18].
Among prehospital patients with a final discharge diagnose of AECOPD, a decrease in 30-day mortality (19,6% to 4,6%) was observed between periods with two different treatment protocols - a period with "high flow oxygen" and a period with "titrated oxygen", in [6].
The proportion of patients with AECOPD arriving at hospital by ambulance receiving inappropriate oxygen therapy is as high as 88.7% of whom 33.3% had respiratory acidosis[5].
The need of another prehospital RCT's to investigate the optimal oxygen therapy to AECOPD patients have been identified several times [5, 6, 15, 18].

For in-hospital AECOPD patients, Bardsley et al.[19] found that a titrated oxygen strategy during bronchodilator inhalation, resulted in a lesser increase in the amount of $PtCO_2$ in blood (subcutaneous measured) compared to standard oxygen strategy with high oxygen fractions. Unfortunately, no patient related outcomes were investigated.
Edwards et al.[20] reported similar finding in a similar study on COPD patients in stable chronical period of COPD.
Gunawarden et al. [21] only found a rise in CO_2 in admitted COPD patients in "relatively stable" condition when making a subgroup analysis of patients defined as CO_2 retainers.

3.4 Guidelines

British Thoracic Society's "Guideline for oxygen use in adults in healthcare and emergency settings"[22], the British NICE guidelines[23] and the "Thoracic Society of Australia and New Zealand oxygen guidelines for acute oxygen use in adults"[24] all recommend COPD patients to be treated with oxygen targeting SpO_2 at 88-92%. They also recommend the use of non-oxygen driven nebulizers, and if not available the use of oxygen driven nebulizers to be limited to 6 minutes. Finally; they recommend ambulance services to implement non-oxygen driven nebulizers[22]. The 2019 national guideline on emergency oxygen published by the Danish Health Authority and The Danish Society of Respiratory Medicine also recommends the targeted SpO_2 at 88-92%[25, 26]. Danish Health Authority's national clinical guideline on oxygen therapy for critically ill patients, recommends oxygen treatment in adults, without risk of hypercarbia, to be initiated if SpO2 is less than 94%. However, the specific recommendation to maintain the blood saturation above 93% is noted as a weak recommendation and is based on a single systematic review with *"serious risk of bias and low external validity"* quoted from the 2019 Danish guideline[25]. Also, the systematic review was based on 25 randomize clinical trials of which 4 regarded surgical patients, 9 regarded STROKE or traumatic brain injury (TBI) patients, 8 on post-ROSC or myocardial infarction and 4 on sepsis or ICU patients. Furthermore, 8 of the randomized studies did either not report any results or were pilot studies. Most studies did not use oxygen levels relevant to the STOP-COPD trial. In conclusion, the 2019 Danish guideline on oxygen therapy for acute patients has a low generalizability to prehospital patients in general and specifically to the hypoxic prehospital patients. Noted as a final remark "patients with a risk of hypercapnia should be treated with titrated oxygen with aiming at a saturation SpO2 88-92%" according to the Danish guideline.

3.5 Standard of care in Denmark

The PEMSCDR system delivers treatment to patients with suspected AECOPD regularly. The treatment consists primarily of inhaled nebulized bronchodilators (Salbutamol see appendix 2), where the nebulizer is driven by compressed oxygen despite the local prehospital standard operating procedure (SOP) recommending a SpO_2 target of 88-92%.
The EMT's and paramedics in Denmark work under delegation from a medical doctor, the delegating doctor. The delegating doctor develops treatment SOP's which the EMT's and paramedics adopts. In the case of COPD and Salbutamol treatment, the indication mentioned in the SOP is bronchospasm (see appendix 2). In severe cases the patients are also treated with IV steroids prehospitally.
The PRU will in some cases be dispatched simultaneous with the ambulance based on the emergency call or on request from the ambulance personnel. The PRU unit provides the option for advanced treatment: e.g., Beta-2-agonists for systemic administration; combined inhaled drug (Fenoterol and Ipratropium); and ventilator treatment (NIV or intubation-based ventilation).
In the Danish EMS, patients with AECOPD are widely (88.7%) treated with inappropriately high fractions of supplemental oxygen evident by high PaO_2 in arterial gas analysis at admission[5]. This is in line with Susanto et al.[27] who found a widespread use of supplemental oxygen despite COPD patients having SpO_2 >92%.

4 Trial objectives

The objectives of this trial are:

Primary objective: To determine whether prehospital titrated oxygen strategy in patients with suspected AECOPD will decrease 30-day mortality compared to patients receiving standard care.

Secondary objectives:

- To determine whether a prehospital titrated oxygen strategy for AECOPD patients will have a positive effect on experienced dyspnoea, rated on a scale from 0-10 compared to patients receiving standard care (see section 8.3)
- To determine whether a prehospital titrated oxygen strategy for AECOPD patients will reduce the in-hospital need for NIV or invasive ventilation compared to patients receiving standard care.

- To determine whether a prehospital titrated oxygen strategy for AECOPD patients will result in a reduced 24-hour and 7-day mortality compared to patients receiving standard care.
- To determine whether a prehospital titrated oxygen strategy for AECOPD patients will reduce the proportion of patients with respiratory acidosis ($PaCO_2$ >6,3 kPa AND pH <7,35[6]) and the degree of acidosis measured on arrival to hospital compared to patients receiving standard care.
- To determine whether a prehospital titrated oxygen strategy for AECOPD patients reduces mortality (24 hours, 7 days, 30 days), acidosis, intensive care unit (ICU) admission rate and need of assisted ventilation compared to patients receiving standard care analyzed on a subgroup level based on prehospital transport time.
- To determine whether a titrated oxygen strategy has an effect on time to intensive care admission, non-invasive ventilation or endotracheal assisted ventilation events compared with standard care
- To determine if a titrated oxygen strategy will lower the readmission rate compared with standard care
- To determine whether a prehospital titrated oxygen strategy for patients with AECOPD will result in reduced length of hospital and ICU stay compared with patients receiving standard care

5 Trial Design

5.1 Overview

The STOP-COPD trial is a patient blinded, randomized, parallel group, superiority trial investigating titrated oxygen strategy on prehospital suspected AECOPD patients compared to standard care. The trial will take place in the PEMSCDR, which has both public and private (contractual based) services. The trial will be performed as an acute trial, see section 13.

5.1.1 Study procedures

**Procedure/ Intervention**	**Description**
**Pre intervention**	
Acute treatment	All patients are treated acutely according to standard, if not in the ambulance.
Randomization	All patients eligible for inhalation treatment are screened using the TrialPartner/ REDCap randomization site

Enrolment	All patients fulfilling inclusion and exclusion criteria are enrolled
**Per-intervention**	
Intervention treatment	Targeted oxygen therapy SpO2 88-92% during treatment and transport.
Standard treatment	Standard treatment during treatment and transport
**Post intervention**	
Consent gaining	Informed consent is gained from patient or relative and legal guardian as soon as possible after hospital arrival.
Follow up	Follow up in PPR are made on first normal working day after enrolment and follow up in EPR are made on day 30 after enrolment.

5.2 Setting

The trial will take place in the Central Denmark Region EMS system. All EMS units are dispatched from the same regional emergency medical dispatch center (EMDC), which is staffed with an emergency medical doctor in daytime from 8 AM to 8 PM. From 8 PM to 8 AM, this function is outsourced to the PRU units. An emergency can be allocated one or more from a total of five levels of care: a lying transport with no treatment possibilities; an ambulance staffed with an EMT team, an ambulance staffed with a paramedic team, PRU and helicopter emergency medical service (HEMS) units. This trial will only take place in the ambulances staffed with either a EMT or a paramedic team. In some cases, one of the physician manned units (PRU or HEMS) will be dispatched together with an EMT or paramedic ambulance, in these cases the patient will be enrolled if no NIV or invasive ventilation is initiated, this last rule is believed to introduce very limited introduction of selection bias due to the very limited use of NIV prehospitally. Pre excluding patients seen by PRU will introduce significant selection bias where patients with the most severe AECOPD gets excluded. Sensitivity analysis regarding PRU involvement will be made on all outcomes See section 5.10.

5.3 Rollout

The trial will use a stepwise implementation over 4-8 weeks according to the iterative principles described by the plan-do-study-act (PDSA) circles for implementation processes[28]. The region has 6 hospitals, ranging from small local hospitals to a large university hospital, all capable of receiving COPD patients. The region's EMS system manage a total of 69 (operational) ambulance units spread throughout the region according to population density

and geography. The implementation process will happen in 4 steps and include evaluating meetings in the study group according to the PDSA model. If significant changes to the study setup are made the relevant authorities will be notified.

5.4 Outcomes

5.4.1 Primary Outcome

Outcome	Assessment
Mortality, 30-day	EPR – by study personnel

5.4.2 Secondary outcomes

Outcome	Assessment
Mortality, 24-hour	EPR – by study personnel
Mortality, 7-day	EPR – by study personnel
Length of hospitalization	EPR – by study personnel
ICU admission rate	EPR – by study personnel
Length of ICU stay	EPR – by study personnel
In-hospital need for NIV within 24 hours, 7 days and 30 days	EPR – by study personnel
Time to NIV	EPR – by study personnel
In-hospital need for invasive mechanical ventilation within 24 hours, 7 days and 30 days	EPR – by study personnel
Time to invasive ventilation	EPR – by study personnel
Proportion of patients with respiratory acidosis on arrival to hospital	EPR – by study personnel
The degree of acidosis based on the pH-value	EPR – by study personnel
Patient experienced dyspnoea on a verbal rating scale 0-10 (see section 8.3)	PPR – by study personnel
Readmission rate	EPR – by study personnel
Time to readmission	EPR – by study personnel

5.5 Allocation

Patients will be randomized in a 1:1 ratio to either titrated oxygen strategy or standard treatment.

5.6 Randomization

The patient will be randomized to standard treatment or titrated treatment according to section 7 by EMTs or paramedics using TrialPartner (an ad-on to REDCap) supported by The Clinical Trail Unit Aarhus University, Denmark. This site will be accessible by smartphone or tablet and require no log in information. A randomized block design will be utilized using receiving hospital, sex and age group (above/below 70 years of age) as blocking factors. Each block will be of random size comprising 4, 6 or 8 patients. The randomization site will need information about receiving hospital, age, CRN and inclusion/exclusion criteria – all data will be uploaded encrypted. The EMTs and paramedics participating in the STOP-COPD trial will be trained and informed about the randomization process before enrolment starts. If a patient has been included previously and withdrawn, for any reason, the TrialPartner randomization site will deem the patient not eligible for inclusion.

5.7 Blinding

The trial will be single blinded. The patients will be blinded to treatment allocation. The EMTs or paramedics will not be blinded because of practical, ethical and security problems with carrying compressed gas without known content in an ambulance. As part of study informed and training, the EMT's and paramedics will be attentive to keep all AECOPD suspected patients blinded to the treatment allocated.
Thus, a protocol for emergency unblinding is not necessary.

5.8 Study drugs

All drugs in the study are used according to the marketing authorisation. Thus, the study can be defined as a "Low-intervention clinical trial"[29].

5.8.1 Investigational medicinal products

5.8.1.1 Oxygen (Medicinsk Oxygen ”Air Liquide”)

Oxygen (Medicinsk Oxygen ”Air Liquide”) will be used in both treatment groups. Oxygen (Medicinsk Oxygen ”Air Liquide”) consists of 100% O_2 compressed in 2 or 10 l. pressure cylinders, stored in the ambulance or in the airway bag. In the standard care group oxygen will be used as the only driver for the inhaled bronchodilator. In the intervention group oxygen

will be used to titrate the SpO_2 level to 88-92% but the driver for inhaled bronchodilators will be compressed atmospheric air (see section 5.7.1.2). In both study groups the opportunity exists to escalate the oxygen levels if the patient remains hypoxic. The duration of treatment will be dictated by the patient's clinical presentation (the presence of bronchoconstriction) and can be applied if needed from patient contact until hospital admission. The use is in accordance with the SmPC.

The Oxygen (Medicinsk Oxygen ”Air Liquide”) will be labelled from the manufacturer. Oxygen (Medicinsk Oxygen ”Air Liquide”) will be produced, managed, and stored according to standard procedures for oxygen. Oxygen (Medicinsk Oxygen ”Air Liquide”) will be used in any other setting than the STOP-COPD trial according to standard procedures. Oxygen (Medicinsk Oxygen ”Air Liquide”) is labelled in accordance with European regulations and therefore, additional labelling is not necessary[30]. See appendix 3 for SmPC and appendix 7 for labelling.

5.8.1.2 Compressed air (Medicinsk Luft ”Air Liquide”)

Compressed air (Medicinsk Luft ”Air Liquide”) will be used in the intervention group only. Compressed air (Medicinsk Luft ”Air Liquide”)consists of 21% O_2 + 79% N_2 compressed in 10 L pressure cylinders, stored in the ambulance, due to logistical reasons it is not practical to carry a 2 l. cylinder of compressed air in the airway bag. In the intervention group Compressed air (Medicinsk Luft ”Air Liquide”) will be used as the only driver for the inhaled bronchodilator. Treatment can be ongoing, if indicated, from patient contact and until hospital admission. Compressed air (Medicinsk Luft ”Air Liquide”) will be labelled from the manufacturer. The use is in accordance with the SmPC.

Compressed air (Medicinsk Luft ”Air Liquide”) will be produced, managed, and stored according to the same SOP as for oxygen. When stored in ambulances the compressed air outlet and inlet will be clearly marked with "Kun til brug I STOP-COPD forsøg", see appendix 6 (ENG: Only for use in the STOP-COPD trial). Compressed air (Medicinsk Luft ”Air Liquide”) is labelled in accordance with European regulations and therefore, additional labelling is not necessary[30]. See appendix 4 for SmPC and appendix 8 for labelling.

5.8.1.3 Procedures

The investigational medicinal products, oxygen (Medicinsk Oxygen ”Air Liquide”) and compressed air (Medicinsk Luft ”Air Liquide”), will be delivered through the ordinary supplier of medical gasses to PEMSCDR (Air Liquide Danmark A/S, Høje Taastrup Vej 42, 2630 Taastrup, Denmark). The supplier will register batch numbers as usual. Expiration date will be part of the daily control of the ambulances. Because this is the usual supplier, procedures for delivery and

storage will follow the SOP for oxygen delivery in PEMSCDR. When an ambulance needs resupply of oxygen or compressed air it will follow the SOP for oxygen resupply.
The study coordinator will monitor consumption of oxygen and compressed air and make orders when needed. Registration of consumption on patient level is not possible.
At the end of the trial, the compressed air cylinders will be returned to manufacture according to standard procedure.

5.8.2 Auxiliary medicinal product

5.8.2.1 Salbutamol

Salbutamol (a pure β2 agonist) liquid for nebulization is used in all ambulances in the region as bronchodilator for treatment of bronchospasm, this includes AECOPD, asthma and allergy. The Salbutamol is used in according to the regional SOP (see appendix 2). Information about the product can be found in the investigator's brochure. The use is in accordance with the SmPC.

5.8.2.2 Berodual

Berodual (a combined β2 agonist and anticholinergic drug) liquid for nebulization is used in the region as bronchodilator for treatment of bronchospasm by the PRU units. The EMT's and paramedics does not routinely use Berodual but in case of PRU unit support Berodual might be used on the discretion of the PRU doctor. Information about the product can be found in the investigator's brochure. The use is in accordance with the SmPC.

5.8.2.3 Procedures

The auxiliary medicinal product will be delivered through the ordinary supplier of medicinal products to the ambulances of the PEMSCDR, and follow the SOPs for delivery, storage and resupply. Expiration date will be part of the daily control of the ambulances

5.9 Trial phases

The trial procedures will be divided into three phases. First the intervention phase, this will be in the prehospital setting as described in section 3.3 this phase will take around 0,5-2 hours depending on geographical location. The second phase is the consent phase, this could take up to 24 hours, due to the time of day the patients are admitted. The third phase will be follow up, here data to the CRF's are collected from prehospital patient record (PPR) system and the in-hospital electronic patient record (EPR), this phase will last 30 days because of the primary outcome, there will be no patient contact in this period. The total duration of all phases will be

around 31 days. To ensure that late registered outcomes are also discovered and registered in the eCRF, a safety follow up will be made on day 100 from inclusion.
There will be no blood draws, interventions, or additional procedures regarding this study in-hospital, meaning that in-hospital treatment will be according to treatment as usual.

5.10 Termination

5.10.1 Termination of allocated treatment

In both treatment groups the standard procedure regarding requesting PRU unit support is applicable. This means that in case of worsening, treatment failure or adverse reactions EMDC or PRU units are contacted for treatment guidance or requested for advanced treatment support. This also means that the EMDC or PRU doctor can terminate the allocated treatment and treatment on discretion by the EMDC or PRU doctor can be initiated. Termination and reason for termination of treatment will be registered and used in the data analysis.

5.10.2 Termination of the trial

Rules for termination of the trial will be available in section 10.3 and mentioned in the data monitoring committee (DMC) charter (see section 12.2).

5.10.3 Withdrawal of treatment

Due to the acute design of the trial, withdrawal of subjects from treatment is suspected to be a rare occurring event. However, if occurring all data already collected will be deleted, except in the TrialPartner randomization site, and the patient registered in the consort diagram as decline to participate under follow-up. This also applies to patients not giving consent (see section 13.2) and patients withdrawn by the EMDC or PRU (see section 5.9.1).
The sample size calculation includes a 4% lost to follow up, this makes a procedure for replacement needless (see section 10.1). A current prehospital RCT shows a withdrawal rate around 2% (*ClinicalTrials.gov identifier (NCT number):* ***NCT03481777***).

5.11 Co-enrolment

The STOP-COPD trial is the only prehospital trial investigating COPD patients in the PEMSCDR currently. If other trials end up enrolling at the same time, measures to ensure consecutive enrolment will be taken.

5.12 Medical responsibility

EMT's and paramedics work under delegation from the medical director of the PEMSCDR. The STOP-COPD trial will have no impact on this distribution of responsibility. The trial is approved by the medical director and the management of the PEMSCDR.

5.13 Recruitment

The STOP-COPD trial will be approved as an acute trial. This means that the EMT's and paramedics will be responsible for recruiting patients to the trial before patient consent is obtained. The process will be as follow; all patients in whom the treating EMT or paramedic finds indication for inhalation drug therapy will be screened for participation in the STOP-COPD trial. The screening will be using the randomization site as mentioned in section 5.5. In this process the EMT or paramedic will evaluate the patient for all inclusion and exclusion criteria. If all inclusion criteria and no exclusion criteria are fulfilled the randomization site will tell the EMT or paramedic to start intervention or standard treatment as applicable. If the patient is not included in the trial the randomization site will tell the EMT or paramedic to treat the patient as usual.

After arrival to hospital and stabilisation, consent is sought from the patient as stated in section 13.2.

5.14 End of trial

Last day of patient enrolment is the 30th of September 2025. Followed by the 30-day follow up period and the 100-day safety follow up period as mentioned in section 5.8 making the date **08/01/2026**.

6 Inclusion and exclusion

6.1 Screening and enrolment

All patients, for which the treating EMT or paramedic finds indication for inhalation treatment, will be screened for eligibility using the TrialPartner randomization site. Patients who fulfil all inclusion criteria and none of the exclusion criteria will be considered eligible for randomization.

If the patient is not declared eligible for randomization by the REDCap randomization site an electronic case report form (eCRF) is still opened in REDCap to register reason for exclusion for

later reporting according to the CONSORT guidelines. No further registration of non-included patients will be made.

Give the that the STOP-COPD trial is an acute trial, no informed consent will be sought in the prehospital setting (see section 13) given the need for prompt intervention. However, some patients may experience the treatment a little different than they are used to and other might not detect any difference. To accommodate this the following short sentence will be a part of the enrolment process and should be said right before initiation of trial treatment:

Danish: "Du får en eksperimentel behandling som, vi forventer, vil hjælpe på dit indlæggelsesforløb. Du vil under dit indlæggelsesforløb blive kontaktet og blive yderligere informeret omkring behandlingen."
English: "You will receive an experimental treatment which, we expect, will help during your hospitalization. You will be contacted during your hospitalization and be further informed about the treatment."

6.2 Inclusion criteria

- **Patients over the age of 40 years**
  35 or 40 years of age has been used in multiple other studies as a cut off to find COPD patients because of the relatively small number of COPD patients under the age of 35/40 and the relatively large group of asthma patients under the age of 35/40 [4, 16, 31-33]. The STOP-COPD trial will use an age of 40 as cut off.
- **The treating EMS provider (EMT or Paramedic) suspicion of AECOPD**
  The local SOP only states bronchospasm as an indicator for inhalation treatment see appendix 2. To fulfil this inclusion criteria in the STOP-COPD trial the treating EMT or Paramedic must also suspect AECOPD.
- **Confirmation of the EMS provider suspicion of COPD**
  Confirmation of COPD after the initial EMS provider suspicion of AECOPD can be obtained from one of the following four sources: the patient, relatives present at the scene, caretakers present at the scene, from discharge letter or text from medical records confirming COPD with the patient’s ID, medication list with COPD listed and with the patient’s ID.
- **Need of inhaled bronchodilators**

All four listed criteria must be present for inclusion in the STOP-COPD trial

6.3 Exclusion criteria

- Non-COPD bronchospasm
- Known or suspected pregnancy
- Prehospital NIV, invasive or bag mask assisted ventilation
- Allergy to inhalation drug (Salbutamol)
- Transfer between hospitals
- More than 2 doses (5 mg salbutamol) inhalation drug, acute treatment by EMS personnel, before allocated treatment is initiated
- Readmission within 30 days from last randomization
- Suspicion of acute coronary syndrome (ACS) (based on symptoms, ECG, TnT biomarker and medical consult. In concordance to the local SOP[34])
- Prior decline to participate in the trial

7 Intervention

The enrolment and randomization will start at first patient contact. If acute treatment is needed patients will receive oxygen or inhalation drugs while the randomization is being processed. After randomization the patient is transported to the ambulance where the allocated treatment is initiated. If the patient receives more than 2 doses (5 mg salbutamol) of inhalation drug, given by the EMT or paramedic (acute treatment), before allocated treatment is initiated this will exclude the patient from participating in the trial.

Rationale: Need of acute treatment of a critical patient in their home (or where the patient might be at first contact) should not be an exclusion from the trial. Start of allocated treatment outside the ambulance would not be practically possible because of the extra gas cylinder for compressed atmospheric air. Furthermore, it would be unethical to withhold treatment until the patient was in the ambulance physically.
Acute treatment is defined as urgent need for oxygen, inhalation drugs or assisted ventilation on first patient encounter, on the discretion of the treating EMT or paramedic, and only when deployed outside the ambulance.

7.1 Standard treatment

The treatment will be according to standard treatment. If the treating EMT or paramedic finds indication for inhaled bronchodilators, this will be done with Oxygen (Medicinsk Oxygen ”Air Liquide”) 6-8 l/min. as the driver for the nebulizer. The patient will have a Bi-nasal $EtCO_2$

meter placed under the nebulizer. This will measure the $EtCO_2$ during the treatment and at the same time mask the patient for group allocation. Repeated treatment will be at the discretion of the treating EMT or paramedic according to local guidelines (see appendix 2).
Following scenarios regarding SpO_2 can occur during treatment:
SpO2 <88%: Supplemental oxygen via the $EtCO_2$ -meter if needed. If the patient remains hypoxic the patient is consulted with the EMDC doctor or the PRU unit.
SpO2 88-92%: No intervention.
SpO2 >92%: No intervention.
If repeated treatment is not indicated the patient receives supplemental oxygen according to the local SOP.
At arrival to hospital the patient will have an ABG analysed within 30 minutes after handover, by hospital staff. The ABG analysis is standard in-hospital treatment and not a part of the trial.

7.2 Intervention treatment

The intervention treatment will be titrated oxygen strategy based on blood oxygenation (SpO_2). If the treating EMT or paramedic finds indications for inhaled bronchodilators, this will be done with compressed air (Medicinsk Luft ”Air Liquide”) 6-8 l/min. as the driver for the nebulizer. The patient will have a Bi-nasal $EtCO_2$ meter placed under the nebulizer. This will measure the $EtCO_2$ during the treatment and at the same time Oxygen (Medicinsk Oxygen ”Air Liquide”) can be titrated through this to a target SpO_2 of 88-92%. Repeated treatment will be at the discretion of the treating EMT or paramedic according to local guidelines (see appendix 2).
Following scenarios regarding SpO_2 can occur during treatment:
SpO2 <88%: Supplemental oxygen via the $EtCO_2$-meter up to 10 l/min, if higher oxygen levels are needed oxygen will be used as driver for the nebulizer. If the SpO_2 remains under 88% additional oxygen can be added via the $EtCO_2$-meter. If the patient remains hypoxic the patient is consulted with the EMDC doctor or the PRU unit.
SpO2 88-92%: No intervention.
SpO2 >92%: No intervention.
If repeated treatment is not indicated the patient receives oxygen to achieve SpO_2 88-92%.
At arrival to hospital the patient will have an ABG analysed within 30 min of handover by hospital staff. The ABG analysis is standard in-hospital treatment and not a part of the trial.

7.3 Clinical treatment

Patients in both treatment arms will receive the usual prehospital and in-hospital treatment, except for the prehospital intervention treatment, on the full discretion of the treating clinician.

7.4 Clinical personnel

Prior to the beginning of patient enrolment EMTs and paramedics involved in the STOP-COPD study will be informed about the trial and educated in the procedures. This includes the trial's background, objectives, the inclusion/exclusion criteria, the randomization process, the intervention treatment, and the trial procedures they are involved in. The education will consist of educational videos and e-learning materials, this will be made in collaboration with the education department of the PEMSCDR. This educational procedure has been used in multiple other prehospital studies in the region with success[35, 36]. Documentation on completed education for the EMT's and paramedics will be available in the trial master file (TMF).
Throughout the enrolment period the clinical personnel participating in the trial, other health professionals and the public will be informed through the website www.STOP-COPD.com about the study status.

8 Data collection

8.1 Process

Enrolment and randomization will be performed by the treating EMT or paramedic directly in the trial specific REDCap site via link on smartphone or tablet. The treating EMT or paramedic will register limited data in REDCap including CRN number, admission hospital and inclusion/exclusion criteria. The EMT or paramedic then presses "Randomiser" (ENG: randomize) and the specific REDCap site will tell the EMT or paramedic to which treatment arm the patient is randomized and what the treatment consists of. REDCap automatically notifies the coordinator about the enrolment of the patient by text message or e-mail. For those not randomized, a specific reason for non-inclusion/exclusion will be documented in the eCRF according to the documentation in the PPR.

To obtain patient consent, trained personnel will contact the patient at the hospital department where the patient is admitted.

If the patient is dead, has a GCS<15 or otherwise unable to give consent, then mandatory consent will be gained by a close relative (next of kin) and a physician (legal guardian) independent of investigators interests according to "REGULATION (EU) No 536/2014 OF THE EUROPEAN PARLIAMENT AND OF THE COUNCIL of 16 April 2014 on clinical trials on medicinal

products for human use, and repealing Directive 2001/20/EC" which has been active since 31th of January 2022 and the Danish "Lov om kliniske forsøg med lægemidler"[29, 37].
The PPR and EPR – systems are used to collect data for the eCRF. This is done by the study coordinator or another trained member of the project group (sponsor-investigator or study group member) after consent is gained from a patient or surrogate. The eCRF will be developed, tested and validated in REDCap before initiation of the trial to optimize the use and ensure high data quality.
Prehospital variables are collected using the ZOLL X series defibrillator (ZOLL Medical Corporation, Chelmsford, Massachusetts, USA). Data from the Zoll x series is automatically transferred to the PPR system and the data collection will happen from there.
Data collection stops 30 days after last patient (patient number 1.988) has been enrolled.

8.2 Variables

A detailed data dictionary that clearly defines all included variables in the eCRF will be created prior to patient enrolment. The data dictionary will provide the name of the variable including the code used in the database, a definition of the variable, categories for categorical variables, and units and ranges for continuous variables. All variables are collected from either PPR or EPR, the data dictionary specifies where to collect each variable.

8.2.1 Baseline characteristics

- CRN number
- Event number
- Dansk Index number
- Video call to EMDC
- Ambulance ID
- Receiving hospital
- Age
- Sex
- Known COPD
- Home oxygen supplement
- Home NIV
- Earlier AECOPD
- Current smoking
- Limited treatment level (do-not-resuscitate (DNR), no ICU, no intubation)
- Comorbidities
- Charlson Comorbidity Index (CCI)

- FEV1 % predicted (within 12 months)
- GOLD category
- Opioids
- Benzodiazepines

Known COPD is defined as the need for daily inhalation medication in the absence of asthma or COPD defined by an in-hospital diagnostic workup.

Earlier AECOPD defined as COPD related admission within 12 months from event

8.2.2 Pre-intervention characteristics

- Date and time for the enrolment
- EMT or Paramedic
- Initial contact to the EMS by general practitioner or an emergency call to the EMDC
- EMS response time
- SpO_2 on ambulance arrival
- $EtCO_2$ on ambulance arrival
- Respiratory rate on ambulance arrival
- Pulse rate on ambulance arrival
- Systolic blood pressure on ambulance arrival
- Diastolic blood pressure on ambulance arrival
- Patient-experienced dyspnoea 0-10 pre intervention (see section 8.3)
- GCS on ambulance arrival

8.2.3 Post-intervention characteristics

- Study ID
- Enrollment time
- Acute oxygen before allocated treatment
- Salbutamol before allocated treatment
- SpO_2 at hospital arrival
- $EtCO_2$ at hospital arrival
- Respiratory rate at hospital arrival
- Pulse on hospital arrival
- Systolic blood pressure on hospital arrival
- Diastolic blood pressure on hospital arrival
- Temperature in degrees Celsius
- Transport time
- Patient-experienced dyspnoea 0-10 post intervention (see section 8.3)

- GCS on hospital arrival
- Prehospital Steroids
- Prehospital I.V./I.M./S.C. Beta-2-agonist
- Inhaled Beta-2-agonist
- Inhaled anticholinergic
- Need for supplemental oxygen (besides flow for nebulizing)
- Prehospital Opioids
- Prehospital Benzodiazepines
- Hospital Opioids
- Hospital Benzodiazepines

8.2.4 Outcomes

- Dead, 30-day
- Dead, 24-hour
- Dead, 7-day
- Acidosis on hospital arrival
- Size of acidosis (pH)
- Invasive ventilation in-hospital
- NIV, in-hospital
- ICU treatment
- Length of stay at ICU
- Hospital length of stay
- Hospital discharge diagnosis
- Readmission

Patients experiencing readmission will be registered as "readmission" and only as dead if death occur within 30 days of first admission.

8.2.5 Safety

- Untreated hypoxia
- SpO_2 under 88% after allocation
- Prehospital termination of treatment

8.2.6 ABG

- PaO_2 on hospital ABG
- $PaCO_2$ on hospital ABG

- pH on hospital ABG
- HCO_3^- on hospital ABG
- Lactate on hospital ABG
- Base excess on hospital ABG

8.3 Patient-experienced dyspnoea

Patients will be asked to rate dyspnoea on a verbal numerical scale from 0 to 10. 0 being no dyspnoea and 10 being the worst imaginable dyspnoea. Patients will be asked to rate their dyspnoea two times. First time will be as soon as possible after first contact and before treatment is started. Second time will be at hospital arrival. In a recent study from the North Denmark Region, the dyspnoea score was useful for obtaining patient-reported outcomes of acute dyspnoea in the ambulance[38]. The dyspnoea scores were statistically associated with vital signs, but of limited clinical relevance. Therefore, the dyspnoea scoring in the STOP-COPD trial will be used to see whether the active treatment will change the patients experience of dyspnoea compared with standard treatment.

8.4 Data quality and validity

All clinically working personnel, EMTs and paramedics, involved in the treatment of enrolled patients will be trained to and informed about the importance of optimizing data quality and validity. This will further be optimized by having trained trial personnel entering all data from EPR and PPR to the eCRF according to the data dictionary. REDCap is designed such that data forms contain field-specific validation checks ensuring that mandatory fields are filled out and that continuous variables are within predefined ranges.
Furthermore, REDCap allows for data quality rules warning of potential incorrect data (e.g., randomization before prehospital arrival).
The eCRF will be validated thoroughly before enrolment of the first patient.
Monitoring of data quality and validity will be performed by the Good Clinical Practice (GCP) unit according to the monitoring plan. received

8.4.1 Protocol violations

Tracing of SpO_2 <88% in consecutive measurements over 5 min. without escalation of oxygen therapy in both groups. The violations will be registered in the eCRF. This data will be analysed by the DMC according to the charter, if they find systematic violations the study group will decide what measures need to be taken.

Patients with SpO2 88-92% in the both groups will be recorded for protocol violation if SpO2 >92% AND supplemental oxygen besides for nebulizing.

9 Safety

Patients with AECOPD have a high in-hospital and 30-day mortality[5, 6, 8, 39, 40]. The aim of this study is to investigate the potential benefits of applying the in-hospital guidelines (national and international) to the prehospital setting regarding levels of SpO_2 in patients with COPD[22, 25]. Patients with a SpO_2 of less than 88% is considered hypoxic and will be treated according to the protocol, no patient with hypoxia will be left untreated.
Furthermore, AECOPD patients have a high prevalence of in-hospital need of/treatment with NIV[5, 6]. The current trial will assess how a titrated oxygen strategy potentially modifies these adverse effects. The overall benefit and potential harm will be captured in our primary and secondary outcomes.

9.1 Standard treatment

The standard treatment involves giving oxygen to patients with COPD, these patients may have respiratory hypercapnia either chronic or in addition to the AECOPD. Oxygen treatment to this group of patients may constitute a risk of worsening hypercapnia and acidosis. This risk is considered acceptable because of the current local treatment guidelines that include high flow oxygen in this patient population as part of standard treatment.

9.2 Intervention treatment

The intervention treatment involves giving compressed air plus oxygen, if needed. The compressed air constitutes no risk to the patient since this is composed of the same gases as the atmosphere and as shown in appendix 4 there are no known side effects to compressed air. Oxygen in this group is titrated to the national and international recommended SpO_2 levels of 88-92%. This is expected to decrease the risk of hypercapnia and acidosis in AECOPD patients. Hypoxia therefore should not occur in this treatment group. The intervention treatment is therefore considered safe and benign to patients and expected to be beneficial as stated in section 3.

9.3 Adverse events and reactions

9.3.1 Definitions

The following definitions will be used[41, 42]:

Adverse event (AE): Any untoward medical occurrence in a patient or clinical trial subject administered a medicinal product and which does not necessarily have a causal relationship with this treatment

Adverse reaction (AR): All untoward and unintended responses to an investigational medicinal product related to any dose administered.

Serious adverse event (SAE): Any adverse event that results in death, is life-threatening, requires hospitalization or prolongation of existing hospitalization, results in persistent or significant disability or incapacity, or is a congenital anomaly or birth defect.

Serious adverse reaction (SAR): Any adverse reaction that results in death, is life-threatening, requires hospitalization or prolongation of existing hospitalization, results in persistent or significant disability or incapacity, or is a congenital anomaly or birth defect. The SARs are identified in the Danish Reference Safety Information (SmPC).

Suspected unexpected adverse reaction (SUSAR): An adverse reaction, the nature or severity of which is not consistent with the applicable product information (e.g., investigator's brochure for an unauthorised investigational product or summary of product characteristics for an authorised product).

9.3.2 Reporting

AE/ARs: EMT's, paramedics or hospital physicians report AE/ARs to the study coordinator according to the SOP. The study coordinator registers the incidents and informs the sponsor-investigator who shares the report with the sponsor. The AE/ARs are reported to the CTIS at the end of the trial by sponsor. This accounts for both investigational and auxiliary medicinal products.

SAEs: EMT's, paramedics or hospital physicians report SAEs to the study coordinator according to the SOP as soon as possible and within 24 hours. The study coordinator registers the incidents and informs the Sponsor-investigator. Sponsor-investigator assess whether SAE is related to the intervention (SAR) or not. The SAEs are reported to the CTIS database at the

end of the trial by sponsor. This accounts for both investigational and auxiliary medicinal products.

SARs: EMT's, paramedics or hospital physicians report SAEs to the study coordinator according to the SOP as soon as possible and within 24 hours. The study coordinator registers the incidents and informs the sponsor-investigator. Sponsor-investigator assess whether the SAE is related to the intervention (SAR) and whether the SAR are expected or not (SUSAR). The SARs are reported once a year to the CTIS database together with a patient safety report by sponsor. Investigational and auxiliary medicinal products will be reported in a single report.

SUSARs: Sponsor-investigator decides whether a reported SAR is unexpected and thereby classified as a SUSAR. Sponsor-investigator reports SUSAR to the GCP unit of Aarhus University within 7 days in case of fatal reactions. The GCP unit handles the further reporting of SUSARs to the EudraVigiliance database. In case of non-fatal reactions, the SUSAR is reported within 15 days.

9.4 Specific adverse reactions

The AR's and SAR's mentioned in section 9.4.1, 9.4.2 and 9.4.3 are anticipated and known reactions, furthermore they are all, except allergic reaction, symptoms on AECOPD. This correlation between adverse reactions and AECOPD symptoms makes it difficult to distinguish between the two. These reactions, will all be reported to the study coordinator and reported as mentioned in section 9.3.2 unless the treating clinician and sponsor-investigator are certain that the reaction are only related to the AECOPD. The DMC will follow the reporting of adverse reactions as stated in the charter.

9.4.1 Oxygen (Medicinsk Oxygen ”Air Liquide”)

According to the SmPC Oxygen (Medicinsk Oxygen ”Air Liquide”)has the following relevant adverse reactions: Hypoventilation, atelectasis, pleuritis, bronchopulmonary dysplasia.

9.4.2 Compressed air (Medicinsk Luft ”Air Liquide”)

According to the SmPC compressed air (Medicinsk Luft ”Air Liquide”) has no adverse reactions.

9.4.3 Salbutamol

According to SmPC for salbutamol the product has the following relevant adverse reactions: Tachycardia, palpitations, arrhythmias, headache, tremor, allergic reaction, cardiac ischemia, peripheral vasodilatation, paradox bronchospasm, agitation.

9.4.4 Berodual

According to the SmPC for Berodual the product has the following relevant adverse reactions: Allergy and anaphylactic reactions, bronchospasm, vision disturbance, tachycardia, palpitations, arrhythmias, headache, tremor, nausea and vomiting, hypertension.

9.5 Assessment of adverse events

9.5.1 Timing

In all participants, we will assess the occurrence of SARs until hospital admission using the PPR record. After hospital admission the patients will receive the usual in-hospital treatment on the full discretion of the treating physician. This in combination with the short half-life of the study drugs eliminates the need for further follow up on SARs. If the treating hospital physician suspects a SAR, it is reported to the study coordinator and sponsor-investigator according to the SOP. Patients experiencing AE, AR, SAE and SAR in the prehospital phase will have follow up 24 hours later by the study coordinator or sponsor-investigator.

9.5.2 Classification of an event

SAE/SARs will be reported to the sponsor-investigator who then classifies it as a SAE, SAR or SUSAR.

Reporting will be according to those in section 9.3.2 mentioned possibilities.

10 Sample size and statistical analysis plan

10.1 Sample size calculation

The RCT by Austin et al. [16] found an absolute risk reduction of 5% on mortality in the intention to treat analysis of suspected AECOPD. The trial used high flow oxygen in the standard treatment arm even if there was no need for inhaled bronchodilators, this will not be the case in the STOP-COPD trial. Furthermore, they used a cluster-randomized setup, making the risk of bias and confounding high. Thus, the suspected risk reduction is a conservatively

estimated to 3%. To meet this uncertainty, a sample size re-estimation is planned, according to the DMC charter, once follow-up data have been collected for the initial 500 patients (see section 12.2 and DMC charter).
To gain a power of 80% the minimum required total sample size is 1.888. The DMC can make recommendations on increasing the sample size according to the Charter for DMC.

**Study Parameters**	
Standard treatment suspected mortality	7%
Study treatment suspected mortality	4%
Significance level	0.05
Power	0.80
Drop out (expected maximum)	4%

**Sample Size**	
Standard Treatment	944
Study treatment	944
**Total**	**1.888**
Total with a power of 0.85	2.160
Total with a power of 0.90	2.526

10.2 Feasibility

Estimated enrolment rate per anno is 1.643 patients based on the following data:

10.2.1 Patients

Based on unpublished data from the PPR system on ambulance transports in the Central Denmark Region, prehospital inhalation treatment was given in 3.351 cases in 2019. Excluding patients under the age of 40 years, the number of patients was 3.135. The data do not specify the reason for inhalation treatment. Based on data from a Danish cohort study from 2019 our exclusion criteria, asthma and allergy (including angioedema) accounted for 6.12% and 6.93% respectively[43].

10.2.2 Clinician's enrolment rate

The EMT's and paramedics employed by the EMS in the CDR are familiar with prehospital trials. Based on other studies, the usual enrolment rates is around 60-70%[35].

**Variable**	**Percent**	**Number**	**Sum**
Raw data (all eligible patients)		3.351	3.351
40 years or older		3.135	3.135
Asthma	-6.12%	-192	2.943
Allergy	-6.93%	-204	2.739
Expected enrolment rate	60%	-1.096	1.643
Patients suitable for enrolment yearly			**1.643**

10.3 Stopping criteria

The DMC will recommend immediate trial stop for reasons of futility or harm based on the following criteria:

1) Patients have a statistically significantly higher risk of death in one treatment arm compared to the other (1% significance level)
2) Patients have a significantly higher risk of safety issues (see 8.2.5) in one treatment arm compared to the other (significance level on discretion of the DMC)

The DMC can also advocate for an early stop due to clear benefit or harm of a treatment, futility, slow recruitment or external evidence based on routine analyses. The sponsor-investigator will make the final decision for early stopping of the trial.

10.4 Statistical analysis plan

Baseline characteristics will be presented as median with inter quartile range (IQR) or percentages and frequencies as applicable.

10.4.1 Outcomes and statistics

Differences in the primary outcome, 30-day mortality, are calculated both as risk difference (RD) and relative risk (RR) and is performed using mixed effects Linear and Poisson regression, respectively, with robust variance estimation and random intercept on the randomization blocks. For the primary analysis the estimation is an otherwise crude analysis.

All results will be presented with 95% confidence intervals (CI). All analysis will be on an intention-to-treat basis. This meaning all patients randomized will be analysed in the allocated groups. Primary and secondary binary outcome analysis will also be presented with number-needed-to-treat or number-needed-to-harm depending on the results.

All binary secondary outcomes will be analyzed as the primary outcome. The following effect measures will be used to assess the secondary outcomes:

- Mortality (binary, within 24 hours and 7-days yes/no): RD% and RR
- Length of hospital stay (time to event, days from inclusion to discharge): Aalen-Johansen curves and Cox-regression presenting Hazard rate ratio (HR)
- Length of hospital stay (days from inclusion to discharge): mean differences using Tobit regression
- Admission at ICU (Binary, yes/no): RD% and RR
- Length of stay ICU (time to event, days from inclusion to discharge from ICU): Aalen-Johansen curves and Cox-regression presenting Hazard rate ratio (HR)
- Length of stay ICU (total number of days from inclusion to discharge from ICU): mean differences using Tobit regression
- Ventilator treatment (NIV) – also performed at non-ICU (binary, within 24 hours, 7 days and 30 days yes/no): RD% and RR
- Time to Ventilator treatment (NIV) (time to event, days from inclusion to initiation of NIV): Aalen-Johansen curves and Cox-regression presenting Hazard rate ratio (HR)
- Ventilator treatment (invasive) (binary, within 24 hours, 7 days and 30 days yes/no): RD% and RR
- Time to Ventilator treatment (invasive) (time to event, days from inclusion to initiation of NIV): Aalen-Johansen curves and Cox-regression presenting Hazard rate ratio (HR)
- Acidosis on hospital arrival (binary, acidosis yes/no): RD% and RR
- Degree of acidosis based on pH (continues): Mean/median difference
- Patient experienced dyspnea on verbal rating scale (Categorical: 0-10): Mean/median difference using linear mixed effects models
- Readmission rate from day 2 to day 30 after discharge (binary yes/no): RD% and RR
- Time to readmission from discharge up to day 30 (time to event, days from discharge to readmission, end at day 30): Aalen-Johansen curves and Cox-regression presenting Hazard rate ratio (HR)

10.4.2 Subgroup analysis

- Primary and secondary outcome for groups defined by pulse oximetry measured blood saturation (<88%, 88-92% and >92%) determined prior to first administration of inhaled bronchodilators. Analyzed as primary and secondary outcomes.
- Primary and secondary outcomes using prehospital transport time as a regression variable.
- Primary and secondary outcomes analysed on patient groups defined by a final diagnosis of AECOPD (yes/no). Analyzed as primary and secondary outcomes.
- Primary and secondary outcomes analysed on patient groups defined by NIV and invasive ventilation. Analyzed as primary and secondary outcomes.

If statistical analysis and methods not described in the protocol are deemed useful and important, in the reporting of results, this will be clearly announced in the main article.

10.5 Missing data

Patients with missing data on the outcome will be excluded in the primary and secondary analysis. Sensitivity analysis will be made using multiple imputations chained equations with 100 imputation sets and including relevant first and second order variables in the imputation model[44].
Possible differences in patient characteristics and exposure between complete cases and dropouts are addressed by sensitivity analysis adjusted by appropriate patient characteristics using inverse probability of treatment weights (IPTW). Balanced diagnostics are conducted using the threshold criteria given by Zhang et al.[45].

11 Data

11.1 Storage

Study data will be collected and managed using research electronic data capture (REDCap) tools hosted by Aarhus University[46, 47]. REDCap is a secure, web-based software platform designed to support data capture for research studies, providing 1) an intuitive interface for validated data capture; 2) audit trails for tracking data manipulation and export procedures; 3) automated export procedures for seamless data downloads to common statistical packages; and 4) procedures for data integration and interoperability with external sources. The REDCap data management system is secure and fully compliant with all regulatory guidelines and

includes a complete audit-trail for data entry validation. Through these mechanisms, as well as relevant training for all involved parties, patient confidentiality will be safeguarded.
The case report form and the consent form for each patient will be stored in REDCap during the inclusion and data handling period. After this period data will be stored in a securely electronic data base hosted by the CDR for 25 years according to EU regulations[29]. Data will be handled according to all relevant Danish and EU laws including the General Data Protection Regulation (GDPR)[48] and the Data Protection Act ("Databeskyttelsesloven")[49]. The project will be registered with the CDR's internal list of research projects.

11.2 Data access

Each patient will receive a unique trial identification number. During the trial, the sponsor-investigator, employed study personnel and coordinator will have access to the entire database except randomization. The Good Clinical Practice (GDP) unit, regulatory agencies, and other relevant entities will have direct access to source data (PPR and EPR) and to all relevant trial data including the case report form as applicable. Upon trial completion the trial sponsor-investigator, employed study personnel and coordinator will be granted access to the randomization key as well.

11.3 Data sharing

De-identified data will be made available for investigators whose proposed use of the data has been approved by local administration, 9 months after the publication and no longer accessible when data is no longer stored according to the current ICMJE recommendations and EU regulations[29, 50]. All trial-related documents will be publicly available at the trial-website www.STOP-COPD.com, patient related data will not be accessible on this website.

12 Quality and monitoring

12.1 Good Clinical Practice monitoring

The investigation site PEMSCDR will be monitored by the GCP monitoring unit from Aarhus University and Central Denmark Region. No treatment or investigations related to the trial is performed at the hospitals. Hospitals are only locations for the collection of consent. Thus, hospitals are not defined as sites in relation to GCP monitoring. A detailed monitoring plan will be developed prior to trial commencement and patient enrolment.

12.2 Data monitoring committee

The DMC will be responsible for safeguarding the interests of trial participants, assessing the safety and efficacy of the interventions during the trial, and for monitoring the overall conduct of the clinical trial. The DMC will consist of three specialists with expertise in anaesthesiology, intensive care, and clinical research, and thus covering clinical and statistical expertise as recommended[51]. The DMC will review de-identified data for safety at five predetermined milestones (200, 500, 1000 and 1500 enrolled patients), but can, at any time, require extra reviews. Unless there are group differences necessitating unblinding (as determined by the DMC), the DMC will be blinded to treatment groups. The trial will continue while the DMC reviews data. After the reviews, the DMC will create a short report to the study group with recommendations for continuation, modifications, or termination of the trial. The formal stop criteria for the trial will be those mentioned in section 10.3. The final decision on potential modifications or termination will rest with the study group and the sponsor-investigator. A detailed charter for the DMC can be will be available on the trail web page after patient inclusion starts. The DMC will Undertake interim sample size re-estimation once follow-up data have been collected for the initial 500 patients, based on the risk difference between the intervention and the control group. The DMC will then make recommendations accordingly. When making recommendations the DMC must take into account clinical relevance and feasibility.

13 Ethical Considerations

13.1 Risk/benefit assessment

13.1.1 Potential benefits

One RCT found a significant reduction in mortality when using a titrated oxygen strategy for prehospital patients with suspected AECOPD [16]. Multiple observational studies have also shown positive effects when restricting or titrating oxygen to patients suspected of AECOPD[6, 17, 18, 52]. The lack of more than one prehospital RCT’s have been confirmed by a Cochrane systematic review, and several manuscript authors. More RCTs could ensure good clinical impact and is essential to enable changes in guidelines for prehospital management of patients with suspected AECOPD.

Details about the potential benefits of the interventions are provided in the background section (see section 1). In-hospital National and international guidelines and recommendations are also listed in section 1.

13.1.2 Potential harms

For now, high flow oxygen is standard treatment, with acceptance of the known or suspected harms following high flow oxygen to this patient population. We do not believe this trial to expose patients to more harm than usual care.
The only theoretical risk of harm is untreated or undetected hypoxia. As stated in section 7 and 9 no patients are left hypoxic because the protocol includes different actions to avoid hypoxia to the extreme extend where a hypoxic patient would be treated with 100% oxygen also in the intervention arm of the study if needed.
Known potential harms for oxygen and compressed air are listed in section 9 and in appendix 3 and 4. Known potential severe adverse events that may be associated with administration of salbutamol are listed in section 9.
Potential harms are monitored continuously through the trial by the data monitoring committee (see section 12) with pre-specified stopping criteria.

13.1.3 Risk/benefit ratio

As seen in section 1 and 9.1/9.2 the intervention in this trial constitutes little or no risk to the patient. In contrast, the standard treatment is considered potentially harmful according to the limited literature on prehospital treatment and the more extensive literature on in-hospital treatment. Therefore, the risk/benefit ratio is in favour of the study.

13.2 Consent in emergency situations

In the prehospital setting obtaining informed consent from the AECOPD patient is not possible. Patients with AECOPD are most often in severe dyspnoea, and therefore desperate and anxious with the need of prompt treatment. These patients are unable to receive and understand information about the trial. AECOPD patients presents with neurological impairment in at least 11% [53]. All patients who are in need of inhalation therapy, and thereby eligibly in the study, are considered to meet these requirements and are considered incapacitated at the first contact. Despite these challenges, the need for more research is evident, as stated in section 9, to improve outcomes for patients with AECOPD.
All patients fulfilling the inclusion criteria in this study have impaired respiratory capacity with dyspnoea requiring acute treatment with inhaled bronchodilators. This means that all participants have moderate to severe pulmonary impairment precluding the possibility of an informed consent. Trying to obtain one could force the patient to consent just to receive treatment without further delay.

Studies like RESIST (*ClinicalTrials.gov identifier (NCT number):* ***NCT03481777***), TRIAGE (*ClinicalTrials.gov identifier (NCT number):* ***NCT03542188***), REFACED (*ClinicalTrials.gov identifier (NCT number):* ***NCT05076435***) and "Prehospital Transfusion Strategy in Bleeding Patients" (ClinicalTrials.gov identifier (NCT number): ***NCT04879485***) all have similar study setup as the STOP-COPD trial by including patients (not in cardiac arrest or with unconsciousness), but who are in a stage of illness, that disallows an attempt to gain informed content. The above-mentioned trials are all approved as emergency trials by the Regional Ethical Committee, hence consent is gained after enrolment and intervention.

The STOP-COPD trial will adhere to the Danish Medicinal Research Ethical Committee, the Danish Health Authority, the Danish Data Protection Agency, REGULATION (EU) No 536/2014, Declaration of Helsinki, GCP-ICH guidelines and Danish law[29, 37, 42, 48, 54-56].

13.2.1 Regulations from the European Parliament

Regulations from the European Parliament allows informed consent to be obtained after enrolment in emergency situations where the following criteria are met. The regulations are implemented in Danish law and regulations[29, 37]. Arguments for this trial are inserted under every criterion:

a) *"Due to the urgency of the situation, caused by a sudden life-threatening or other sudden serious medical condition, the subject is unable to provide prior informed consent and to receive prior information on the clinical trial."*

   Patients with AECOPD with the need of inhalation treatment are most often in severe dyspnoea hence desperate to receive prompt treatment without the ability to receive and understand information about the trial. This can be due to one or more of the following: hypoxia, hypercapnia, exhaustion, infection all leading to altered cognitive abilities. Furthermore, the definition of AECOPD is an *acute* worsening of dyspnoea and thereby automatically contains a state of distress.

b) *"There are scientific grounds to expect that participation of the subject in the clinical trial will have the potential to produce a direct clinically relevant benefit for the subject resulting in a measurable health-related improvement alleviating the suffering and/or improving the health of the subject, or in the diagnosis of its condition."*

As stated in section 3, 7 and 9 the intervention is hypothesised to lower in-hospital mortality, need for in-hospital NIV, need for invasive ventilation and shorter hospital stay. All of which must be considered as a clinically relevant benefit for the patients.

c) *"It is not possible within the therapeutic window to supply all prior information to and obtain prior informed consent from his or her legally designated representative."*

In the prehospital setting managing a desperate AECOPD patient with severe dyspnoea, it is not possible to supply the patient with information before enrolment. Because of the need for immediate treatment.

d) *"The investigator certifies that he or she is not aware of any objections to participate in the clinical trial previously expressed by the subject."*

All patients who decline to participate will be registered in the eCRF as not includible, if the patient later is sought included, the randomization site will automatically declare the patient not suitable for inclusion. The registration will be deleted as soon as the inclusion sample is met.

e) *"The clinical trial relates directly to the subject's medical condition because of which it is not possible within the therapeutic window to obtain prior informed consent from the subject or from his or her legally designated representative and to supply prior information, and the clinical trial is of such a nature that it may be conducted exclusively in emergency situations."*

The trial objectives are to investigate the effects of titrated oxygen on acute worsening of COPD (AECOPD) and thereby exclusively in the emergency situation. It is not possible to obtain consent prior to the worsened state of COPD (AECOPD) because the medical condition is the main reason for the EMS contact.

f) *"The clinical trial poses a minimal risk to, and imposes a minimal burden on, the subject in comparison with the standard treatment of the subject's condition."*

As stated in section 9.2 the potential harms for the intervention group are considered very small and the risk/benefit ratio is believed to favour the intervention. The

intervention imposes no extra burden on the patient and requires minimal/no compliance.

13.2.2 Obtaining informed consent

Informed consent is obtained as soon as possible after hospitalization. Following scenarios, in order of priority, can occur:

1. **The patient is stabilised when consent is sought**:
   The patient is informed and accepts or declines to participate.

2. **The patient is not stabilised or is stabilised but comatose or invasive ventilated and a close relative is present:** A close relative is sought to obtain consent as a surrogate together with a "legal guardian"

3. **The patient is not stabilised or is stabilised but comatose or invasive ventilated and a close relative is not present:** Consent is obtained from a legal guardian.

4. **The patient dies before consent is obtained:** A close relative is sought, within a reasonable extent, to obtain consent as a surrogate, together with a "legal guardian"[57]. If no close relative is found, consent is given only by the "legal guardian".

Information regarding the trial will be written and verbal, and contain information about the background, in/exclusion criteria, risks and benefits and the trial design. Information about data sharing will also be given. The patient or surrogate will be informed that the intervention is prehospital and no other intervention regarding the trial will be performed and that a decline of consent will have no influence on any current or future treatment. The patient or surrogate then gains or declines to give consent. Consent forms will be electronic and placed in the REDCap database together with the eCRF. The conversation will be held in a calm and quiet environment. The patient will be informed about the right to have an independent assessor present during the conversation and the right to reflection time.

In the case of a surrogate consent, the patient will be sought for informed consent as soon as possible after they regain the ability to provide consent.

13.2.2.2 Gaining consent after discharge

In the case a patient is discharged before consent is obtained, the patient is contacted by telephone and asked about the possibility to get information conversation done by video call. If the patient agrees to this, all the above-mentioned information will be given by video call, afterwards the written information will be send by e-mail or postal service. The video call will be held in a calm and quiet environment, this applies to both the patient and the person gaining consent, securing patient confidentiality. The platform used for video call is delivered by the Central Denmark Region and is approved for patient consultation (https://bestilvideo.rm.dk/video/). The patient will be informed about the right to have an independent assessor present during the conversation and the right to reflection time. Correct patient identification will be secured by CRN. The process of video call will adhere to national guidelines[58].

Signing of the consent form will be done by mailing an electronic eCRF link to the patient. This option is only chosen if the patient understands the procedure, or a relative can help and if the patient owns a tablet or smartphone.

If the patient is unable to receive and understand the information over video call or is unable to use and understand the procedure about the eCRF link, the patient is sought out at home and informed as if still admitted to hospital. This applies only if the reason is due to lack of technical skills. If the reason is due to the patient being unable to provide consent, the rules for incapacitated participants apply.

It is expected that the process of video call consent will be used in exceptional few cases.

13.2.3 Responsibilities regarding consent

The sponsor-investigator is responsible for collecting consent from patients and legal guardians. The sponsor-investigator can delegate the task of gaining consent to a qualified physician. The person who gains consent should have knowledge about the disease process of COPD, furthermore the person should know and be educated in the study protocol and legal aspects of consent. The person who gains consent will be educated in GCP-regulations using e-learning from the GCP unit, certificates will be stored in the trial master file.

A legal guardian, in context of the STOP-COPD trial, is a physician with speciality in intensive care medicine who are not involved in the STOP-COPD trial as an investigator or author and who are independent of the sponsor and investigators interests. This ensures that the legal guardian is a physician who treats AECOPD patients in their clinical routine work and has a professional insight in COPD. The legal guardian will be introduced to the study material and from here gain detailed insight to the study protocol including the in-/exclusion criteria and the

responsibilities as legal guardian. The legal guardian acts according to the interest of the research participant. The legal guardian confirms these requirements when signing the consent form.

13.2.4 Decline of consent

If a patient or surrogate decline to give consent/participate, collection of data stops at that point. Data collected up to that point is deleted and patient is registered as decline to participate in the eCRF and reported according to the consort flow diagram.
A decline to participate will have no influence on the current or following treatment of the patient.
Patient and/or relatives will be informed on mentioning a decline to participate in the trial if another EMS contact arises.

13.3 Summary of ethical considerations

AECOPD patients have a broad spectrum of severity. The group of patients with the most severe symptoms will obviously have a decreased level of consciousness based on a mix of hypoxia, hypercapnia and severe dyspnoea fulfilling the criteria listed in section 13.2.1. Even when patients suffer from less severe AECOPD, the nature of the condition with experienced dyspnoea make attempts to obtaining informed consent impossible and patients in this group thereby also fulfil the criteria listed in section 13.2.1. The intervention is, as stated in section 9.2, without any known major risks, thus making it a safe trial for enrolled patients. The intervention in the trial is simple and easy to perform, with an expected high compliance from the EMT's and paramedics. We find that the conditions for an acute study are fully satisfied since the vast majority of AECOPD patients are unsuitable for informed consent to be acutely obtaining and because the treatment has to be initiated as soon as possible to correct the patients dyspnoea, hypoxia and hypercapnia and here through the patients outcome.
Moreover, there are no reported serious risks from the intervention.
Participating in the trial is voluntary and unpaid.

13.4 Insurance

The patients participating in the STOP-COPD trial are covered by the Danish patient insurance[59].

13.5 Approval from authorities

The trial will be approved by the Danish Medicine Agency and the Medical Research Ethics Committees before initiation.

14 Funding

- "Den Landsdækkende Akutlægehelikopterordning" (eng: The Nationwide Emergency Medical Helicopter Service) has supported the project with kr. 90.478 (euro 12.000) to protocol development be providing 2 month salary to the study coordinator.
- "Simon Spies Fonden" has supported the project with kr. 15.000,-
- "Eva Merete Falck Crones Fond" has supported the project with kr. 50.000,-
- "Region Midtjyllands Strategiske Forskningsmidler" has supported the project with kr. 1.175.000,-

Further private and public organisations will be applied for funding. When additional funding is achieved, amendments will be submitted through the EU clinical trials system.

All funding is handled by the financial department at PEMSCDR. Contributors have no influence on the trial e.g., the design, conduct, results or final manuscript.

15 Timeline

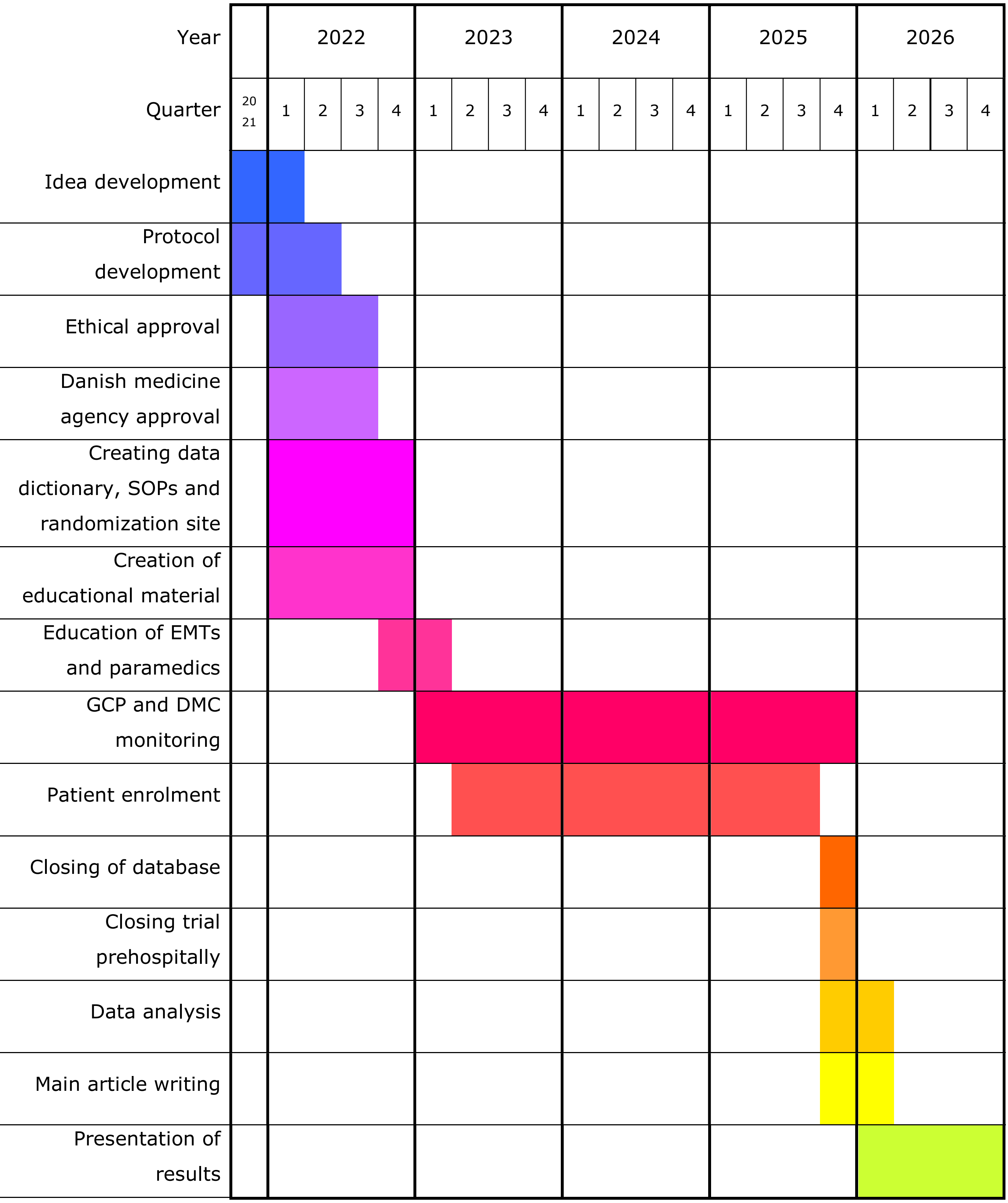
Year
2022
2023
2024
2025
2026
Quarter
20
21
1 2 3 4 1 2 3 4 1 2 3 4 1 2 3 4 1 2 3 4
Idea development
Protocol development
Ethical approval
Danish medicine agency approval
Creating data dictionary, SOPs and randomization site
Creation of educational material
Education of EMTs and paramedics
GCP and DMC monitoring
Patient enrolment
Closing of database
Closing trial prehospitally
Data analysis
Main article writing
Presentation of results

16 Publication

The study will result in two articles. First a protocol article describing the trial and the analysis plan, the article will be published when the Danish authorities have approved the trial. The second article will be the main article, this article will be published regardless of the results, both negative, inconclusive or positive results will be published following the CONSORT guidelines[60, 61]. The study coordinator will be the first and corresponding author and the sponsor-investigator the last author. Authorship will follow International Committee of Medical Journal Editors guidelines[50]. The article will be published as open access in an international peer-reviewed journal and as conference presentations.
The trial results will be shared with the participating EMT's, paramedics, patients and other interested on the study website www.stop-copd.com
Within one year from end of trial the results (according to CTR annex IV) will be uploaded to the CTIS database.

17 Division of tasks

Sponsor-Investigator: Protocol development, funding, budget, data dictionary development, Danish Medicine Agency approval, ethical approval, evaluation of SAE/SAR/SUSAR's, daily management in the absence of the coordinator, data analysis, presentation of results, trial registration, collection of consent.

Coordinator: Protocol development, funding, budget, data dictionary development, Danish Medicine Agency approval, ethical approval, trial registration, daily management, contact to GCP unit, development of educational material, data analysis, presentation of results, article writing, assessing recruitment speed, completion of eCRF.

Study group: Protocol development, data dictionary development, presentation of results.

Student assistants: Completion of eCRF.

Clinical staff: Enrolment, randomization and treatment of patients according to protocol.

DMC: See section 12.2

GCP: See section 12.1

18 References

1. Lopez-Campos, J.L., W. Tan, and J.B. Soriano, *Global burden of COPD.* Respirology, 2016. **21**(1): p. 14-23.
2. Lozano, R., et al., *Global and regional mortality from 235 causes of death for 20 age groups in 1990 and 2010: a systematic analysis for the Global Burden of Disease Study 2010.* Lancet, 2012. **380**(9859): p. 2095-128.
3. Moll, L., P. Lange, and B.H. Dahl, *KOL- sygdom, behandling og organisation*. 2011: Munksgaard Danmark. 262.
4. Jakobsen, M., et al., *Study on drug costs associated with COPD prescription medicine in Denmark.* The Clinical Respiratory Journal, 2013. **7**(4): p. 328-337.
5. Ringbaek, T.J., J. Terkelsen, and P. Lange, *Outcomes of acute exacerbations in COPD in relation to pre-hospital oxygen therapy.* Eur Clin Respir J, 2015. **2**.
6. Bentsen, L.P., et al., *A change from high-flow to titrated oxygen therapy in the prehospital setting is associated with lower mortality in COPD patients with acute exacerbations: an observational cohort study.* Acute Med, 2020. **19**(2): p. 76-82.
7. Dansk register for Kronisk Obstruktiv Lungesygdom, *Dansk register for Kronisk Obstruktiv Lungesygdom - Årsrapport for 2020*. 2021.
8. Donaldson, G.C. and J.A. Wedzicha, *COPD exacerbations .1: Epidemiology.* Thorax, 2006. **61**(2): p. 164-8.
9. Neder, J.A., et al., *Exertional ventilation/carbon dioxide output relationship in COPD: from physiological mechanisms to clinical applications.* Eur Respir Rev, 2021. **30**(161).
10. Murphy, R., P. Driscoll, and R. O'Driscoll, *Emergency oxygen therapy for the COPD patient.* Emerg Med J, 2001. **18**(5): p. 333-9.
11. Aubier, M., et al., *Effects of the administration of O2 on ventilation and blood gases in patients with chronic obstructive pulmonary disease during acute respiratory failure.* Am Rev Respir Dis, 1980. **122**(5): p. 747-54.
12. Abdo, W.F. and L.M. Heunks, *Oxygen-induced hypercapnia in COPD: myths and facts.* Crit Care, 2012. **16**(5): p. 323.
13. Hanson, C.W., 3rd, et al., *Causes of hypercarbia with oxygen therapy in patients with chronic obstructive pulmonary disease.* Crit Care Med, 1996. **24**(1): p. 23-8.
14. Savi, A., et al., *Influence of FIO2 on PaCO2 during noninvasive ventilation in patients with COPD.* Respir Care, 2014. **59**(3): p. 383-7.
15. Kopsaftis, Z., et al., *Oxygen therapy in the pre-hospital setting for acute exacerbations of chronic obstructive pulmonary disease.* Cochrane Database Syst Rev, 2020. **1**: p. CD005534.
16. Austin, M.A., et al., *Effect of high flow oxygen on mortality in chronic obstructive pulmonary disease patients in prehospital setting: randomised controlled trial.* Bmj, 2010. **341**: p. c5462.
17. Cameron, L., et al., *The risk of serious adverse outcomes associated with hypoxaemia and hyperoxaemia in acute exacerbations of COPD.* Postgrad Med J, 2012. **88**(1046): p. 684-9.
18. Wijesinghe, M., et al., *Pre-hospital oxygen therapy in acute exacerbations of chronic obstructive pulmonary disease.* Intern Med J, 2011. **41**(8): p. 618-22.
19. Bardsley, G., et al., *Oxygen versus air-driven nebulisers for exacerbations of chronic obstructive pulmonary disease: a randomised controlled trial.* BMC pulmonary medicine, 2018. **18**(1): p. 157-157.
20. Edwards, L., et al., *Randomised controlled crossover trial of the effect on PtCO2 of oxygen-driven versus air-driven nebulisers in severe chronic obstructive pulmonary disease.* Emergency Medicine Journal, 2012. **29**(11): p. 894.

21. Gunawardena, K.A., et al., *Oxygen as a driving gas for nebulisers: safe or dangerous?* Br Med J (Clin Res Ed), 1984. **288**(6413): p. 272-4.
22. O'Driscoll, B.R., et al., *BTS guideline for oxygen use in adults in healthcare and emergency settings.* Thorax, 2017. **72**(Suppl 1): p. ii1-ii90.
23. NICE, *chronic obstructive pulmonary disease in over 16s diagnosis and management.*
24. Beasley, R., et al., *Thoracic Society of Australia and New Zealand oxygen guidelines for acute oxygen use in adults: 'Swimming between the flags'.* Respirology (Carlton, Vic.), 2015. **20**(8): p. 1182-1191.
25. Sundhedsstyrelsen, *National Klinisk Retningslinje om iltbehandling til den akut syge voksne patient*. 2019.
26. Hansen, E.F., et al., *Kronisk Obstruktiv Lungesygdom (KOL) i exacerbation og Non-invasiv ventilation (NIV) The Danish Socity For Respiratory Medicine*. 2020.
27. Susanto, C. and P.S. Thomas, *Assessing the use of initial oxygen therapy in chronic obstructive pulmonary disease patients: a retrospective audit of pre-hospital and hospital emergency management.* Internal Medicine Journal, 2015. **45**(5): p. 510-516.
28. Taylor, M.J., *Systematic review of the application of the plan-do-study-act method to improve quality in healthcare.* BMJ quality & safety, 2014. **23**(4): p. 290-298.
29. *REGULATION (EU) No 536/2014 OF THE EUROPEAN PARLIAMENT AND OF THE COUNCIL.* 2014.
30. *EUROPA-PARLAMENTETS OG RÅDETS DIREKTIV 2001/83/EF af 6. november 2001 om oprettelse af en fællesskabskodeks for humanmedicinske lægemidler (EFT L 311 af 28.11.2001, s. 67.* 2001.
31. Johannesdottir, S.A., et al., *Hospitalization with acute exacerbation of chronic obstructive pulmonary disease and associated health resource utilization: A population-based Danish cohort study.* Journal of Medical Economics, 2013. **16**(7): p. 897-906.
32. Littner, M.R., *In the clinic. Chronic obstructive pulmonary disease.* Ann Intern Med, 2011. **154**(7): p. ITC4-1-ITC4-15; quiz ITC4-16.
33. Patil, S.P., et al., *In-Hospital Mortality Following Acute Exacerbations of Chronic Obstructive Pulmonary Disease.* Archives of Internal Medicine, 2003. **163**(10): p. 1180-1186.
34. *Akut Koronar Syndrom og anvendelse af Tele-EKG*, Præhospitalet, Editor. 2022.
35. Vallentin, M.F., et al., *Effect of Intravenous or Intraosseous Calcium vs Saline on Return of Spontaneous Circulation in Adults With Out-of-Hospital Cardiac Arrest: A Randomized Clinical Trial.* JAMA, 2021.
36. Behrndtz, A., et al., *TRIAGE-STROKE: Treatment strategy In Acute larGE vessel occlusion: Prioritize IV or endovascular treatment-A randomized trial.* Int J Stroke, 2020. **15**(1): p. 103-108.
37. *LOV nr 620 af 08/06/2016 Lov om kliniske forsøg med lægemidler.* 2016.
38. Lindskou, T.A., et al., *Patient experience of severe acute dyspnoea and relief during treatment in ambulances: a prospective observational study.* Scand J Trauma Resusc Emerg Med, 2020. **28**(1): p. 24.
39. Echevarria, C., et al., *Oxygen therapy and inpatient mortality in COPD exacerbation.* Emerg Med J, 2021. **38**(3): p. 170-177.
40. Lindskou, T.A., *Prehospital dyspnoea measurements*. Aalborg Universitet. Det Sundhedsvidenskabelige Fakultet. Ph.D.-Serien. 2019: Aalborg Universitetsforlag.
41. European Parlement, *Directive 2001/20/EC of the European Parliament and of the Council of 4 April 2001 on the approximation of the laws, regulations and administrative provisions of the Member States relating to the implementation of good clinical practice in the conduct of clinical trials on medicinal products for human use*. 2001.
42. European Medicines Agency, *Guideline for good clinical practice E6(R2)*. 2015.
43. Lindskou, T.A., et al., *Symptom, diagnosis and mortality among respiratory emergency medical service patients.* PloS one, 2019. **14**(2): p. e0213145-e0213145.
44. White, I.R., P. Royston, and A.M. Wood, *Multiple imputation using chained equations: Issues and guidance for practice.* Stat Med, 2011. **30**(4): p. 377-99.

45. Zhang, Z., et al., *Balance diagnostics after propensity score matching.* Ann Transl Med, 2019. **7**(1): p. 16.
46. Harris, P.A., et al., *The REDCap consortium: Building an international community of software platform partners.* Journal of Biomedical Informatics, 2019. **95**: p. 103208.
47. Harris, P.A., et al., *Research electronic data capture (REDCap)--a metadata-driven methodology and workflow process for providing translational research informatics support.* Journal of biomedical informatics, 2009. **42**(2): p. 377-381.
48. EuropeanCommission, *REGULATION (EU) 2016/679 OF THE EUROPEAN PARLIAMENT AND OF THE COUNCIL of 27 April 2016 on the protection of natural persons with regard to the processing of personal data and on the free movement of such data, and repealing Directive 95/46/EC (General Data Protection Regulation)*. 2016.
49. Folketinget, *Lov om supplerende bestemmelser til forordning om beskyttelse af fysiske personer i forbindelse med behandling af personoplysninger og om fri udveksling af sådanne oplysninger (databeskyttelsesloven)*. 2018.
50. International Committee of Medical Journal Editors, *Recommendations for the Conduct, Reporting, Editing, and Publication of Scholarly Work in Medical Journals*. 2019.
51. Tyson, J., et al., *Stopping guidelines for an effectiveness trial: What should the protocol specify?* Trials, 2016. **17**.
52. Durrington, H.J., et al., *Initial oxygen management in patients with an exacerbation of chronic obstructive pulmonary disease.* Qjm, 2005. **98**(7): p. 499-504.
53. Roche, N., et al., *Predictors of outcomes in COPD exacerbation cases presenting to the emergency department.* European Respiratory Journal, 2008. **32**(4): p. 953.
54. *World Medical Association Declaration of Helsinki: ethical principles for medical research involving human subjects.* Jama, 2013. **310**(20): p. 2191-4.
55. *BEK nr 1464 af 02/12/2016 Bekendtgørelse om information og samtykke til deltagelse i sundhedsvidenskabelige forskningsprojekter samt om anmeldelse af og tilsyn med sundhedsvidenskabelige forskningsprojekter.* 2016.
56. *LBK nr 903 af 26/08/2019 Bekendtgørelse af Sundhedsloven*. 2019.
57. *Lov om ændring af lov om kliniske forsøg med lægemidler, lov om videnskabsetisk behandling af sundhedsvidenskabelige forskningsprojekter og sundhedsdatavidenskabelige forskningsprojekter og sundhedsloven*, Sundhedsministeriet, Editor. 2022.
58. Ethics, D.N.C.f., *Guidance on decentralised clinical trials.*
59. *LBK nr 1113 af 07/11/2011 Bekendtgørelse af lov om klage- og erstatningsadgang inden for sundhedsvæsenet*. 2011.
60. Moher, D., et al., *CONSORT 2010 Explanation and Elaboration: updated guidelines for reporting parallel group randomised trials.* BMJ, 2010. **340**: p. c869.
61. Schulz, K.F., D.G. Altman, and D. Moher, *CONSORT 2010 Statement: updated guidelines for reporting parallel group randomised trials.* BMJ, 2010. **340**: p. c332.

Appendices

Appendix 1 CONSORT flow diagram

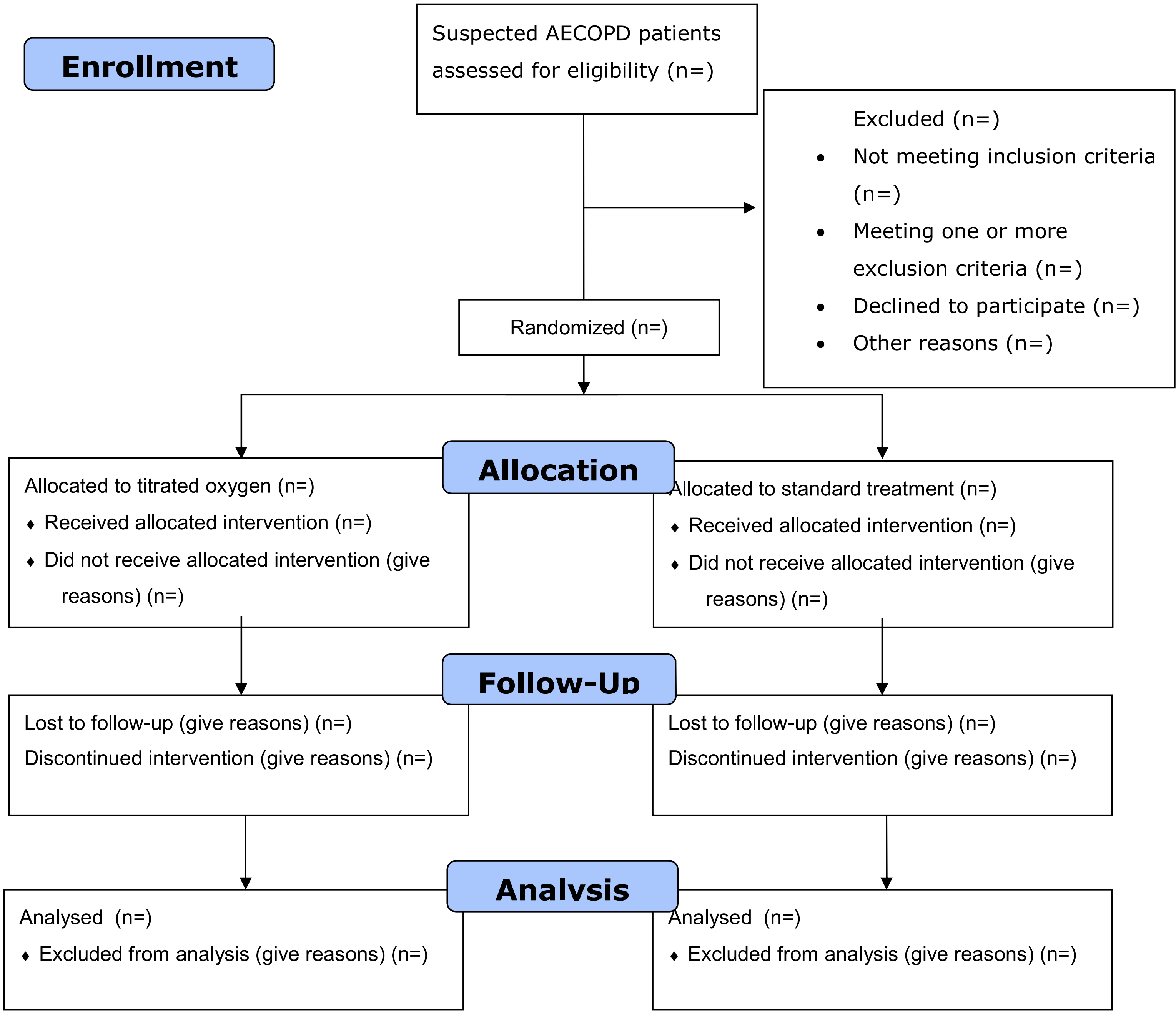

Appendix 2 SOP Salbutamol

**Salbutamol**			
Udgiver	Præhospital > Ambulancer		
Fagligt ansvarlig	Hans Kristian Sangill Brødløs/HANSBR/RegionMidtjylland	Version	8
Kvalitetsansvarlig	Marie Priergaard Løvberg/MARLOB/RegionMidtjylland	Gældende fra	07-04-2021
Ledelsesansvarlig	Palle Juelsgaard/PALJUE/RegionMidtjylland	Næste revision	06-04-2023
Ændringer	Adrenalin IV under indikation ændret til adrenalin administration. Ellers ingen ændring af instruks.		

Formål

At sikre en ensartet og korrekt anvendelse af Salbutamol inhalationsvæske.

Patientgruppe/Patientforløb/Anden målgruppe

Patientgruppe: Patienter med ekspiratorisk forlænget respirationsbesvær.

Medarbejdergruppe: Retningslinjen er gældende for ambulancebehandlere og paramedicinere.

Definition af begreber

WOB - Work Of Breathing:
Vurdering af hvor meget energi, der bruges på at trække vejret.

Fremgangsmåde

**Anvendelse**	**Indikation**	Bronkospasme.<br>Kan vælges som supplerende behandling ved tilstande med ekspiratorisk forlænget respirationsbesvær. Herunder anafylaksi, såfremt der efter Adrenalin administration fortsat er bronkospasmer.
	**Indgiftsform og -måde**	Salbutamol inhalationsvæske 1 mg/ml. i plastampul.<br>1 ampul indeholder 2,5 ml, svarende til 2,5 mg.<br>Inhalation via medicinforstøver med 6-8 L/min. iltflow.
	**Dosis og anvendelse Behandlere**	Patient > 8 år : 5 mg.<br>Kan gentages efter behov.<br>Børn 1-8 år: 2,5 mg.<br>Kan gentages én gang, til maks dosis på 5 mg.<br>kontakt læge på AMK/akutlægebil for yderligere dosering.

	**Dosis og anvendelse Paramedicinere**	Patient > 8 år : 5 mg.<br><br>Børn 1-8 år: 2,5 mg.<br><br>Kan gentages efter behov.
**Vigtigt**	**Kontraindikation**	Børn under 1 år.<br><br>Lungeødem.
	**Bivirkninger**	Tremor. Takykardi, hovedpine, arytmi.
	**Interaktioner**	ß-blokkere kan hæmme effekten af Salbutamol.
**Særligt**	**Særlige forhold**	Patienter, der behandles med Salbutamol skal monitoreres med min. SpO2, RF, WOB, BT, puls.<br><br>Opstår der indikation herfor, optages EKG-12.<br><br>Hvis hjertefrekvensen overstiger 150, skal patienten monitoreres tæt for cirkulatorisk forværring. Overvej seponering af inhalationsbehandling, hvis dette vurderes relevant.<br><br>KOL-patienter, som er iltfølsomme (5-10%), kan udvikle bevidsthedspåvirkning og åndedrætsdepression/stop, som følge af kuldioxid ophobning.<br><br>Overvej støtteventilation ved svær respiratorisk påvirkning - tilkald ALB.<br><br>**Paramediciner**<br><br>Overvej telemedicinsk konsultation med AMK/ALB mhp. administration af Solu-Medrol, hvis der ikke opnås mærkbar forbedring efter første inhalationsbehandling.
**Medikament**	**Farmakodynamik**	Korttidsvirkende β2-agonist. Virker dilaterende på bronkierne via stimulering af β2-receptorerne og den afledte afslapning af den glatte muskulatur.<br><br>Ved inhalation opnås næsten udelukkende lokal virkning i luftvejene. Virkningen indtræder efter få minutter og varer 3-6 timer.
	**Farmakokinetik**	10-20% af inhalations væsken når nedre luftveje ved inhalation (afhængig af patientens tidalvolumen).<br><br>Plasmahalveringstid 4-6 timer.<br><br>Metaboliseres i leveren.<br><br>Ca. 20% udskilles uomdannet gennem nyrerne.<br><br>Inhalation. Den systemiske absorption er ringe.<br>Dog kan en lille mængde af Salbutamolen optages systemisk, hvorved $ß_2$ receptorer i andre organer kan påvirkes og udløse bivirkninger., eks. takykardi.
	**Holdbarhed**	3 måneder, når folieemballagen til plast-ampullerne er anbrudt.

		Når folieemballagen brydes markeres på folieemballagen udløbsdato, som er 3 måneder fra anbrudsdato.<br>Hvis producentens udløbsdato er indenfor 3 måneder, når folieemballagen brydes, er det producentens udløbsdato, der gælder. Dette skal kontrolleres ved anbrud og markering af udløbsdato.<br><br>Producentens udløbsdato følges ved ubrudt folieemballage.

Dokumentation

Anvendt medicin dokumenteres i PPJ med mængde, indikation, tidspunkt og effekt.

Ansvar

Hospitalsledelsen i Præhospitalet har det overordnede ansvar for retningslinjen.

Medarbejderne har ansvaret for at anvende retningslinjen.

Referencer

- www.pro.medicin.dk
- Lægehåndbogen - KOL
- Lægehåndbogen - Astma
- Lægehåndbogen - Astma, anstrengelsesudløst
- Lægehåndbogen - Astma hos unge & voksne
- Lægehåndbogen - Astma, akut, børn
- Lægehåndbogen - Astma hos børn
- Sundhed.dk - Astma & allergi

Appendix 3 SmPC Oxygen (Medicinsk Oxygen "Air Liquide") (In Danish)

LÆGEMIDDELSTYRELSEN
DANISH MEDICINES AGENCY

18. juli 2022

PRODUKTRESUMÉ

for

Medicinsk Oxygen "Air Liquide" 100 %, medicinsk gas komprimeret

0. D.SP.NR.
25715

1. LÆGEMIDLETS NAVN
Medicinsk Oxygen "Air Liquide" 100 %

2. KVALITATIV OG KVANTITATIV SAMMENSÆTNING
Oxygen 100 % ved et tryk på 200 bar (15 °C).

3. LÆGEMIDDELFORM
Medicinsk gas, komprimeret.
Farveløst, lugtfrit og uden smag.

4. KLINISKE OPLYSNINGER

4.1 Terapeutiske indikationer
Oxygenbehandling
- Til behandling eller forebyggelse af akut og kronisk hypoksi uanset årsag.
- Som en del af friskgasflowet ved anæstesi eller intensiv behandling.
- Som drivgas i behandlingen med nebulisator.
- Til behandling af et akut anfald af klyngehovedpine.

Hyperbar oxygenbehandling
Til behandling af dykkersyge, luft-/gasemboli af andre årsager og kulilteforgiftning. Behandling af patienter, der har været udsat for kulilte, er specielt indiceret hos gravide patienter eller patienter, som er eller har været bevidstløse, eller som har udvist neurologiske symptomer og/eller kardiovaskulære effekter eller alvorlige acidoser uanset den målte COHb-værdi.

Som tillægsbehandling ved:
- alvorlig osteoradionekrose, clostridium myonekrose (gasgangræn).

4.2 Dosering og indgivelsesmåde

Dosering

Oxygenbehandling

Formålet med behandlingen er at sikre, at oxygenpartialtrykket i arterieblod (PaO_2) ikke kommer under 8,0 kPa (60 mmHg), eller at oxygenmætningen af hæmoglobin i arterieblod ikke kommer under 90 %. Dette sker ved en justering af oxygenfraktionen i den inhalerede luft (FiO_2).

Dosis (FiO_2) skal justeres i henhold til den enkelte patients individuelle behov, idet der tages hensyn til risikoen for oxygentoksicitet. Det anbefales generelt at anvende den lavest mulige dosis (FiO_2), der er nødvendig for at opnå det ønskede behandlingsresultat. I tilfælde af udtalt hypoksi kan oxygenfraktioner, der kan medføre risiko for oxygentoksicitet, indiceres (se pkt. 4.9).

Behandlingen skal evalueres løbende, og virkningen måles ved hjælp af PaO_2 eller arteriel oxygenmætning (SpO_2).

Ved korttidsbehandling med oxygen skal oxygenkoncentrationen – fraktionen i den inhalerede gasblanding (FiO_2) (undgå > 0,6 = 60 % O_2 i den inhalerede gasblanding) – bevares, således at man med eller uden positivt slutekspiratorisk tryk (PEEP) eller kontinuerligt positivt luftvejstryk (CPAP) kan opnå et arterielt oxygentryk (PaO_2) > 8 kPa.

Korttidsbehandling med oxygen skal monitoreres/følges ved hjælp af gentagne målinger af det arterielle oxygentryk (PaO_2) eller pulsoximetri, som giver en numerisk værdi for hæmoglobinoxygenmætning (SpO_2). Disse er dog kun indirekte målinger af oxygenmætningen i væv. Behandlingens effekt skal også evalueres klinisk.

I en nød-/akut situation er den normale dosis for voksne til behandling eller forebyggelse af *akut oxygenmangel* 3-4 liter i minuttet ved anvendelse af næsekateter og 5-15 liter i minuttet med maske.

Ved langtidsbehandling styres behovet for ekstra oxygen af resultatet af målingerne af gas i arterieblodet. Til justering af oxygenbehandling hos patienter med hyperkapni skal blodgasser monitoreres for at undgå en markant stigning i tensionen af kuldioxid i arterieblod.

Hvis oxygen blandes med andre gasser, må koncentrationen af oxygen i den inhalerede gasblanding (FiO_2) ikke være lavere end 21 % og må være op til 100 %.

Ved behandling af klyngehovedpine leveres oxygen via en ansigtsmaske i et ikke-genåndingssystem. Oxygenbehandling skal påbegyndes hurtigt efter anfaldets start og skal vare i omkring 15 minutter, eller indtil smerten er forsvundet. Sædvanligvis er et flow på 7-10 l/min nok, men et flow på op til 15 l/min kan være nødvendigt hos nogle patienter for at få effekt. Oxygenbehandlingen skal afbrydes, hvis der ikke opstår nogen effekt efter 15-20 minutter.

Hyperbar oxygenbehandling

Hyperbar oxygenbehandling (HBO) indebærer tilførsel af 100 % oxygen ved et tryk på over 1,4 gange det atmosfæriske tryk ved havets overflade (1 atmosfære = 101,3 kPa = 760

mmHg). Af sikkerhedsårsager må trykket ved HBO ikke overskride 3 atmosfære. Varigheden af en behandling med HBO ved et tryk, der svarer til 2-3 atm, er sædvanligvis mellem 60 minutter og 4-6 timer afhængigt af indikationen. Behandlinger kan gentages 2-3 gange om dagen om nødvendigt, afhængigt af indikationen og den kliniske tilstand. Gentagne behandlinger er oftest nødvendige i forbindelse med behandlingen af bløddelsinfektioner og iskæmiske sår, der ikke reagerer over for konventionel behandling. HBO skal gives af kompetent personale. Øgning og reducering af trykket skal ske langsomt for at undgå risikoen for trykskade (barotraume).

Pædiatrisk population
Nyfødte bør monitoreres omhyggeligt under behandling. De laveste effektive koncentrationer bør tilstræbes for at sikre passende iltning.

Administration
Oxygenbehandling
Oxygen tilføres via indåndingsluften.

Oxygen kan også tilføres via en såkaldt "oxygenator" direkte til blodet i tilfælde af bl.a. hjertekirurgi med en hjerte-lungemaskine og andre tilstande, der kræver ekstrakorporal cirkulation.

Oxygen tilføres ved hjælp af udstyr, der er beregnet til dette formål. Med dette udstyr tilføres oxygenet til indåndingsluften, og ved udånding passerer den udåndede gas med eventuel overskydende oxygen fra patienten og blandes med den omgivende luft (ikke-genindåndingssystem). Til behandlingen af klyngehovedpine leveres oxygen via en ansigtsmaske i et ikke-genindåndingssystem.
Ved anæstesi anvendes ofte specialudstyr, hvori den udåndede gas recirkulerer og delvist genindåndes (cirkulært system med genindånding).
Der findes et stort antal apparater, der er beregnet til oxygentilførsel.

Low-flow system
Det enkleste system, som blander oxygen med den inhalerede luft, f.eks. et system, hvor oxygen doseres via et simpelt rotameter og et næsekateter eller en ansigtsmaske.

High-flow system
Et system, der er beregnet til at levere en gasblanding, der svarer til patientens åndedræt. Dette system er beregnet til at levere en fast oxygenkoncentration, der ikke påvirkes eller fortyndes af den omgivende luft, f.eks. en Venturimaske med et konstant oxygenflow for at levere en fast oxygenkoncentration i den inhalerede luft.

Hyperbar oxygenbehandling
Hyperbar oxygenbehandling (HBO) gives i specielt konstruerede trykkamre, der er beregnet til hyperbar oxygenbehandling, hvor tryk på op til det, der svarer til 3 atmosfære (atm), kan opretholdes. HBO kan også gives via en meget tætsluttende ansigtsmaske, en hætte, der slutter til omkring hovedet, eller via et trakealrør.

For instruktioner om håndtering af lægemidlet før adminsitration, se pkt. 6.6.

4.3 Kontraindikationer
Normobar oxygenbehandling:
Ingen

Hyperbar oxygenbehandling (HBO):
Udrænet/ubehandlet pneumothorax (se afsnit 4.4)

4.4 Særlige advarsler og forsigtighedsregler vedrørende brugen

Høje oxygenkoncentrationer skal gives i den kortest mulige periode i forhold til at opnå det ønskede resultat, og skal monitoreres med gentagne kontroller af arterielle gastryk af oxygen i blod (PaO_2) eller hæmoglobin- oxygenmætning (SpO_2) samt klinisk vurdering.

Patienter med risiko for hyperkapnisk respirationssvigt:
Der skal udvises særlig forsigtighed ved patienter, som enten har nedsat følsomhed overfor kuldioxidspændingen i arterielt blod eller hvor der er risiko for hyperkapnisk respirationssvigt (*hypoxic drive*) (f.eks. patienter med kronisk obstruktiv lungesygdom (KOL), cystisk fibrose, morbid fedme, misdannelse i brystvæggen, neuromuskulære sygdomme, overdosis med respiratorisk depressive lægemidler.) Administration af supplerende oxygen kan resultere i respiratorisk depression og en stigning i $PaCO_2$ med efterfølgende symptomatisk respiratorisk acidose (se afsnit 4.8). Hos sådanne patienter skal oxygenbehandlingen omhyggeligt titreres. Den oxygenmætning, der skal nås, kan være lavere end hos andre patienter, og oxygen bør administreres med en lav flowhastighed.

Særlige forholdsregler for patienter med bleomycin-inducerede lungeskader: Den pulmonale toksicitet ved oxygenbehandling med høje doser kan forværre lungeskader, også selvom behandlingen indgives flere år efter den oprindelige bleomycin-inducerede lungeskade, og den oxygenmætning, der skal nås, kan være lavere end hos andre patienter (se afsnit 4.5).

Pædiatrisk population:
På grund af af den højere sensitivitet overfor supplerende oxygen bør man søge den laveste mulige effektive koncentration til opnåelse af tilstrækkelig og egnet iltning hos neonatale (se afsnit 4.2).

Hos præmature og neonatale spædbørn kan øget PaO_2 føre til præmatur retinopati (se afsnit 4.8). Det anbefales, at man starter genoplivning af neonatale, der er født til tiden eller lidt for tidligt, med luft i stedet for 100 % ilt. Til præmature børn kendes den optimale koncentration af oxygen ikke fuldt ud. Hvis det ikke kan undgås, skal supplerende oxygen omhyggeligt monitoreres og afvikles i overensstemmelse med pulsoximetri.

Hyperbar oxygenbehandling (HBO):
Hyperbar oxygenbehandling bør kun administreres af kvalificeret personale og på specialafdelinger, hvor man er opmærksom på og har udstyret til at sikre de relevante sikkerhedsforanstaltninger i relation til hyperbar brug.
Trykket bør øges og sænkes langsomt for at undgå risikoen for trykskader (barotraume).

Angst for indespærring og klaustrofobi kan forekomme under HBO-sessionen i tanken. Risk/benefit-forholdet for HBO bør omhyggeligt evalueres hos patienter med klaustrofobi, svær angst og psykose.

Respirationslidelser:
Som følge af dekompressionen stiger gasmængden ved afslutningen af den hyperbar session, samtidig med at trykket i tanken falder, hvilket kan resultere i delvis pneumothorax eller forværring af en underliggende pneumothorax. Hos patienter med en udrænet pneumothorax kan dekompression potentielt føre til udvikling af en spændingspneumothorax. I tilfælde af

pneumothorax skal pleural kavitet drænes inden sessionen, og det kan være nødvendigt at fortsætte dræningen under HBO-sessionen (se afsnit 4.3).
Herudover bør risk/benefit-forholdet for HBO grundigt evalueres hos patienter med utilstrækkeligt kontrolleret astma, lungeemfysem, kronisk obstruktiv lungesygdom (KOL) og nylig thorakal operation som følge af risikoen for gasudvidelse under HBO's dekompressionsfasen.

Diabetikere: Der har været indberettet fald i blodglukose under HBO-sessioner. Det betyder, at det kan være relevant at monitorere indholdet af glukose i blodet inden en HBO-session hos diabetikere.

Koronarsygdom: Risk/benefit-forholdet for HBO bør grundigt evalueres hos patienter med koronarsygdom. Hos patienter med akut koronart syndrom eller akut myokardieinfarkt, som også har brug for HBO, f.eks. i tilfælde af kulmonooxidforgiftning, bør HBO anvendes med forsigtighed som følge af muligheden for vasokonstriktion ved hyperoxi i koronar cirkulationen.

Øre-, næse- og halslidelser: I relation til HBO's kompressions- og dekompressionsfase er omhyggelig og grundig evaluering af risk/benefit-forholdet for HBO påkrævet for patienter med sinusitis, otitis, kronisk rhinitis, laryngocele, mastoid kavitet, vestibulært syndrom, høretab og nylig operation i mellemøret.

I relation til HBO-induceret hyperoxi bør risk/benefit-forholdet for HBO grundigt evalueres hos patienter med:
- Krampeanfald i anamnesen, epilepsi
- Ukontrolleret høj feber

Brandfare:
Oxygen er et brandnærende produkt, der fremmer forbrænding. Ved anvendelse af oxygen skal den øgede risiko for antændelse af en brand inddrages i overvejelserne:
- Risiko for brand i hjemmet: Patienter og plejepersonale skal endvidere advares om risikoen for brand ved forekomst af andre antændelseskilder (rygning, åben ild, gnister, madlavning, ovne, osv.) og/eller meget letantændelige materialer, især fedtholdige stoffer (olie, fedt, cremer, salver, smøremidler, osv.). Anvend altid vandbaserede produkter til hænder og ansigt samt næsens inderside ved samtidig brug af oxygen.
- Risiko for brand i medicinske omgivelser: Denne risiko er forhøjet i forbindelse med procedurer, der omfatter diatermi, defibrillation og behandling med elektrokonvertering.
- Brand kan opstå i ventilåbningen (opvarmning via friktion).

Der har været fortilfælde af brandsår, der skyldes utilsigtet brand ved forekomst af oxygen.

Håndtering af gasbeholdere:
Plejepersonalet og andre, som håndterer gasbeholdere med oxygen til medicinsk brug, bør være vidende om behovet for at håndtere gasbeholderne forsigtigt for at undgå beskadigelse af udstyret, især ventilen. Beskadiget udstyr kan resultere i obstruktion af udgangen og/eller visning af forkerte oplysninger på manometeret med hensyn til mængden af tilbageværende ilt i flasken samt levering af flow, hvilket kan føre til utilstrækkelig eller manglende indgift af ilt.

4.5 Interaktion med andre lægemidler og andre former for interaktion
Inhalation af høje oxygenkoncentrationer kan forværre pulmonal toksicitet, der skyldes

lægemidler såsom bleomycin (også selvom oxygen indgives flere år efter den oprindelige bleomycin-inducerede lungeskade), amiodaron og nitrofurantoin samt paraquat-forgiftning. Supplerende oxygen bør undgås, medmindre patienten er hypoxisk.

Nitrogenoxid oxideres hurtigt ved forekomst af oxygen og danner ekstremt nitrat-derivativer, som er lokalirriterende for det bronkiale epitel og den alveolo-kapillære membran. Den primære forbindelse, der dannes, er nitrogendioxid (NO_2). Oxidationssraten er proportional med den oprindelige koncentration af nitrogenoxid og oxygen i den inhalerede luft samt varigheden af kontakten mellem NO og O_2.

Der er risiko for brand ved forekomst af andre former for antændelseskilder (rygning, åben ild, gnister, ovne, osv.) og/eller meget letantændelige materialer (olier, fedt, cremer, salver, smøremidler, osv.) (se afsnit 4.4).

4.6 Graviditet og amning

Graviditet:
I dyreforsøg er der observeret toksicitet i forhold til reproduktion efter administration af oxygen ved forhøjet tryk eller i høje koncentrationer (se afsnit 5.3). I hvilket omfang disse resultater er relevante for mennesker er ikke kendt.

Normobar oxygenbehandling:
Oxygen må kun anvendes under graviditet, hvis det er nødvendigt, dvs. på vitale indikationer, hos kvinder med kritisk sygdom eller hypoxemi.

Hyperbar oxygenbehandling (HBO):
Mængden af dokumenteret erfaring med brug af HBO hos gravide kvinder er begrænset, men der har vist sig at være en fordel for fosteret ved HBO, såfremt kvinden lider af CO-forgiftning. I andre situationer bør HBO anvendes med forsigtighed hos gravide, da indvirkningen på fosteret af en potentiel stigning i oxidativ belastning som følge af for meget oxygen er ukendt. Brug af HBO bør derfor vurderes for den enkelte patient, men kan tillades under graviditet, hvis vitale indikationer taler herfor.

Amning:
Oxygenbehandling kan anvendes under amning uden risiko for barnet.

4.7 Virkninger på evnen til at føre motorkøretøj eller betjene maskiner
Ikke mærkning.

Normobar oxygenbehandling:
Oxygen har ingen indvirkning på evnen til at føre motorkøretøj eller betjene maskiner.

Hyperbar oxygenbeandling (HBO):
Der er indberettet syns- og høreforstyrrelser, som kan påvirke evnen til at føre motorkøretøj og betjene maskiner, efter HBO (se afsnit 4.8).

4.8 Bivirkninger
Forskellige former for væv udviser forskellig sensitivitet overfor hyperoxi, men det mest sensitive er lunger, hjerne og øjne.

Beskrivelse af udvalgte bivirkninger:

Respiratoriske bivirkninger

- Ved et omgivende tryk kan de første tegn (tracheobronchitis, intrathorakale smerter og tør hoste) vise sig efter 4 timer ved 95% oxygen. Der kan opstå en reduceret tvungen ventilation efter 8-12 timers eksponering for 100 % oxygen, men svære skader kræver meget længere eksponering. Interstitielt ødem kan forekomme efter 18 timers eksponering for 100 % oxygen og kan resultere i pulmonal fibrose. Indberettede respiratoriske bivirkninger som følge af HBO er generelt de samme som dem, der ses ved normobar oxygenbehandling, men tiden til indtræden af symptom er kortere.

- Nitrogenkoncentrationen/-trykket reduceres med høje oxygenkoncentrationer i inspirationsluften/-gassen. Det betyder, at koncentration af nitrogen i væv og lunger (alveolerne) falder. Alveolerne kan kollapse (udvikling af atelectase), hvis der optages oxygen fra alveolerne til blodet hurtigere, end det leveres i fraktionen af inspirationsgas). Udviklingen af atelektatiske dele i lungerne giver risiko for dårligere iltmætning af arterielt blod, på trods af god perfusion, som følge af mangel på gasudveksling i de atelektatiske dele af lungerne. Ventilations-/perfusionsforholdet forværres, og fører ultimativt til intrapulmonal shunt.

- Der kan være en ændring af modaliteterne for ventilationskontrol hos patienter med langvarige sygdomme, som forbindes med kronisk hypoxi og hyperkapni. Under sådanne omstændigheder kan indgift af oxygen i en koncentration, der er for høj, føre til respiratorisk depression, hvilket inducerer forværret hyperkapni, respiratorisk acidose og ultimativt respirationsstop (se afsnit 4.4).

Toksicitet i centralnervesystemet:
- Der kan observeres toksicitet i centralnervesystemet i HBO-situationer. Patienten kan udvikle toksicitet i centralnervesystemet, hvis denne indånder 100 % oxygen ved tryk på over 2 ATA. Tidlige manifestationer omfatter sløret syn, reduceret perifert syn, tinnitus, åndedrætsforstyrrelser, lokaliserede muskelkontraktioner især i øjne, mund og på pande. Fortsat eksponering kan føre til vertigo og kvalme, efterfulgt af ændret adfærd (angst, forvirring, irritabilitet) og til sidst krampeanfald. De hyperoxi-inducerede udledninger menes at være reversible, medfører ikke tilbageblivende neurologiske skader og går væk, når det inspirerede partielle oxygentryk reduceres.

Toksicitet i øjne:
Der er indberettet progressiv myopi i tilfælde af gentagne hyperbariske behandlinger. Mekanismen er stadig uklar, men en stigning i linsens brydningsindeks har været diskuteret. De fleste tilfælde var spontant reversible. Risikoen for irreversibilitet øges dog efter 100 behandlinger. Efter seponering af HBO skete der normalt hurtigt ophævelse af myopi i de første par uger, hvorefter udviklingen fortsætter mere langsomt i en periode, der varer fra flere uger helt op til et år. Tærsklen for antallet af HBO-sessioner, perioder eller varighed kan ikke anslås. Den er sat til at spænde fra 8 til mere end 150 sessioner.
- Præmatur retinopati: Se nedenfor.

Pædiatrisk population
Hos præmature, som har fået høje koncentrationer af oxygen, kan der opstå præmatur retinopati (retrolental fibropati).

Brandfare: Risikoen for brand er forhøjet ved forekomst af høje koncentrationer af oxygen og antændelseskilder, hvilket potentielt kan resultere i brandsår (se afsnit 4.4).

Bivirkninger med relation til HBO-proceduren:

- Uønskede virkninger af HBO er barotraumer eller konsekvenserne af flere og hurtige kompressioner/dekompressioner. De fleste af dem er ikke specifikke for brugen af oxygen og kan forekomme hos patienter, som får oxygen, samt hos det personale, der er til stede under hyperbar behandling med omgivelsesluft. Der er tale om barotraumer i ører, bihuler og hals, pulmonale barotraumer og andre barotraumer (tænder, osv.).

- Som følge af nogle trykkamres ringe størrelse kan patienten udvikle angst for indespærring, hvilket ikke er en direkte virkning af oxygen.

Bivirkninger med relation til oxygenbehandling:

<table>
<tr><td></td><td>Meget almindelige (> 1/10)</td><td colspan="2">Almindelige (≥1/100 til <1/10)</td><td colspan="2">Ikke almindelig(≥1/1.000 til <1/100)</td><td>Sjældne (≥1/10.000 til <1/1.000)</td><td>Meget sjældne (<1/10.000)</td><td>Ikke fastlagt hyppighed</td></tr>
<tr><td colspan="2">Luftveje, thorax og mediastinum</td><td></td><td></td><td>Atelectase</td><td></td><td></td><td colspan="2">Pulmonal toksicitet:
• Tracheobronchitis (intrathorakale smerter, tør hoste)
• Interstitielt ødem
• Pulmonal fibrose

Forværring af hyperkapni hos patienter med kronisk hypoxi/hyperkapni, der behandles med for meget forhøjet FiO_2:
• Hypoventilation
• Respiratorisk acidose
• Respirationsstop</td></tr>
<tr><td colspan="2">Øjne</td><td>Præmatur retinopati</td><td></td><td></td><td></td><td></td><td colspan="2"></td></tr>
<tr><td colspan="2">Almene symptomer og reaktioner på administrationsstedet</td><td></td><td></td><td></td><td></td><td></td><td colspan="2">Slimhindetørhed
Lokal irritation og inflammation af slimhinden</td></tr>
</table>

Bivirkninger, der er specifikke for hyperbar oxygenbehandling:

	Meget almindelige (> 1/10)	Almindelige (≥1/100 til <1/10)	Ikke almindelige (≥1/1.000 til <1/100)	Sjældne (≥1/10.000 til <1/1.000)	Meget sjældne (<1/10.000)	Ikke fastlagt hyppighed
**Luftveje, thorax og mediastinum**				Dyspnø		Respiratoriske forstyrrelser

	Meget almindelige (> 1/10)	Almindelige (≥1/100 til <1/10)	Ikke almindelige (≥1/1.000 til <1/100)	Sjældne (≥1/10.000 til <1/1.000)	Meget sjældne (<1/10.000)	Ikke fastlagt hyppighed
**Nervesystemet**		Krampe anfald				
**Knogler, led, muskler og bindevæv**						Lokaliseret muskelspænding
**Øre og labyrint**	Øresmerter		Trommehinde-ruptur			Vertigo<br>Nedsat hørelse<br>Akut serøs otitis media<br>Tinnitus
**Mavetarmkanalen**						Kvalme
**Psykiatriske lidelser**						Unormal adfærd
**Øjne**	Progressiv myopi					Nedsat perifert syn<br>Sløret syn<br>Katarakt*
**Tilskadekomst, forgiftning og proceduremæssige komplikationer**	Barotraume (bihuler, øre, lunge, tænder, osv.)					
**Stofskifte og ernæring**				Hypoglykæmi hos diabetikere		

* Der er indberettet udvikling af katarakt hos patienter, der gennemgik længere forløb og/eller gentagne HBO-sessioner (> 150 sessioner). Der har været observeret nogle tilfælde af novo/ny katarakt.

Indberetning af formodede bivirkninger
Når lægemidlet er godkendt, er indberetning af formodede bivirkninger vigtig. Det muliggør løbende overvågning af benefit/risk-forholdet for lægemidlet. Sundhedspersoner anmodes om at indberette alle formodede bivirkninger via:

Lægemiddelstyrelsen
Axel Heides Gade 1
DK-2300 København S
Websted: www.meldenbivirkning.dk

4.9 Overdosering

Symptomer på oxygenforgiftning er de symptomer, der ses ved hyperoxi.
Symptomerne på respiratorisk toksicitet svinger fra tracheobronchitis (intrathorakale smerter, tør hoste) til interstitielt ødem og pulmonal fibrose.
Symptomerne på toksicitet i centralnervesystemet, som er observeret i forbindelse med HBO, omfatter tinnitus, respiratoriske forstyrrelser, lokaliseret muskeltrækninger, især i

øjne, mund og på pande. Fortsat eksponering kan føre til vertigo og kvalme, efterfulgt af ændret adfærd (angst, forvirring, irritabilitet) og til sidst krampeanfald.
Toksicitet i øjnene omfatter sløret syn og nedsat perifert syn i HBO-sammenhæng.

Pædiatrisk population:
Toksicitet i øjnene hos præmature børn: Der kan forekomme præmatur retinopati hos for tidligt fødte, som har fået høje koncentrationer af oxygen.

Patienter med risiko for hyperkapnisk respirationssvigt:
Indgift af supplerende oxygen kan resultere i respiratorisk depression og en stigning i $PaCO_2$ med efterfølgende symptomatisk respiratorisk acidose.

I tilfælde af oxygenforgiftning, som er knyttet til hyperoxi, bør iltbehandlingen reduceres eller om muligt seponeres, og symptomatisk behandling opstartes.

4.10 Udlevering
GH

5. FARMAKOLOGISKE EGENSKABER

5.0 Terapeutisk klassifikation
Farmakoterapeutisk klassifikation: Alle andre terapeutiske produkter -medicinske gasser, oxygen; ATC-kode: V 03 AN 01

5.1 Farmakodynamiske egenskaber
Oxygen udgør cirka 21 % af luften. Oxygen er livsnødvendigt for mennesket og skal kontinuerligt tilføres alle væv for at opretholde cellernes energiproduktion. Oxygen transporteres med inhaleret luft via luftvejene til lungerne. Som en følge af forskellen i partialtryk sker der en gasudveksling i lungealveolerne fra den inhalerede luft-/gasblanding til det kapillære blod. Oxygen transporteres videre i systemisk cirkulation, hovedsageligt bundet til hæmoglobin, til kapillærbanerne i kroppens forskellige væv. Oxygen transporteres ved hjælp af trykgradienten ud til de forskellige celler. Målet er mitokondrierne i de individuelle celler, hvor oxygenet indgår i en enzymatisk kædereaktion, der skaber energi. Ved at øge oxygenfraktionen i den inhalerede luft-/gasblanding øges partialtryksgradienten, som styrer transporten af oxygen til cellerne.

Når oxygen gives ved et tryk, der er højere end det atmosfæriske tryk (HBO), øges mængden af oxygen, der transporteres med blodet til de perifere væv, betydeligt. Intermitterende hyperbar oxygenbehandling forårsager endda oxygentransport i ødematøse væv og væv med utilstrækkelig perfusion og kan på den måde opretholde cellulær energiproduktion og -funktion.

I overensstemmelse med Boyles lov reducerer HBO mængden af gasbobler i væv i relation til trykket, hvormed det gives.

HBO modvirker væksten af anaerobe bakterier.

5.2 Farmakokinetiske egenskaber
Inhaleret oxygen absorberes af en trykafhængig gasudveksling mellem alveolær gas og det kapillære blod, der passerer alveolerne.

Oxygen transporteres vha. den systemiske cirkulation til alle kroppens væv, hovedsageligt reversibelt bundet til hæmoglobin. Kun en meget lille del opløses frit i plasma. Ved passage igennem væv sker der en partialtrykafhængig oxygentransport til de individuelle celler. Oxygen er en vital komponent i cellens intermediære metabolisme. Oxygen er vigtigt for cellens metabolisme bl.a. for at skabe energi igennem aerob ATP-produktion i mitokondrierne.

Oxygen fremskynder udskillelsen af kulilte, der er bundet til hæmoglobin, myoglobin og andre jernoldige proteiner, og modvirker dermed de negative blokerende effekter, der skyldes bindingen af kulilte til jern.

Overtryksbehandling fremskynder yderligere afgivelsen af kulilte sammenlignet med100 % oxygen under normaltryk.

Oxygen, der absorberes i kroppen, elimineres næsten fuldstændigt som kuldioxid dannet i den intermediære metabolisme.

5.3 Prækliniske sikkerhedsdata

Dyreforsøg har vist, at langvarig, kontinuerlig inhalation af ren oxygen kan have skadelige virkninger. Vævsskade kan induceres i lunger, øjne og centralnervesystem. Der er udtalt variabilitet mellem tidspunktet for indtræden af patologiske ændringer blandt forskellige arter og blandt dyr af samme art.

Hyperbar oxygenbehandling under drægtighed hos mus, rotter, hamstere og kaniner medførte øget resorption og fosterabnormaliteter samt nedsat fødselsvægt hos ungerne.

6. FARMACEUTISKE OPLYSNINGER

6.1 Hjælpestoffer

Ingen.

6.2 Uforligeligheder

Ikke relevant.

6.3 Opbevaringstid

3 år for gasflasker ≤ 5 liter
5 år for gasflasker > 5 liter.

6.4 Særlige opbevaringsforhold

Opbevaringsinstruktioner vedrørende lægemidlet
Dette lægemiddel kræver ingen særlige instruktioner vedrørende opbevaringen med hensyn til temperatur ud over dem, der gælder for gasbeholdere og gas under tryk (se nedenfor). Opbevares i et aflåst rum, der er forbeholdt medicinske gasser (gælder ikke hjemmemiljø).

Opbevaringsinstruktioner vedrørende gasbeholdere og gasser under tryk
Brandfarlig ved kontakt med brændbare stoffer.
Holdes væk fra brændbare stoffer.

Rygning forbudt.
Eksplosionsrisiko ved kontakt med olie og fedt.
Må ikke udsættes for stærk varme. Bringes i sikkerhed ved risiko for brand.
Skal håndteres forsigtigt. Må ikke tabes eller udsættes for stød.
Holdes ren og tør. Opbevares på et ventileret sted forbeholdt medicinske gasser.
Opbevares og transporteres med lukket ventil, med beskyttelseshætte og cover, hvis disse forefindes.

6.5 Emballagetyper og pakningsstørrelser

Gasflaskens skulder er markeret med hvid farve (oxygen). Gasflaskens krop er hvid (medicinsk gas).

Beholder (inklusive materiale) og ventiler:
0,5-liters stål-gasbeholder med lukkeventil
0,5-liters stål-gasbeholder med lukkeventil med pin-index
0,5-liters aluminiums-gasbeholder med lukkeventil
0,5-liters aluminiums-gasbeholder med lukkeventil med pin-index

1-liters stål-gasbeholder med lukkeventil
1-liters stål-gasbeholder med lukkeventil med pin-index
1-liters stål-gasbeholder med lukkeventil med indbygget trykregulator
1-liters aluminiums-gasbeholder med lukkeventil
1-liters aluminiums-gasbeholder med lukkeventil med indbygget trykregulator
1-liters aluminiums-gasbeholder med lukkeventil med pin-index
1-liters komposit-gasbeholder med lukkeventil med indbygget manometer/trykmåler

2-liters stål-gasbeholder med lukkeventil
2-liters stål-gasbeholder med lukkeventil med pin-index
2-liters stål-gasbeholder med lukkeventil med indbygget trykregulator
2-liters stål-gasbeholder med lukkeventil med RPV
2-liters aluminiums-gasbeholder med lukkeventil
2-liters aluminiums-gasbeholder med lukkeventil med pin-index
2-liters aluminiums-gasbeholder med lukkeventil med RPV
2-liters aluminiums-gasbeholder med lukkeventil med indbygget trykregulator
2-liters aluminiums-gasbeholder med lukkeventil med indbygget trykregulator og flowregulator (Compact)
2-liters aluminiums-gasbeholder med lukkeventil med indbygget trykregulator og flowregulator (TAKEO)
2-liters aluminiums-gasbeholder med lukkeventil med indbygget trykregulator og flowregulator (0-6 liter/minut) (VIPROXAL)
2-liters aluminiums-gasbeholder med lukkeventil med indbygget trykregulator og flowregulator (0-15 liter/minut) (VIPROXAL)
2-liters aluminiums-gasbeholder med lukkeventil med indbygget trykregulator og flowregulator (0-15 liter/minut) (Oyan).

2,5-liters stål-gasbeholder med lukkeventil
2,5-liters stål-gasbeholder med lukkeventil med pin-index
2,5-liters stål-gasbeholder med lukkeventil med RPV
2,5-liters stål-gasbeholder med lukkeventil med indbygget trykregulator
2,5-liters aluminiums-gasbeholder med lukkeventil
2,5-liters aluminiums-gasbeholder med lukkeventil med pin-index

2,5-liters aluminiums-gasbeholder med lukkeventil med RPV
2,5-liters aluminiums-gasbeholder med lukkeventil med indbygget trykregulator
2,5-liters aluminiums-gasbeholder med lukkeventil med indbygget trykregulator og flowregulator (0-6 liter/minut) (VIPROXAL)
2,5-liters aluminiums-gasbeholder med lukkeventil med indbygget trykregulator og flowregulator (0-15 liter/minut) (VIPROXAL)

3-liters stål-gasbeholder med lukkeventil
3-liters stål-gasbeholder med lukkeventil med pin-index
3-liters stål-gasbeholder med lukkeventil med RPV
3-liters aluminiums-gasbeholder med lukkeventil
3-liters aluminiums-gasbeholder med lukkeventil med pin-index
3-liters aluminiums-gasbeholder med lukkeventil med RPV
3-liters aluminiums-gasbeholder med lukkeventil med indbygget trykregulator og flowregulator (TAKEO)
3-liters aluminiums-gasbeholder med lukkeventil med indbygget trykregulator og flowregulator (0-15 liter/minut) (VIPROXAL)
3-liters aluminiums-gasbeholder med lukkeventil med indbygget trykregulator og flowregulator (0-25 liter/minut) (VIPROXAL)
3-liters aluminiums-gasbeholder med lukkeventil med indbygget trykregulator og flowregulator (0-15 liter/minut) (Oyan).

3,5-liters stål-gasbeholder med lukkeventil
3,5-liters stål-gasbeholder med lukkeventil med pin-index
3,5-liters stål-gasbeholder med lukkeventil med RPV
3,5-liters aluminiums-gasbeholder med lukkeventil
3,5-liters aluminiums-gasbeholder med lukkeventil med pin-index
3,5-liters aluminiums-gasbeholder med lukkeventil med RPV

4-liters stål-gasbeholder med lukkeventil
4-liters stål-gasbeholder med lukkeventil med pin-index
4-liters stål-gasbeholder med lukkeventil med RPV
4-liters aluminiums-gasbeholder med lukkeventil
4-liters aluminiums-gasbeholder med lukkeventil med pin-index
4-liters aluminiums-gasbeholder med lukkeventil med RPV

4,75-liters stål-gasbeholder med lukkeventil med pin-index

5-liters stål-gasbeholder med lukkeventil
5-liters stål-gasbeholder med lukkeventil med pin-index
5-liters stål-gasbeholder med lukkeventil med RPV
5-liters stål-gasbeholder med lukkeventil med indbygget trykregulator
5-liters aluminiums-gasbeholder med lukkeventil
5-liters aluminiums-gasbeholder med lukkeventil med pin-index
5-liters aluminiums-gasbeholder med lukkeventil med RPV
5-liters aluminiums-/alternativt stål-gasbeholder med lukkeventil med indbygget trykregulator og flowmåler (Compact)
5-liters aluminiums-gasbeholder med lukkeventil med indbygget trykregulator og flowmåler (TAKEO)
5-liters aluminiums-gasbeholder med lukkeventil med indbygget trykregulator og flowmåler (0-6 liter/minut) (VIPROXAL)

5-liters aluminiums-gasbeholder med lukkeventil med indbygget trykregulator og flowmåler (0-15 liter/minut) (VIPROXAL)
5-liters aluminiums-gasbeholder med lukkeventil med indbygget trykregulator og flowregulator (0-15 liter/minut) (Oyan).

6-liters stål-gasbeholder med lukkeventil
6-liters stål-gasbeholder med lukkeventil med pin-index
6-liters stål-gasbeholder med lukkeventil med RPV
6-liters aluminiums-gasbeholder med lukkeventil
6-liters aluminiums-gasbeholder med lukkeventil med pin-index
6-liters aluminiums-gasbeholder med lukkeventil med RPV

7-liters stål-gasbeholder med lukkeventil
7-liters stål-gasbeholder med lukkeventil med pin-index
7-liters stål-gasbeholder med lukkeventil med RPV
7-liters aluminiums-gasbeholder med lukkeventil
7-liters aluminiums-gasbeholder med lukkeventil med pin-index
7-liters aluminiums-gasbeholder med lukkeventil med RPV

8-liters stål-gasbeholder med lukkeventil
8-liters stål-gasbeholder med lukkeventil med pin-index
8-liters stål-gasbeholder med lukkeventil med RPV
8-liters aluminiums-gasbeholder med lukkeventil
8-liters aluminiums-gasbeholder med lukkeventil med pin-index
8-liters aluminiums-gasbeholder med lukkeventil med RPV
8-liters aluminiums-gasbeholder med lukkeventil med indbygget trykregulator og flowregulator (Compact)
8-liters aluminiums-gasbeholder med lukkeventil med indbygget digital trykregulator og flowregulator (TAKEO)

10-liters stål-gasbeholder med lukkeventil
10-liters stål-gasbeholder med lukkeventil med pin-index
10-liters stål-gasbeholder med lukkeventil med RPV
10-liters stål-gasbeholder med lukkeventil med indbygget trykregulator og flowregulator
10-liters stål-gasbeholder med lukkeventil med indbygget trykregulator og flowmåler (0-15 liter/minut) (VIPROXAL)
10-liters stål-gasbeholder med lukkeventil med indbygget trykregulator og flowmåler (0-25 liter/minut) (VIPROXAL)
10-liters stål-gasbeholder med lukkeventil med indbygget trykregulator og flowregulator (0-15 liter/minut) (Oyan)

10-liters aluminiums-gasbeholder med lukkeventil
10-liters aluminiums-gasbeholder med lukkeventil med pin-index
10-liters aluminiums-gasbeholder med lukkeventil med RPV
10-liters aluminiums-gasbeholder med lukkeventil med indbygget trykregulator og flowregulator (Compact)
10-liters aluminiums-gasbeholder med lukkeventil med indbygget digital trykregulator og flowregulator (TAKEO)
10-liters aluminiums-gasbeholder med lukkeventil med indbygget trykregulator og flowmåler (0-15 liter/minut) (VIPROXAL)

10-liters aluminiums-gasbeholder med lukkeventil med indbygget trykregulator og flowmåler (0-25 liter/minut) (VIPROXAL)
10-liters aluminiums-gasbeholder med lukkeventil med indbygget trykregulator og flowregulator (0-15 liter/minut) (Oyan)

11-liters stål-gasbeholder med lukkeventil
11-liters stål-gasbeholder med lukkeventil med RPV
11-liters stål-gasbeholder med lukkeventil med indbygget trykregulator og flowregulator
11-liters aluminiums-gasbeholder med lukkeventil
11-liters aluminiums-gasbeholder med lukkeventil med RPV
11-liters aluminiums-gasbeholder med lukkeventil med indbygget trykregulator og flowregulator (Compact)
11-liters aluminiums-gasbeholder med lukkeventil med indbygget digital trykregulator og flowregulator (TAKEO)
11-liters aluminiums-gasbeholder med lukkeventil med indbygget trykregulator og flowregulator (0-15 liter/minut) (Oyan).

15-liters stål-gasbeholder med lukkeventil
15-liters aluminiums-gasbeholder med lukkeventil
15-liters aluminiums-/alternativt stål-gasbeholder med lukkeventil med indbygget trykregulator og flowmåler (Compact)
15-liters aluminiums-gasbeholder med lukkeventil med indbygget digital trykregulator og flowregulator (TAKEO)
15-liters aluminiums-gasbeholder med lukkeventil med indbygget trykregulator og flowregulator (0-15 liter/minut) (Oyan).

16-liters stål-gasbeholder med lukkeventil
16-liters aluminiums-gasbeholder med lukkeventil

20-liters stål-gasbeholder med lukkeventil
20-liters stål-gasbeholder med lukkeventil med RPV
20-liters stål-gasbeholder med lukkeventil med indbygget trykregulator
20-liters aluminiums-gasbeholder med lukkeventil
20-liters aluminiums-gasbeholder med lukkeventil med indbygget trykregulator og flowregulator

28-liters stål-gasbeholder med lukkeventil

30-liters stål-gasbeholder med lukkeventil
30-liters stål-gasbeholder med lukkeventil med indbygget trykregulator
30-liters aluminiums-gasbeholder med lukkeventil
30-liters aluminiums-gasbeholder med lukkeventil med indbygget trykregulator og flowregulator

40-liters stål-gasbeholder med lukkeventil

50-liters stål-gasbeholder med lukkeventil
50-liters stål-gasbeholder med lukkeventil med indbygget trykregulator
50-liters stål-gasbeholder med lukkeventil med RPV
50-liters aluminiums-gasbeholder med lukkeventil

50-liters aluminiums-gasbeholder med lukkeventil med indbygget trykregulator og flowregulator
50-liters aluminiums-gasbeholder med lukkeventil med RPV

12x40-liters stål-gasbeholder med lukkeventil
10x50-liters stål-gasbeholder med lukkeventil
15x40-liters stål-gasbeholder med lukkeventil
12x50-liters stål-gasbeholder med lukkeventil
20x40-liters stål-gasbeholder med lukkeventil
20x50-liters stål-gasbeholder med lukkeventil

Ikke alle pakningsstørrelser er nødvendigvis markedsført.

Gasbeholder/batteri fyldt til 200 bar leverer cirka X kubikmeter (m^3) gas ved atmosfærisk tryk og 15° C i henhold til tabellen nedenfor:

Beholder-størrelse i liter	0,5	1	2	2,5	3	3,5	4	4,75	5	6	7
Kubikmeter gas (m^3)	0,1	0,2	0,4	0,5	0,6	0,7	0,8	1,0	1,1	1,3	1,5

Beholder-størrelse i liter	8	10	11	15	16	20	28	30	40	50
Kubikmeter gas (m^3)	1,7	2,1	2,3	3,2	3,4	4,2	5,8	6,2	8,4	11

Batteri-størrelse i liter	12x40	10x50	15x40	12x50	20x40	20x50
Kubikmeter gas (m^3)	$1,0 \times 10^2$	$1,1 \times 10^2$	$1,3 \times 10^2$	$1,3 \times 10^2$	$1,7 \times 10^2$	$2,1 \times 10^2$

6.6 Regler for destruktion og anden håndtering

Generelt
Medicinske gasser må kun anvendes til medicinske formål.

Forskellige gastyper og gaskvaliteter skal adskilles fra hinanden. Fulde og tomme gasbeholdere skal opbevares adskilt.

Anvend aldrig olie eller fedt, selvom flaskeventilen er stram, eller hvis regulatoren er svær at tilslutte. Håndter ventiler og dertil hørende apparater med rene og fedtfri (håndcreme osv.) hænder.

Anvend kun standardudstyr, der er beregnet til medicinsk oxygen.

Kontroller, at flaskerne er forseglede, før de anvendes.

Før enhver anvendelse skal det sikres, at flasken indeholder en tilstrækkelig mængde af produktet til at sikre fuldførelse af den planlagte administration.

Beholdere med en såkaldt Compact-, Viproxal-, Takeo- og Oyan ventiler har en indbygget trykregulator i ventilen. Derfor er en separat trykregulator unødvendig. Compact-, Viproxal-, Takeo- og Oyan-ventilerne har en standardlynkobling til tilslutning af specifikt udstyr, men også separat udgang til konstant flow, hvor flowet kan reguleres til det ønskede niveau.

Forberedelse til brug

Fjern forseglingen fra ventilen før brug.

Anvend kun regulatorer, der er beregnet til medicinsk oxygen. Kontroller, at den automatiske sammenkobling eller regulator er ren, og at pakningerne er i god stand.

Anvend aldrig værktøj på en tryk-/flowregulator, der sidder fast, hvis den er beregnet til at skulle tilsluttes manuelt, da det kan beskadige sammenkoblingen.

Åbn beholderventilen langsomt – mindst en halv omgang.

Forsøg ikke at håndtere lækager fra ventilen eller apparatet selv, ud over ved udskiftning af pakning eller O-ring.

I tilfælde af lækage skal ventilen lukkes og regulatoren frakobles. Mærk defekte beholdere, sæt dem til side, og returner dem til leverandøren.

Anvendelse af gasbeholderen
Rygning og åben ild er strengt forbudt i rum, hvor der udføres oxygenbehandling.

Luk beholderen ved brand, eller hvis den ikke anvendes.

Bringes i sikkerhed ved brand.

Større gasbeholdere skal transporteres på en flaskevogn af passende type. Vær specielt opmærksom på, at tilsluttede apparater ikke løsnes utilsigtet.

Når beholderen anvendes, skal den sidde fast i en passende holder.

For beholdere med indbygget ventil skal brugeren forberede sig på at udskifte flasken, når trykindikatoren er inden for det gule felt, og udskifte den, når indikatoren når ind i det røde felt.

Når der er en lille mængde gas tilbage i gasbeholderen (cirka 2 bar), skal ventilen lukkes. Det er vigtigt at efterlade et lille tryk i beholderen for at beskytte den imod kontaminering.

Efter brug skal ventilen lukkes med normal kraft. Fjern trykket fra regulatoren eller tilslutningen.

7. INDEHAVER AF MARKEDSFØRINGSTILLADELSEN

Air Liquide Santé International
75 Quai d' Orsay
F-75007 Paris
Frankrig

Repræsentant
Air Liquide Gas AB
Lundavägen 151
212 24 Malmö
Sverige

8. MARKEDSFØRINGSTILLADELSESNUMMER (NUMRE)
49225

9. DATO FOR FØRSTE MARKEDSFØRINGSTILLADELSE
18. november 2010

10. DATO FOR ÆNDRING AF TEKSTEN
18. juli 2022

Appendix 4 SmPC for Compressed air (Medicinsk Luft ”Air Liquide”)

LÆGEMIDDELSTYRELSEN
DANISH MEDICINES AGENCY

30. marts 2021

PRODUKTRESUMÉ

for

Medicinsk Luft ”Air Liquide” 100 %, medicinsk gas, komprimeret

0. D.SP.NR.
27953

1. LÆGEMIDLETS NAVN
Medicinsk Luft ”Air Liquide” 100 %

2. KVALITATIV OG KVANTITATIV SAMMENSÆTNING
Medicinsk luft 100 % ved et tryk på 200 bar (15 °C).

3. LÆGEMIDDELFORM
Medicinsk gas, komprimeret.

4. KLINISKE OPLYSNINGER

4.1 Terapeutiske indikationer
Medicinsk luft er indiceret som erstatning for normal omgivelses-/rumluft, når der er behov herfor, f.eks.:

- Ved respiratorbehandling eller i forbindelse med anæstesi som en del af friskgasflowet for at tilføre en gasblanding med det ønskede oxygenindhold (FiO_2).
- Som drivgas i nebulisatorbehandling.
- Som ren luft til behandling af immunsupprimerede patienter, f.eks. ved organ-/celletransplantation eller omfattende brandsår.

4.2 Dosering og indgivelsesmåde

Dosering
Medicinsk luft kan anvendes til børn, voksne og ældre. Formålet med at bruge medicinsk luft er at sikre en pålidelig tilførsel af gas, som indeholder oxygen i en koncentration, der svarer til den normale omgivelses-/rumluft uden risiko for tilblanding af lugte eller andre potentielt irriterende stoffer. Medicinsk luft er kun indiceret til erstatning for rumluft. Hvis der er behov kan Medicinsk luft blandes med medicinsk oxygen, således at den ønskede oxygenkoncentration opnås på baggrund af følgende formel:

FiO_2 = [(antal liter luft/minut x 21) + (antal liter oxygen/minut x 100)] / (antal liter luft/minut + antal liter oxygen/minut)

Administration
Medicinsk luft tilføres via indåndingsluften.
Medicinsk luft tilføres med specialudstyr. Ved hjælp af dette udstyr tilføres den medicinske luft til den gas, der skal indåndes, og ved udånding blandes den luft, som ikke er blevet absorberet, med den omgivende luft (ikke-genindåndingssystem). Især ved anæstesi anvendes ofte specialudstyr, som bevirker, at en større eller mindre del af den udåndede gas kan recirkuleres i respirationssystemet og genindåndes (såkaldt genindåndingssystem).

For information om brug og håndtering se pkt. 6.6

4.3 Kontraindikationer
Ingen kendte

4.4 Særlige advarsler og forsigtighedsregler vedrørende brugen
Ingen kendte

4.5 Interaktion med andre lægemidler og andre former for interaktion
Ingen kendte interaktioner.

4.6 Graviditet og amning
Medicinsk luft må anvendes under graviditet og amning.

4.7 Virkninger på evnen til at føre motorkøretøj eller betjene maskiner
Ikke mærkning.
Ikke relevant.

4.8 Bivirkninger

Indberetning af mistænkte bivirkninger
Når lægemidlet er godkendt, er indberetning af mistænkte bivirkninger vigtig. Det muliggør løbende overvågning af benefit/risk-forholdet for lægemidlet. Sundhedspersoner anmodes om at indberette alle mistænkte bivirkninger via:
Lægemiddelstyrelsen
Axel Heides Gade 1
DK-2300 København S
Websted: www.meldenbivirkning.dk

4.9 Overdosering
Ikke relevant.

4.10 Udlevering
GH

5. FARMAKOLOGISKE EGENSKABER

5.0 Terapeutisk klassifikation

Farmakoterapeutisk klassifikation: Medicinske gasser; ATC-kode: V 03 AN 05

5.1 Farmakodynamiske egenskaber

Medicinsk luft indeholder 21 % oxygen, og den resterende del er nitrogengas, der må betragtes som inert. Medicinsk luft anvendes primært på grund af sit oxygenindhold, som svarer fuldstændigt til rumluft.

Oxygen er livsnødvendigt for mennesket og skal kontinuerligt tilføres alle væv for at opretholde cellernes energiproduktion. Målet er mitokondrierne i de individuelle celler, hvor oxygenet deltager i en enzymatisk kædereaktion, der skaber energi, aerob metabolisme.

Nitrogen kan betragtes som inert.

5.2 Farmakokinetiske egenskaber

Medicinsk luft består af 21 % oxygen, hvilket svarer fuldstændigt til koncentrationen i normal rum-/omgivelsesluft. Det administreres ved inhalation og transporteres via luftvejene til lungerne. Som en følge af forskellen i partialtryk sker der en gasudveksling i lungealveolerne fra den inhalerede luft-/gasblanding til det kapillære blod. Oxygen transporteres videre i systemisk cirkulation, hovedsageligt bundet til hæmoglobin og en meget lille andel er opløst i plasma, til kapillærbanerne i kroppens mange forskellige væv. Oxygen transporteres ved hjælp af trykgradienten ud til de forskellige celler.

Oxygen, der absorberes i kroppen, elimineres næsten fuldstændigt som kuldioxid dannet ved den intermediære metabolisme.

Nitrogen absorberes ikke og følger udåndingsluften uden at have gennemgået nogen omdannelse/metabolisme.

5.3 Prækliniske sikkerhedsdata

Ikke relevant.

6. FARMACEUTISKE OPLYSNINGER

6.1 Hjælpestoffer

Ingen.

6.2 Uforligeligheder

Ikke relevant.

6.3 Opbevaringstid

3 år for gasflasker ≤ 5 liter.
5 år for gasflasker > 5 liter.

6.4 Særlige opbevaringsforhold

Opbevares i et rum, der er forbeholdt medicinske gasser (gælder ikke hjemmemiljø).
Skal håndteres forsigtigt. Må ikke tabes eller udsættes for stød.
Opbevares og transporteres med lukket ventil, samt beskyttelseshætte og cover, hvis disse forefindes.

6.5 Emballagetyper og pakningsstørrelser

Gasbeholdrens skulder er markeret med sort og hvid farve (luft). Gasbeholderens krop er hvid (medicinsk gas).

Beholder (inklusive materiale) og ventiler:

1-liters stål-gasbeholder med lukkeventil.
1-liters aluminiums-gasbeholder med lukkeventil.
2-liters stål-gasbeholder med lukkeventil.
2-liters aluminiums-gasbeholder med lukkeventil.
2,5-liters stål-gasbeholder med lukkeventil.
2,5-liters aluminiums-gasbeholder med lukkeventil.
2,5-liters stål-gasbeholder med lukkeventil med pin-index.
2,5-liters stål-gasbeholder med lukkeventil med indbygget trykregulator.
4-liters stål-gasbeholder med lukkeventil.
4-liters stål-gasbeholder med lukkeventil med pin-index.
5-liters stål-gasbeholder med lukkeventil.
5-liters aluminiums-gasbeholder med lukkeventil.
5-liters aluminiums-gasbeholder med lukkeventil med indbygget trykregulator.
10-liters stål-gasbeholder med lukkeventil.
10-liters aluminiums-gasbeholder med lukkeventil.
20-liters stål-gasbeholder med lukkeventil.
20-liters aluminiums-gasbeholder med lukkeventil.
40-liters stål-gasbeholder med lukkeventil.
50-liters stål-gasbeholder med lukkeventil.
50-liters aluminiums-gasbeholder med lukkeventil.

10x50-liters stål-gasbeholder med lukkeventil.
12x50-liters stål-gasbeholder med lukkeventil.
15x40-liters stål-gasbeholder med lukkeventil.

Ikke alle pakningsstørrelser er nødvendigvis markedsført.

Gasbeholder/batteri fyldt til 200 bar leverer cirka X kubikmeter (m^3) gas ved atmosfærisk tryk og 15° C i henhold til tabellen nedenfor:

Beholder-størrelse i liter	1	2	2,5	4	5	10	20	40	50
Kubikmeter gas (m^3)	0,2	0,4	0,5	0,8	1,0	2,0	4,0	8,0	9,9

Batteri-størrelse i liter	10x50	12x50	15x40
Kubikmeter gas (m^3)	$1{,}0 \times 10^2$	$1{,}2 \times 10^2$	$1{,}2 \times 10^2$

6.6 Regler for destruktion og anden håndtering

Generelt
Medicinske gasser må kun anvendes til medicinske formål.

Forskellige gastyper og gaskvaliteter skal adskilles fra hinanden. Fulde og tomme gasbeholdere skal opbevares adskilt.

Anvend aldrig olie eller fedt, selvom flaskeventilen er stram, eller hvis regulatoren er svær at tilslutte. Håndter ventiler og dertil hørende udstyr med rene og fedtfri (håndcreme osv.) hænder.

Anvend kun standardudstyr, der er beregnet til medicinsk luft.

Gasbeholderne skal opbevares beskyttet imod vind og vejr og holdes tørre og rene.

Kontroller, at beholderne er forseglede, før de anvendes.

Forberedelse til brug
Fjern forseglingen fra ventilen før brug.

Anvend kun regulatorer, der er beregnet til medicinsk luft. Kontroller, at forbindelsen på ydersiden af kobling eller regulator er ren, og at forbindelserne er i god stand.

Anvend aldrig værktøj på en tryk-/flowregulator, der sidder fast, hvis den er beregnet til at skulle tilsluttes manuelt, da det kan beskadige sammenkoblingen.

Åbn flaskeventilen langsomt – mindst en halv omgang. Forsøg ikke at håndtere lækager fra ventilen eller apparatet selv, ud over ved udskiftning af pakning eller O-ring.

I tilfælde af lækage skal ventilen lukkes og regulatoren frakobles. Mærk defekte beholdere, sæt dem til side på et sted tilegnet reklamationer, og returner dem til leverandøren.

Anvendelse af gasbeholderen
Rygning og åben ild er strengt forbudt i rum, hvor der udføres behandling med medicinsk luft. Luk beholderen ved brand, eller hvis den ikke anvendes.

Større gasbeholdere skal transporteres på en flaskevogn af passende type. Vær specielt opmærksom på, at tilsluttede apparater ikke løsnes utilsigtet.

Når beholderen er i brug, skal den sidde fast i en passende holder.

Når der er en lille mængde gas tilbage i gasbeholderen, skal ventilen lukkes. Det er vigtigt at efterlade et lille tryk i beholderen for at beskytte den imod kontaminering.

Efter brug skal ventilen lukkes med håndkraft. Fjern trykket fra regulatoren eller tilslutningen.

7. INDEHAVER AF MARKEDSFØRINGSTILLADELSEN
Air Liquide Santé International

75 Quai d' Orsay
F-75007 Paris
Frankrig

Repræsentant
Air Liquide Gas AB
Lundavägen 151
212 24 Malmö
Sverige

8. MARKEDSFØRINGSTILLADELSESNUMMER (NUMRE)

49227

9. DATO FOR FØRSTE MARKEDSFØRINGSTILLADELSE

28. oktober 2011

10. DATO FOR ÆNDRING AF TEKSTEN

19. marts 2021

Appendix 5 Marking of compressed air in ambulances

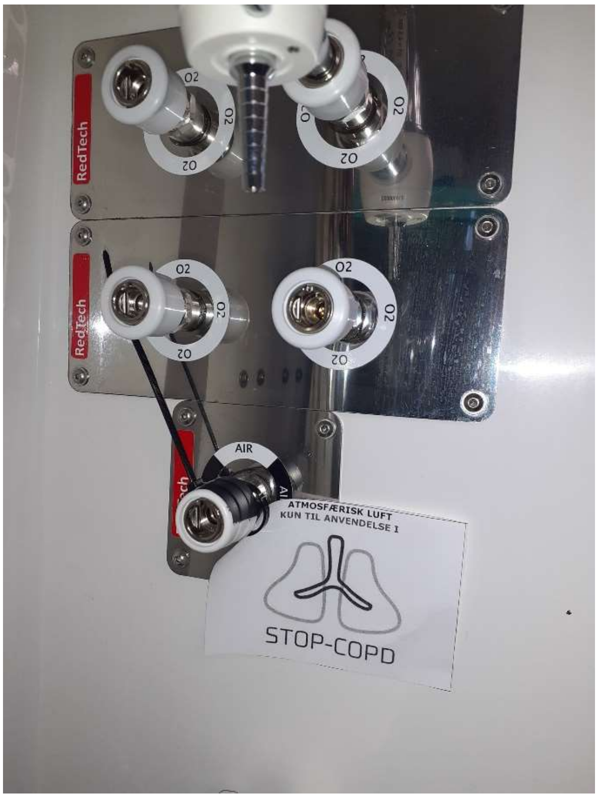

Figur 1 Example on marking of Medicinsk Luft "Air Liquide" (Compressed Air) outlet in ambulance

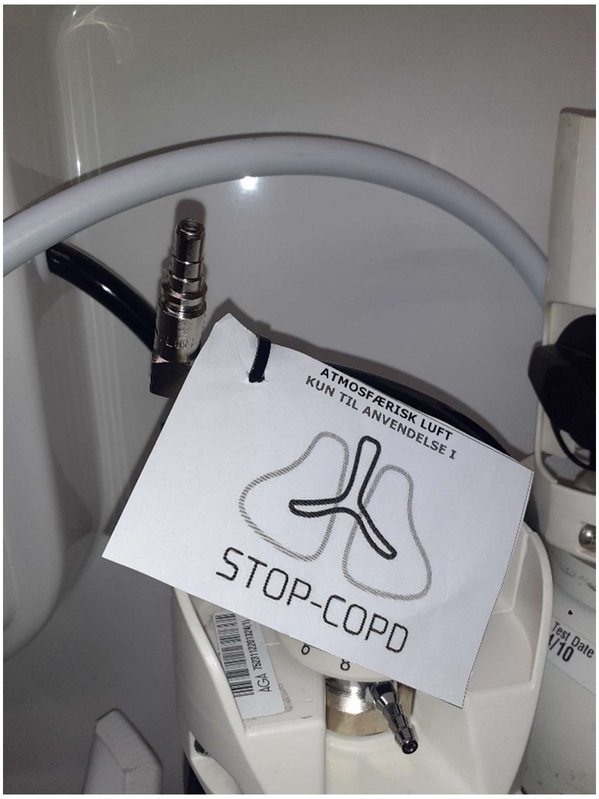

Figur 2 Example on marking of Medicinsk Luft "Air Liquide" (Compressed Air) inlet in ambulance

Appendix 6 Labelling of Oxygen

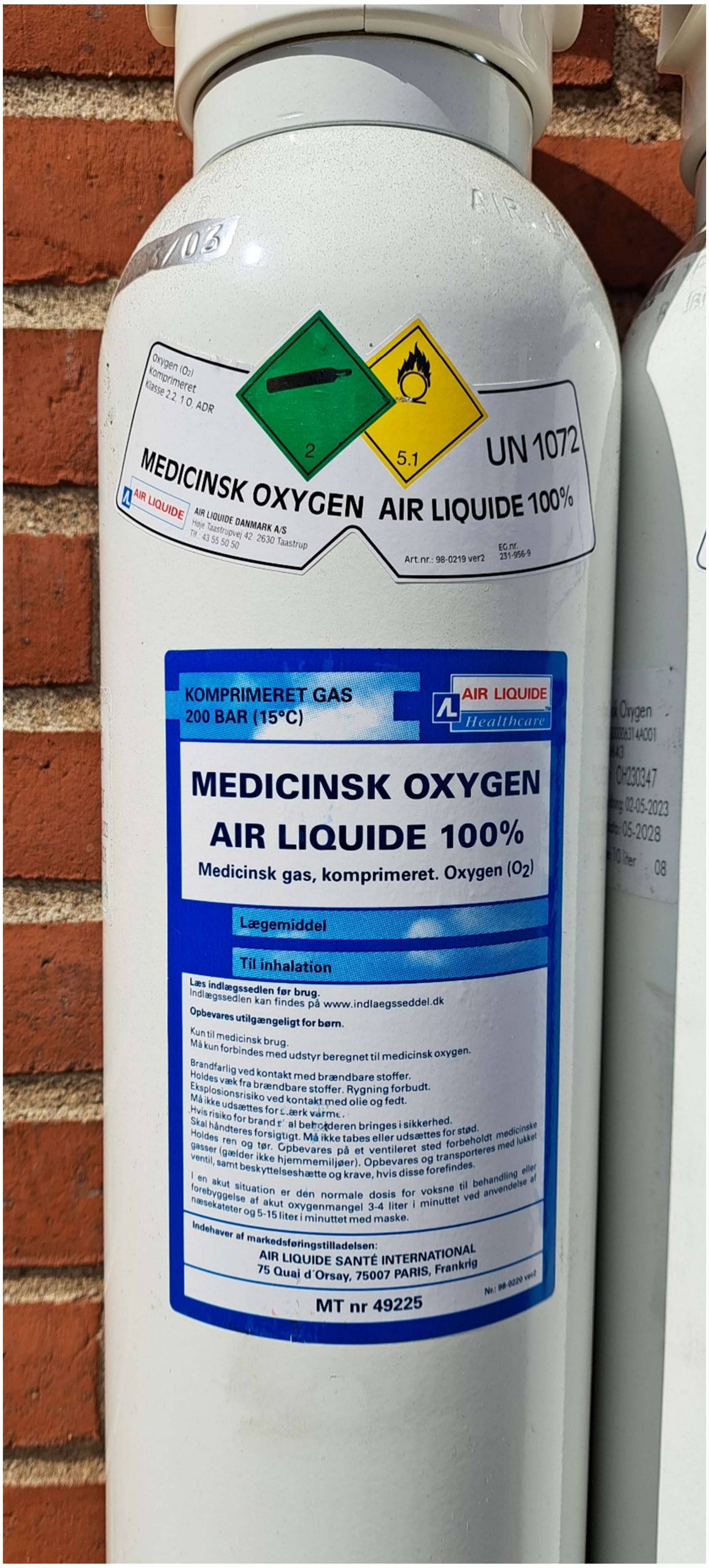

Appendix 7 Labelling of Air

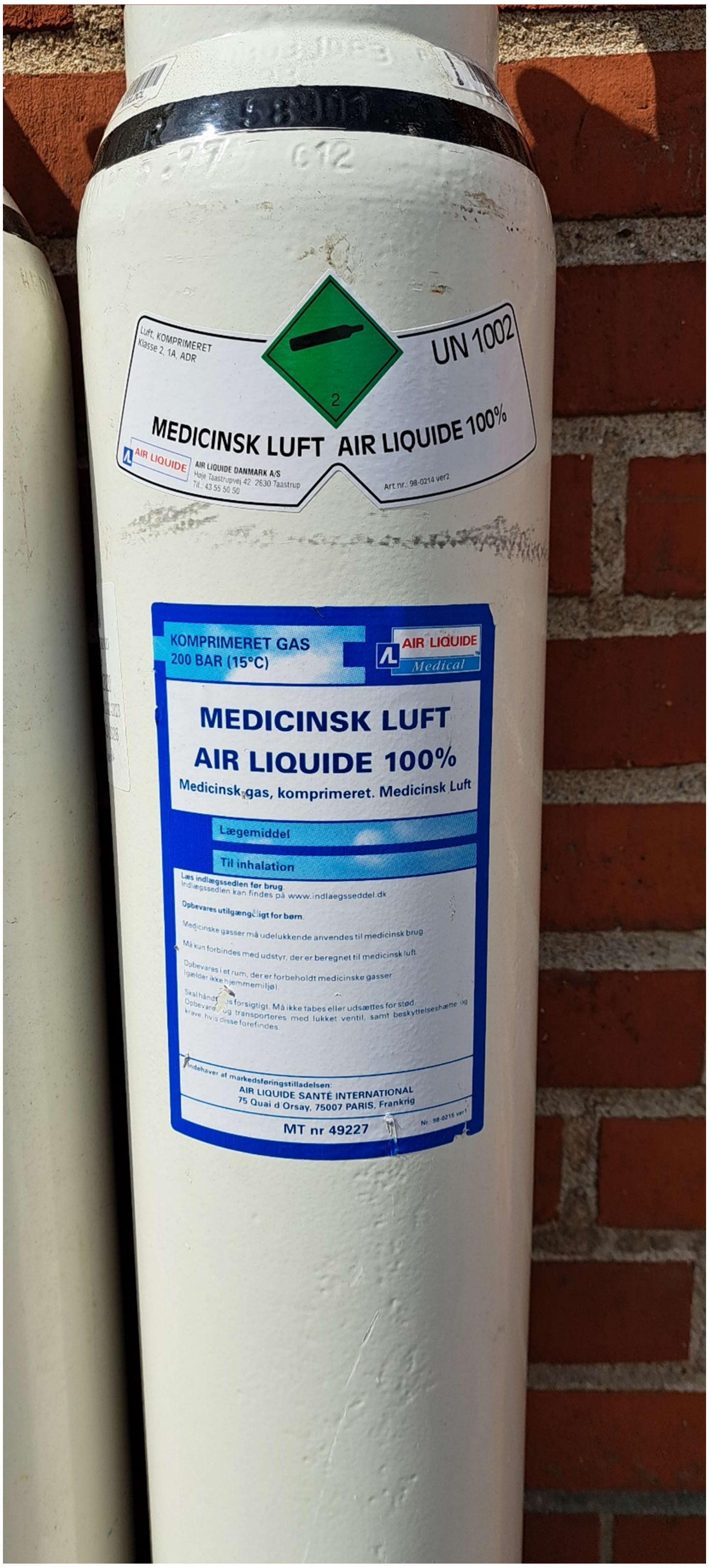

Participant consent form (in Danish)

Deltagerinformation om deltagelse i et videnskabeligt forsøg

Forsøgets titel: **STOP-COPD** – **S**tandard vs **T**argeted **O**xygen Therapy **P**rehospital for **C**hronic **O**bstructive **P**ulmonary **D**isease

Dansk oversættelse: Standard vs målrettet iltbehandling præhospitalt til kronisk obstruktiv lungelidelse

EU CTA nummer: 2022-502003-30-00

Vi vil spørge, om du vil deltage i et videnskabeligt forsøg, der udføres af Forskningsansvarlig læge Martin Faurholdt Gude og Paramediciner Arne Sylvester Rønde Jensen, Afdeling for Forskning & Udvikling, Præhospitalet, Region Midt.

Før du beslutter, om du vil deltage i forsøget, skal du fuldt ud forstå, hvad forsøget går ud på, og hvorfor vi gennemfører forsøget. Vi vil derfor bede dig om at læse denne deltagerinformation grundigt.
Efter du har læst informationen vil du blive informeret mundtligt om forsøget, hvor denne deltagerinformation vil blive uddybet, og hvor du kan stille de spørgsmål, du har om forsøget. Du er velkommen til at tage en bisidder med til samtalen, det kan f.eks. være en pårørende, ven eller et personale fra afdelingen.

Hvis du beslutter dig for at deltage i forsøget, vil vi bede dig om at underskrive en elektronisk samtykkeerklæring. Husk, at du har ret til betænkningstid, før du beslutter, om du vil underskrive samtykkeerklæringen.

Det er frivilligt at deltage i forsøget. Du kan når som helst og uden at give en grund trække dit samtykke tilbage. Det vil ikke få konsekvenser for din videre behandling.

Formål med forsøget

Formålet med forskningsprojektet er at undersøge om man kan sænke risikoen for komplikationer af KOL i forværring ved at målrette mængden af ilt der gives i ambulancen på vej til hospitalet.

Participant consent form (in Danish)

Baggrund for forsøget

Ved KOL i forværring er der dele af luftvejene i lungerne der er forsnævrede enten pga. sammentrækning eller pga. øget slim og sekret. Derfor gives inhalationsmedicin for at afslappe luftvejene og dermed skabe mere plads så vejrtrækningen lettes. Denne behandling opstartes allerede i ambulancerne og forsættes på hospitalet.
Normal bliver inhalationsmedicinen forstøvet ved hjælp af ilt i en forstøvermaske. En del forskning har vist at der kan være flere negative konsekvenser ved at modtage store mængder ilt over længere tid. Derfor vil vi i dette projekt undersøge om det kan have positive effekter at bruge atmosfærisk luft til forstøvningen i stedet for ilt.

Forsøgsbehandlingen foregår kun i ambulancerne og er derfor slut når du ankommer til hospitalet. Forskerne har i en periode på op til 100 dage efter din indlæggelse lov til at indhente oplysninger i din journal. Herefter er din deltagels i forsøget afsluttet.

Forsøgspersonener i projektet vil blive tilfældigt fordelt i to grupper af 944 patienter, dermed når det samlede antal deltagende patienter op på 1.888.
Den ene gruppe får den normal behandling, det vil sige inhalations medicin forstøvet ved hjælp at ilt.
Den anden gruppe får forsøgsbehandling, her forstøves inhalationsmedicinen af atmosfærisk luft, hvis en patients iltmætning er meget lav vil der blive givet et tilskud af ilt, tilpasset efter den enkelte patients behov.
Tildelingen foregår elektronisk og kan ikke påvirkes af ambulancepersonalet, forskningspersonalet eller andre. Patienterne i forsøget er blindet, dvs. at de ikke ved hvilken behandling de tildeles.

Grunden til at du først høre om forsøget nu er at det er godkendt som et akutforsøg. Det betyder at man i akutte situationer hvor det ikke er muligt at informere en patient mundtligt, skrifteligt og give tilpas betænkningstid, pga. behovet for hurtig behandling, kan få lov til at gøre dette efter behandlingen er overstået.

Hvis du samtykker til at deltage i forsøget giver du forskerene lov til at indhente nogle af de målinger der er lavet i ambulancen samt lige da du ankom til hospitalet, det kan f.eks. være blodtryk, puls og iltmætning. Du giver også lov til at forskerene må se i din journal om du får nogle komplikationer i hospitalsforløbet og i de næste 30 dage. Her er der tale om oplysninger så som indlæggelsestid, intensiv behandling, genindlæggelse og udskrivelsesdiagnose, disse oplysninger vil blive anvendt til at vurdere effekten af behandlingen.

Participant consent form (in Danish)

Forskningsprojektet bliver løbende monitoreret af GCP-enheden ved Aarhus Universitet, derudover kan der komme kontrolbesøg fra Datatilsynet, Lægemiddelstyrelse og Etisk komite, disse vil også få adgang til dine journaloplysninger.
Alle med adgang til personoplysninger er underlagt tavshedspligt.

Hvis du ikke giver samtykke til at deltage i forsøget vil der ikke blive indhentet yderligere oplysninger og allerede indsamlet data vil blive slettet. Det vil ikke have indflydelse på dit videre behandlingsforløb. Alle personfølsomme data opbevares og behandles efter databeskyttelsesloven og databeskyttelsesforordningen.

Nytte ved forsøget

Hvis forsøgsbehandling viser sig at være som vi forventer, vil det betyde kortere indlæggelsestid, færre dage på intensiv, færre der skal i respirator og færre der dør under hospitalsopholdet.
Det kan komme dig til gode hvis du skulle få KOL i forværring en anden gang eller det kan komme nogle af de mange tusind andre patienter der hvert år oplever det samme til gode.

Bivirkninger, risici, komplikationer og ulemper

Der er ikke nogle kendte bivirkninger til atmosfærisk luft.
Der kan være risici ved forsøget, som vi endnu ikke kender. Vi beder dig derfor om at fortælle, hvis du oplever problemer med dit helbred, mens forsøget står på. Hvis vi opdager bivirkninger, som vi ikke allerede har fortalt dig om, vil du naturligvis blive orienteret med det samme, og du vil skulle tage stilling til, om du ønsker at fortsætte i forsøget.

Andre behandlingsmuligheder

Du vil både i ambulance og på hospitalet blive behandlet efter de guidelines og retningslinjer der er for patienter med KOL i forværring, fraset måden inhalationsmedicinen blive forstøvet i ambulancen.

Participant consent form (in Danish)

Udelukkelse fra og afbrydelse af forsøg

Hvis der før inklusion i forsøget har været behov for avanceret behandling af en akutlægebil kan du desværre ikke deltage i dette forsøg.

Oplysninger om økonomiske forhold

Paramediciner Arne Sylvester Jensen har taget initiativ til projektet i samarbejde med læge Martin Faurholdt Gude og Præhospitalet som organisation.

Der er givet støtte fra følgende fonde:

- “Den Landsdækkende Akutlægehelikopterordning" kr. 90.478,-
- "Simon Spies Fonden" kr. 15.000,-
- "Eva Merete Falck Crones Fond" kr. 50.000,-
- "Region Midtjyllands Strategiske Forskningspulje" kr. 1.175.000,-

Der er og bliver ansøgt flere offentlige og private fonde om yderligere støtte. Alle støttebeløb udbetales til en projektkonto ved Præhospitalets økonomiafdeling som også står for at administrere pengene. Bidragsydere har ikke indflydelse på planlægning, afvikling eller afrapportering af forsøget.
Der gives ikke vederlag til forsøgspersoner der deltager i dette forsøg.

Adgang til forsøgsresultater

Når forsøget er afsluttet, senest d. 08/01/2026, vil der blive skrevet en forskningsartikel til et medicinsk tidsskrift. Denne forskningsartikel vil kunne læses af alle interesserede (open acces). Forsøgets resultater vil også blive præsenteret på en relevant konference. Forsøgets resultater og et letlæseligt resumé vil være tilgængelige i EU Clinical Trials databasen, efter forsøgets afslutning. Hvis du er interesseret i at følge projektet kan du se mere på www.stop-copd.com. Hvis du gerne vil have resultaterne tilsendt på e-mail gør vi gerne det, oplyse det da venligst til vedkommende der indhenter samtykket. Du har samtidig mulighed for at blive informeret om hvilken behandling du blev tildelt hvis du ønsker det.
Vi forventer at kunne præsentere resultaterne i starten af 2026.
Anonymiseret data fra forskningsprojektet kan blive delt med andre forskere i henhold til dansk lovgivning.

Participant consent form (in Danish)

Yderligere information og kontakt

Vi håber, at du med denne information har fået tilstrækkeligt indblik i, hvad det vil sige at deltage i forsøget, og at du føler dig rustet til at tage beslutningen om din eventuelle deltagelse. Vi beder dig også om at læse det vedlagte materiale "Forsøgspersonens rettigheder i et sundhedsvidenskabeligt forskningsprojekt".

Hvis du vil vide mere om forsøget, er du meget velkommen til at kontakte undertegnede.

Med venlig hilsen

Kontaktperson:
Paramediciner Arne Sylvester Rønde Jensen
Præhospitalet
Olof Palmes Alle 34 1. sal
8200 Aarhus N
Tlf: 22396968
E-mail: arjens@rm.dk

Forsøgsansvarlig:
Læge Matin Faurholdt Gude
Præhospitalet
Olof Palmes Alle 34 1. sal
8200 Aarhus N

Participant consent form (in Danish)

Informeret samtykke til deltagelse i forsøget:

Standart vs. målrettet iltbehandling til kronisk obstruktiv lungelidelse

Eng: STOP-COPD – Standard vs Targeted Oxygen Therapy Prehospital for Chronic Obstructive Pulmonary Disease

Erklæring fra forsøgspersonen:

Jeg har fået skriftlig og mundtlig information og jeg ved nok om formålet, metoden, fordele og ulemper til at sige ja til at deltage.

Jeg ved, at det er frivilligt at deltage, og at jeg altid kan trække mit samtykke tilbage uden at miste mine nuværende eller fremtidige rettigheder til behandling.

Jeg giver samtykke til, at deltage i forskningsprojektet, og har fået en kopi af dette samtykkeark samt en kopi af den skriftelige information om projektet til eget brug.

Forsøgspersonens navn: ____

Dato: _______________ Underskrift: ____

Ønsker du at blive informeret om forskningsprojektets resultat samt eventuelle konsekvenser for dig?: Ja:______ Nej:______

Hvis Ja, e-mail adresse resultaterne kan sendes til:____________________________________

Erklæring fra den samtykkeansvarlige:

Jeg erklære, at forsøgspersonen har modtaget mundtlig og skriftelig information om forsøget.

Efter min overbevisning er der givet tilstrækkelig information til, at der kan træffes beslutning om deltagelse i forsøget.

Samtykkeansvarliges navn: ____

Dato: _______________ Underskrift: ____

Projekt identifikation:

EU CTA nummer: 2022-502003-30-00

NATIONALT CENTER FOR ETIK

10. marts 2022

Dine rettigheder som forsøgsperson i forsøg med medicin

Hvis du er deltager i et klinisk forsøg med medicin, så er det vigtigt, at du er opmærksom på dine rettigheder. Dem kan du læse om på denne side.

- Din deltagelse i forskningsprojektet er helt frivillig og kan kun ske, efter du har fået både skriftlig og mundtlig information om forskningsprojektet og underskrevet samtykkeerklæringen.
- Du kan til enhver tid mundtligt, skriftligt eller ved anden klar tilkendegivelse trække dit samtykke til deltagelse tilbage og udtræde af forskningsprojektet. Såfremt du trækker dit samtykke tilbage, påvirker dette ikke din ret til nuværende eller fremtidig behandling eller andre rettigheder, som du måtte have.
- Du har ret til at tage et familiemedlem, en ven eller en bekendt med til informationssamtalen.
- Du har ret til betænkningstid, før du underskriver samtykkeerklæringen.
- Oplysninger om dine helbredsforhold, øvrige rent private forhold og andre fortrolige oplysninger om dig, som fremkommer i forbindelse med forskningsprojektet, er omfattet af tavshedspligt.
- Behandling af oplysninger om dig, herunder oplysninger om dine blodprøver og væv, sker efter reglerne i databeskyttelsesforordningen, databeskyttelsesloven samt sundhedsloven. Den dataansvarlige i forsøget skal orientere dig nærmere om dine rettigheder efter databeskyttelsesreglerne.
- Der er mulighed for at få aktindsigt i forsøgsprotokoller efter offentlighedslovens bestemmelser. Det vil sige, at du kan få adgang til at se alle papirer vedrørende forsøgets tilrettelæggelse, bortset fra de dele, som indeholder forretningshemmeligheder eller fortrolige oplysninger om andre.
- Der er mulighed for at klage og få erstatning efter reglerne i lov om klage- og erstatningsadgang inden for sundhedsvæsenet. Hvis der under forsøget skulle opstå en skade, kan du henvende dig til Patienterstatningen, se nærmere på www.patienterstatningen.dk
